# Insulin signalling in tanycytes gates hypothalamic insulin uptake and regulation of AgRP neuron activity

**DOI:** 10.1038/s42255-021-00499-0

**Published:** 2021-12-20

**Authors:** Marta Porniece Kumar, Anna Lena Cremer, Paul Klemm, Lukas Steuernagel, Sivaraj Sundaram, Alexander Jais, A. Christine Hausen, Jenkang Tao, Anna Secher, Thomas Åskov Pedersen, Markus Schwaninger, F. Thomas Wunderlich, Bradford B. Lowell, Heiko Backes, Jens C. Brüning

**Affiliations:** 1 Department of Neuronal Control of Metabolism, Max Planck Institute for Metabolism Research, Cologne, Germany; 2 Institute for Experimental and Clinical Pharmacology and Toxicology, University of Lübeck, Lübeck, Germany; 3 Helmholtz Institute for Metabolic, Obesity and Vascular Research (HI-MAG) of the Helmholtz Zentrum München at the University of Leipzig and University Hospital Leipzig, Leipzig, Germany; 4 Division of Endocrinology, Diabetes and Metabolism, Department of Medicine, Beth Israel Deaconess Medical Center, Harvard Medical School, Boston, MA USA; 5 Program in Neuroscience, Harvard Medical School, Boston, MA USA; 6 Global Drug Discovery, Novo Nordisk A/S, Måløv, Denmark; 7 Policlinic for Endocrinology, Diabetes and Preventive Medicine (CEDP), University Hospital Cologne, Cologne, Germany; 8 National Center for Diabetes Research (DZD), Neuherberg, Germany

**Keywords:** Blood-brain barrier, Hypothalamus, Metabolism, Hypothalamus

## Abstract

Insulin acts on neurons and glial cells to regulate systemic glucose metabolism and feeding. However, the mechanisms of insulin access in discrete brain regions are incompletely defined. Here we show that insulin receptors in tanycytes, but not in brain endothelial cells, are required to regulate insulin access to the hypothalamic arcuate nucleus. Mice lacking insulin receptors in tanycytes (IR^∆Tan^ mice) exhibit systemic insulin resistance, while displaying normal food intake and energy expenditure. Tanycytic insulin receptors are also necessary for the orexigenic effects of ghrelin, but not for the anorexic effects of leptin. IR^∆Tan^ mice exhibit increased agouti-related peptide (AgRP) neuronal activity, while displaying blunted AgRP neuronal adaptations to feeding-related stimuli. Lastly, a highly palatable food decreases tanycytic and arcuate nucleus insulin signalling to levels comparable to those seen in IR^∆Tan^ mice. These changes are rooted in modifications of cellular stress responses and of mitochondrial protein quality control in tanycytes. Conclusively, we reveal a critical role of tanycyte insulin receptors in gating feeding-state-dependent regulation of AgRP neurons and systemic insulin sensitivity, and show that insulin resistance in tanycytes contributes to the pleiotropic manifestations of obesity-associated insulin resistance.

## Abstract (web summary)

Tanycytic insulin receptors allow insulin access to the hypothalamic arcuate nucleus and are relevant for driving AgRP neuronal activity in response to feeding.

## Main

Beyond insulin’s pleiotropic effects in peripheral tissues, it also acts on neural circuits to control systemic metabolism^1^ via regulation of body weight as well as systemic fat and glucose metabolism^2–4^. Insulin activates insulin receptors (IRs) expressed in neurons and glial cells to provide a feedback signal instructing the brain about glucose availability of the organism. In the hypothalamus, insulin modifies excitability of neurons in several regions such as the arcuate nucleus (ARC), the ventromedial nucleus of the hypothalamus (VMH) and the lateral hypothalamus (LH)^5–7^. Specifically, insulin hyperpolarizes and inactivates AgRP/neuropeptide Y (NPY)-expressing neurons, contributing to the regulation of hepatic gluconeogenesis, glucose uptake in brown adipose tissue (BAT) as well as to the control of meal size^5,8–12^.

Yet, the molecular mechanisms underlying insulin access to these target cells remain incompletely defined. Early studies have shown that insulin crosses the blood–brain barrier (BBB) through active and saturable transport^12,13^. Kinetic studies on regional distribution of insulin in the brain on intravenous (i.v.) injection of radioactively labelled insulin indicated that the hypothalamus exhibits the highest insulin uptake rates compared with other brain regions^14^. However, which cell types, receptors or transporters are involved in this process is largely unknown^15^.

Recently, in vivo studies have demonstrated that insulin transport across the BBB can occur largely independent of the IR expression in endothelial cells^16^, while previous studies had reported that IR inactivation in endothelial cells leads to delayed insulin signalling in several brain regions thus causing mild obesity and systemic insulin resistance^17^. However, besides brain vasculature this animal model exhibited IR inactivation in the peripheral vasculature. Therefore, the specific role of endothelial cells of the BBB and other compartments of the BBB has not been fully elucidated.

In the mediobasal hypothalamus (MBH), barrier properties in addition to endothelial cells are governed by tanycytes^18^. Tanycytes are specialized radial glial cells, which line the third ventricle and regulate of broad range of hypothalamic functions^19^. The apical side of tanycytes in the median eminence is characterized by tight-junction complexes, thereby preventing passage of blood-borne molecules into the cerebrospinal fluid (CSF). On the basal side, tanycytes possess processes that contact fenestrated vessels in the median eminence and capillaries in neighbouring hypothalamic nuclei^20^. Previous studies have proposed that the main route for metabolic hormones to enter the brain is via the brain vasculature^12,21^. However, recent evidence indicates that hypothalamic tanycytes also contribute to transcytosis of peripheral hormones such as leptin and ghrelin across the median eminence and into the ARC^18,22–25^. In addition, tanycytes have nutrient-sensing properties and are involved in hormone release^26–30^. Moreover, conditional ablation of tanycytes in mice has further highlighted their role in regulating energy balance and fat metabolism^31^.

To define the specific role of IR-dependent signalling in different compartments of the BBB in vivo, we have generated mice with specific deletion of the IR in either brain vascular endothelial cells (BVECs) or in tanycytes. We demonstrated that IRs in tanycytes but not in BVECs are required to regulate insulin access to the ARC and thereby control regulation of AgRP neurons and systemic insulin sensitivity.

## Results

### IR inactivation in BBB compartments

To investigate the potential contribution of IR-dependent signalling in key components of the blood–ARC or blood–brain barrier, we employed two inducible genetic mouse models to specifically compromise expression of the IR either in tanycytes or in the brain microvasculature of mice.

**Fig. 1:**
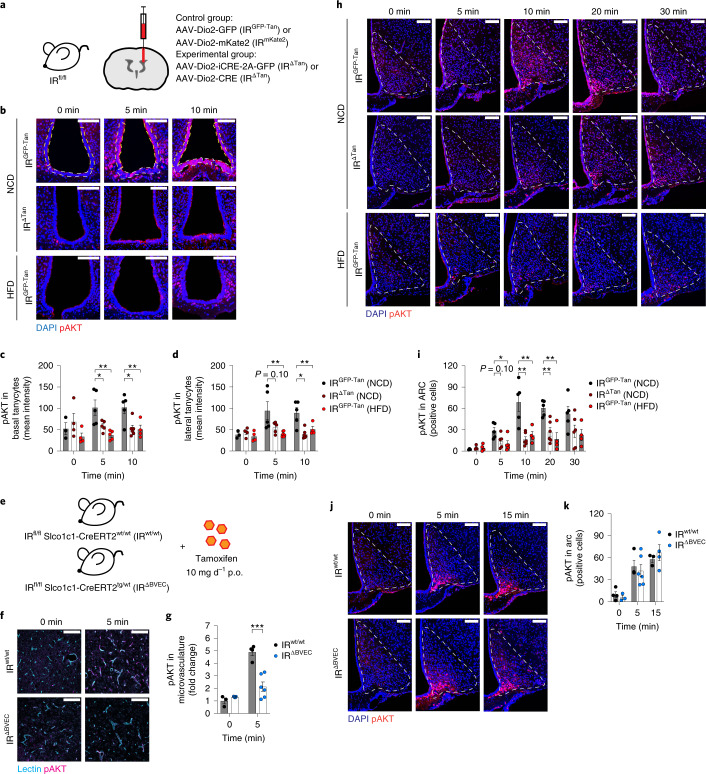
IR in tanycytes is necessary for insulin signalling in the ARC. **a**, Knockout strategy to inactivate IR specifically in tanycytes. Animals 10–12 weeks old received i.c.v. injection of either control virus AAV-Dio2-GFP or AAV-Dio2-mKate2 (IR^GFP-Tan^, IR^mKate2^) or Cre-recombinase-carrying virus AAV-Dio2-iCRE-GFP or AAV-Dio2-Cre (IR^ΔTan^). **b**, Representative images of pAKT signal in basal and lateral tanycytes of unstimulated (0 min) and 5 and 10 min post i.v. injection of 0.5 IU kg^−1^ insulin in NCD-fed control animals IR^GFP-Tan^ (NCD) (top panel), tanycyte-specific IR KO animals (IR^ΔTan^) (NCD) (middle panel) and HFD-fed control animals IR^GFP-Tan^ (HFD) (lower panel). pAKT was quantified in DAPI-positive tanycyte layer. White and yellow dashed lines indicate quantified ROI in basal and lateral tanycytes, respectively. **c**,**d**, Mean intensity of pAKT signal in DAPI-positive nuclei of basal (**c**) and lateral tanycytes (**d**). **c**, 5 min: *P*(IR^GFP-Tan^ (NCD) versus IR^ΔTan^ (NCD)) = 0.047, *P*(IR^GFP-Tan^ (NCD) versus IR^GFP-Tan^ (HFD)) = 0.0041; 10 min: *P*(IR^GFP-Tan^ (NCD) versus IR^ΔTan^ (NCD)) = 0.0144, *P*(IR^GFP-Tan^ (NCD) versus IR^GFP-Tan^ (HFD)) = 0.0205. **d**, *P*(IR^GFP-Tan^ (NCD) versus IR^ΔTan^ (NCD)) = 0.0994, *P*(IR^GFP-Tan^ (NCD) versus IR^GFP-Tan^ (HFD)) = 0.0151; 10 min: *P*(IR^GFP-Tan^ (NCD) versus IR^ΔTan^ (NCD)) = 0.0031, *P*(IR^GFP-Tan^ (NCD) versus IR^GFP-Tan^ (HFD)) = 0.0309. **b**–**d**, *n*(0 min) = 3/IR^GFP-Tan^ (NCD), 4/IR^ΔTan^ (NCD), 4/IR^GFP-Tan^ (HFD); *n*(5 min) = 5 mice per group; *n*(10 min) = 5/IR^GFP-Tan^ (NCD), 6/IR^ΔTan^ (NCD), 4/IR^GFP-Tan^ (HFD). **e**, Knockout strategy to inactivate IR in endothelial cells. The 10–12-week-old IR^fl/fl^ Slco1c1-CreERT2^wt/wt^ and IR^fl/fl^ Slco1c1-CreERT2^tg/wt^ littermates received tamoxifen (10 mg d^−1^, 5 d). **f**, Representative images of pAKT signal in lectin-positive brain cortices of unstimulated mice (0 min) and 5 min post i.v. injection of 0.5 IU kg^−1^ insulin in IR^wt/wt^ and IR^ΔBVEC^ mice. **g**, pAKT in lectin-positive microvessels, normalized to IR^wt/wt^ control animals. *P*(5 min) = 0.0007. **f**,**g**, *n*(0 min) = 3/IR^wt/wt^, 4/IR ^ΔBVEC^; *n*(5 min) = 4/IR^wt/wt^, 6/IR ^ΔBVEC^ (unpaired, two-sided Student’s *t*-test). **h**, Representative images of pAKT in the ARC of unstimulated mice (0 min) and 5, 10, 20 and 30 min post i.v. injection of 0.5 IU kg^−1^ insulin in NCD-fed control animals IR^GFP-Tan^ (NCD) (top panel), tanycyte-specific IR KO animals IR^ΔTan^ (NCD) (middle panel) and HFD-fed control animals IR^GFP-Tan^ (HFD) (lower panel). **i**, Quantification of pAKT-positive cells per ARC hemisphere. 5 min: *P*(IR^GFP-Tan^ (NCD) versus IR^ΔTan^ (NCD)) = 0.104, *P*(IR^GFP-Tan^ (NCD) versus IR^GFP-Tan^ (HFD)) = 0.0341; 10 min: *P*(IR^GFP-Tan^ (NCD) versus IR^ΔTan^ (NCD)) = 0.0014, *P*(IR^GFP-Tan^ (NCD) versus IR^GFP-Tan^ (HFD)) = 0.0069; 20 min: *P*(IR^GFP-Tan^ (NCD) versus IR^ΔTan^ (NCD)) = 0.007, *P*(IR^GFP-Tan^ (NCD) versus IR^GFP-Tan^ (HFD)) = 0.0023. **h**,**i**, *n*(0 min) = 3/IR^GFP-Tan^ (NCD), 4/IR^ΔTan^(NCD), 4/IR^GFP-Tan^ (HFD); *n*(5 min) = 5 mice per group; *n*(10 min, 20 min, 30 min) = 5/IR^GFP-Tan^ (NCD), 6/IR^ΔTan^(NCD), 4/IR^GFP-Tan^ (HFD). **j**, Representative images of pAKT in ARC of unstimulated mice (0 min) and 5 and 15 min post i.v. injection of 0.5 IU kg^−1^ insulin in tamoxifen-treated IR^wt/wt^ and IR^ΔBVEC^ mice. **k**, Quantification of pAKT-positive cells per hemisphere of ARC in treated IR^wt/wt^ and IR^ΔBVEC^ mice. **j**,**k**, *n*(0 min) = 4/IR^wt/wt^, 3/IR^ΔBVEC^; *n*(5 min) = 3/IR^wt/wt^, 5/IR^ΔBVEC^; *n*(5 min) = 3/IR^wt/wt^, 4/IR^ΔBVEC^. **c**,**d**,**i**, One-way ANOVA, Tukey post hoc test. Data are represented as the mean ± s.e.m. **b**,**f**,**h**,**k**, Scale bar, 100 µm. KO, knockout; p.o., per oral; ROI, region of interest. Source data

**Extended Data Fig. 1:**
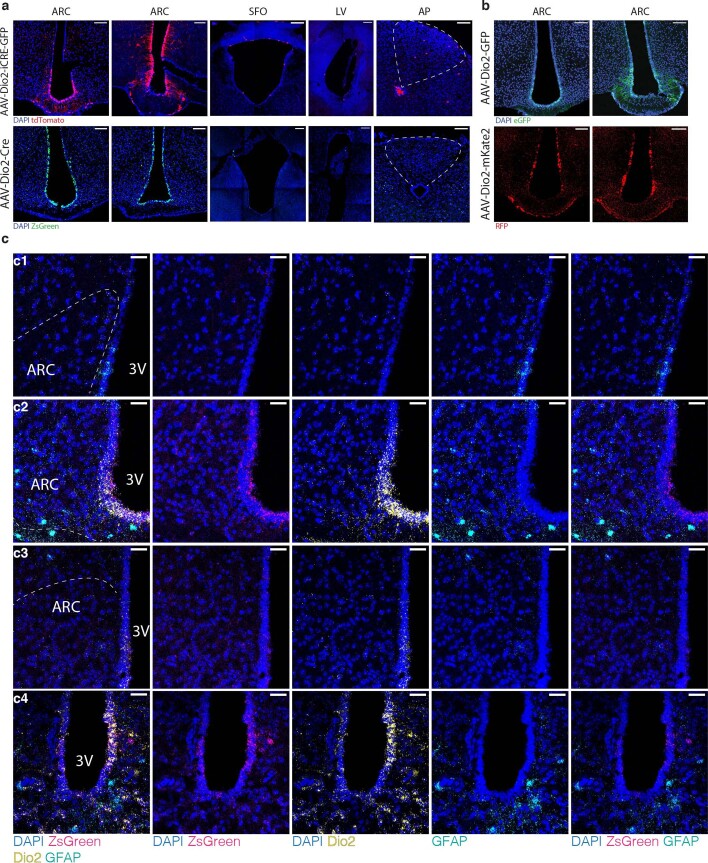
Validation of Cre-mediated recombination in tanycytes. a. Expression profiles of tanycytes specific Cre-expressing rAAVs in reporter mice across the brain. Expression of AAV-Dio2-iCRE-GFP was assessed in ROSA26|STOP|tdTomato^fl/fl^ reporter mice 3 weeks post i.c.v. injection in ARC, d3V, LV and AP. AAV-Dio2-Cre was injected i.c.v. in mice expressing Cre-dependent ZsGreen reporter from ROSA26 locus (Rosa26|STOP|ZsGreen^fl/fl^) and assessed in ARC, SFO, LV and AP. Virus expression and fluorescence of the ZsGreen reporter was assessed 3 – 4 weeks post injection in 3 – 5 mice. b. AAV-Dio2-GFP representative images were acquired from IR^GFP-Tan^ mice 6 weeks post injection. AAV-Dio2-mKate2 expression in tanycyte nuclei was verified in IR^fl/fl^ mice 3 weeks post injection. Because AAV-Dio2-mKate2 exhibited a weak fluorescent signal, it was amplified with immunohistochemistry staining for red fluorescent protein (RFP). All viruses were expressed in the lateral and basal tanycytes of the 3^rd^ ventricle. n = 4/group. c. Representative images of smISH of *ZsGreen* mRNA expression in mediobasal hypothalamus of Rosa26|STOP | ZsGreen^fl/fl^-mice 3 weeks post i.c.v. injection of AAV-Dio2-Cre virus in 5 mice. Expression of *Zsgreen* reporter gene was assessed in the anterior and posterior hypothalamus (c1 – medial dorsal, c2 – medial ventral, c3 – posterior dorsal, c4 – posterior ventral) by co-labelling for tanycyte- (*Dio2*) and astrocyte- (*GFAP*) specific genes. Dashed lines indicate the outline of the ARC. ARC – arcuate nucleus, d3V – dorsal third ventricle, SFO - subfornical organ, LV – lateral ventricle, AP – Area Postrema.

To inducibly inactivate the IR gene in tanycytes, we intracerebroventricularly (i.c.v.) injected mice homozygous for the lox-P-flanked *Insr* allele^32^ with a recombinant adeno-associated virus (rAAV) into the lateral ventricle expressing either Cre-recombinase and green fluorescent protein (GFP) (AAV-Dio2-iCRE-GFP) or GFP only (AAV-Dio2-GFP) under transcriptional control of the type II iodothyronine deiodinase (Dio2) promoter, thereby generating control (IR^GFP-Tan^) mice and experimental mice lacking the IR specifically in tanycytes (IR^ΔTan^)^30^ (Fig. 1a). Additionally, we generated viruses AAV-Dio2-mKate2 and AAV-Dio2-Cre, which exchange the GFP fluorophore in control virus to the higher wavelength fluorophore mKate2 or which lack GFP in Cre-recombinase-carrying virus (Extended Data Fig. 1a).

To validate the efficiency of Cre-mediated recombination, we i.c.v. injected AAV-Dio2-iCre-GFP and AAV-Dio2-Cre viruses into reporter mice, allowing for Cre-dependent expression of tdTomato (ROSA26|STOP|tDTomato^fl/fl^) or ZsGreen (ROSA26|STOP|ZsGreen^fl/fl^). Spontaneous fluorescence analysis 3 weeks post injection revealed strong tdTomato fluorescence and ZsGreen fluorescence rostral to caudal in basal and lateral tanycytes, while there was no signal observed in ependymal cells (Extended Data Fig. 1a). The expression of the AAV-Dio2-GFP and AAV-Dio2-mKate2 viruses was validated in IR^fl/fl^ mice 3 and 6 weeks post i.c.v. injection (Extended Data Fig. 1b).

Assessment of tDTomato expression in whole brain indicated only sparse signal at the dorsal portion of the third ventricle, the lateral ventricle and the area postrema, whereas AAV-Dio2-Cre-driven expression of ZsGreen reporter protein indicated even lower unspecific expression in these areas (Extended Data Fig. 1a). To assess in which cell types of the ventral third ventricle Cre-mediated recombination occurs, we employed RNAscope-based single-molecular fluorescence in situ hybridization (FISH) in AAV-Dio2-Cre-injected ROSA26|STOP|ZsGreen^fl/fl^ mice and analysed messenger RNA (mRNA) expression of *ZsGreen* and markers of tanycytes (*Dio2)* and astrocytes (*GFAP)*. This analysis revealed a clear overlap between *ZsGreen* and *Dio2* expression in basal tanycytes and ventro-lateral tanycytes of the anterior and posterior MBH (Extended Data Fig. 1c(2), (4)). Sparse *GFAP*-expressing cells were located only dorsally of the anterior (Extended Data Fig. 1c(1)), but not posterior, MBH (Extended Data Fig. 1c(3)), which did not overlap with *ZsGreen* mRNA expression. Together, these experiments indicated specific targeting of tanycytes in the third ventricle.

**Extended Data Fig. 2:**
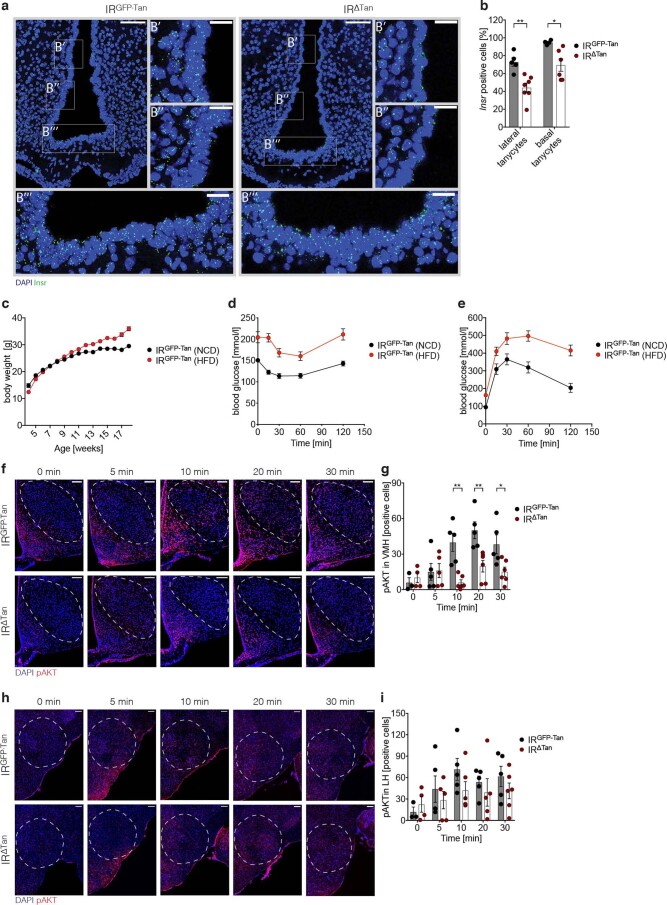
Verification of IR inactivation in tanycytes, HFD-induced insulin resistance in IR^GFP-Tan^-mice, and assessment of insulin signalling in VMH and LH of IR^ΔTan^-mice. a. Microphotographs of smFISH assessing *Insr* expression in lateral (B’, B’) and basal (B’’) tanycytes of IR^GFP-Tan^ and IR^ΔTan^ animals. b. Quantification of *Insr* positive cells in lateral and basal tanycytes. a-b. *p*(lateral) = 0.0026, *p*(basal) = 0.0186, n = 5/IR^GFP-Tan^, n = 7/IR^ΔTan^. c. Body weight of NCD and HFD fed IR^GFP-Tan^ animals. Animals were fed a HFD starting at 4 weeks of age, at 12 weeks mice received an i.c.v. injection with the AAV-Dio2-GFP virus. d. ITT of NCD and HFD fed IR^GFP-Tan^ animals (as in Fig. 3c). e. GTT of NCD and HFD fed IR^GFP-Tan^ animals (as in Fig. 3c). c-e. *n* = 13/IR^GFP-Tan^ (NCD), 14/IR^GFP-Tan^ (HFD). f-i. Representative images and quantification of pAKT immunostaining in ventromedial hypothalamus (g, h) and lateral hypothalamus (I, j) of unstimulated mice and 5, 10, 20 and 30 min post i.v. injection of 0.5 IU/kg insulin in IR^GFP-Tan^ (top panel) or IR^ΔTan^ (bottom panel). *n*(0 min) = 3/IR^GFP-Tan^ (NCD), 4/IR^ΔTan^(NCD); *n*(5 min) = 4/IR^GFP-Tan^ (NCD), 5/IR^ΔTan^ (NCD); *n*(10 min, 20 min, 30 min) = 5/IR^GFP-Tan^ (NCD), 6/IR^ΔTan^ (NCD). For VMH: *p*(10 min) = 0.0012, *p*(20 min) = 0.0058, *p*(30 min) = 0.023, a-b, f-i. unpaired, two-tailed Student’s *t*-test. Data are represented as the mean ± SEM. Scale bar: 100 µm. Source data

Next, we assessed *Insr* mRNA expression, employing RNAscope-based single-molecular FISH in control and IR^ΔTan^ mice. These experiments showed *Insr* expression in tanycytes and ependymal cells lining the third ventricle in control mice (Extended Data Fig. 2a,b), while IR^∆Tan^ mice exhibited a reduction in *Insr* mRNA expression in lateral (33%) and basal tanycytes (28%). The mild reduction in *Insr* mRNA expression can be explained by the fact that deletion of the lox-P-flanked exon 4 of *Insr* results in splicing of exons 3 and 5, which generates a premature stop codon after amino acid 308 (ref. ^32^).

To assess whether insulin signalling had been successfully perturbed in tanycytes of IR^∆Tan^ mice, we injected mice with insulin (i.v., 0.5 IU kg^−1^ body weight) and assessed phosphorylated AKT (pAKT) immunoreactivity in tanycytes. These analyses revealed that insulin treatment robustly induced AKT phosphorylation in tanycytes of control mice, and that this activation was largely diminished in tanycytes of IR^∆Tan^ mice (Fig. 1b–d).

Since obesity has been associated with insulin resistance in peripheral tissues such as liver, skeletal muscle and adipose tissue, as well as in the brain, we next aimed to investigate whether obesity also causes insulin resistance in tanycytes. Therefore, we assessed insulin’s ability to stimulate AKT phosphorylation in tanycytes of mice, which had been fed a high-fat diet (HFD) for 14 weeks and which exhibited increased body weight, adiposity, systemic insulin resistance and glucose intolerance (Extended Data Fig. 2c–e). Insulin’s ability to activate AKT phosphorylation in tanycytes was reduced in obese mice to similar extent as observed in IR^∆Tan^ mice (Fig. 1b–d).

To reduce expression of the IR specifically in the brain microvasculature, we crossed mice that carry the lox-P-flanked *Insr* allele with those expressing a tamoxifen-activatable Cre-recombinase (CreERT2) under control of thyroxine transporter Slco1c1-promoter (ref. ^33^), thus allowing for tamoxifen-inducible deletion of the IR in brain vascular endothelial cells (BVECs) in IR^fl/fl^ Slco1c1-CreERT2^tg/wt^ (IR^ΔBVEC^) mice, but not in control IR^fl/fl^ Slco1c1-CreERT2^wt/wt^ (IR^wt/wt^) mice (Fig. 1e).

**Extended Data Fig. 3:**
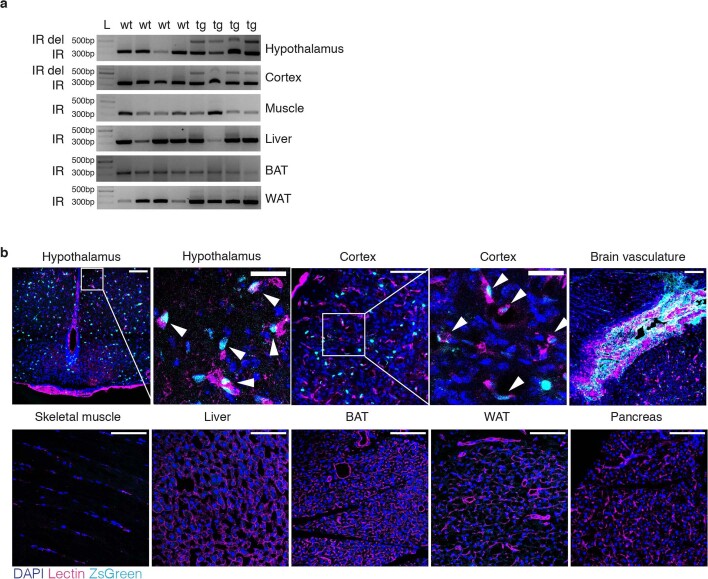
Validation of Cre-mediated recombination in brain vascular endothelial cells. a. PCR for wildtype insulin receptor gene (IR) and Exon 4 deleted insulin receptor allele (IR del) in hypothalamus, cortex, muscle, liver, BAT and WAT of IR^fl/fl^ Slco1c1-CreERT2^wt/wt^ (wt) and IR^fl/fl^ Slco1c1-CreERT2^tg/wt^ mice (tg) 8 weeks post tamoxifen treatment (n = 4/4). L – Gene Ruler 1 kb Plus DNA ladder. b. ZsGreen reporter protein expression in hypothalamus, cortex, brain vasculature, skeletal muscle, liver, BAT, WAT and pancreas of Rosa26|STOP | ZsGreen^fl/fl^ Slco1c1-CreERT2^tg/wt^ mice 2 weeks post tamoxifen treatment. Arrows indicate co-localized ZsGreen signal with lectin positive microvessels. Scale bar: 100 µm, scale bars in insets 30 µm. Source data

Validation of exon 4 deletion in IR^ΔBVEC^ mice and lack thereof in control mice using PCR indicated brain-specific (hypothalamus, cortex) inactivation of IR, while sparing skeletal muscle, liver, BAT and white adipose tissue (WAT) (Extended Data Fig. 3a). Further, we crossed Slco1c1-CreERT2 mice with ROSA26|STOP|ZsGreen^fl/fl^ mice (Extended Data Fig. 3b). At 2 weeks post tamoxifen treatment, ZsGreen was detected only in the brain, but not in skeletal muscle, liver, BAT, WAT or pancreas. Specifically, in brain ZsGreen was colocalized with lectin-positive microvessels in hypothalamus, cortex and brain macro-vasculature (Extended Data Fig. 3b).

Brain sections of IR^∆BVEC^ and control mice were further analysed for pAKT immunoreactivity and visualization of BVECs via lectin colabelling (Fig. 1f). While insulin rapidly induced AKT phosphorylation in BVECs of control mice, this response was largely attenuated in IR^∆BVEC^ mice (Fig. 1g).

### IR inactivation in tanycytes reduces insulin signalling in MBH neurons

Next, we analysed the ability of peripherally injected insulin to stimulate AKT phosphorylation in neurons of different regions of MBH of IR^∆Tan^, IR^∆BVEC^ and their control littermates. Knockout of IR in tanycytes resulted not only in decreased pAKT in tanycytes (Fig. 1b–d), but also in a notable reduction of pAKT immunoreactive cells in the ARC (Fig.1h,i) compared with control mice. Again, the magnitude of reduced insulin action in ARC neurons was comparable to what was observed in HFD-fed obese control mice (Fig. 1h,i).

We next analysed pAKT immunoreactivity in further regions of MBH. There was a clear reduction and delay in insulin-stimulated AKT phosphorylation in the VMH of IR^∆Tan^ compared with control mice (Extended Data Fig. 2f,i). In contrast, no difference could be observed in insulin-stimulated AKT phosphorylation in cells in the LH of mice of the different genotypes. Concurrently, we analysed insulin signalling in ARC of BVECs knockout mice. However, IR^∆BVEC^ mice showed unaltered pAKT-positive cells in the ARC (Fig. 1j,k).

Together, this analysis revealed a reduced and delayed response to insulin on IR deletion in tanycytes in key regions of MBH, which reside in close proximity to third ventricle, and in tanycytes, but not in more distal brain regions, such as the LH.

### Reduced MBH insulin uptake in IR^∆Tan^ mice

**Fig. 2:**
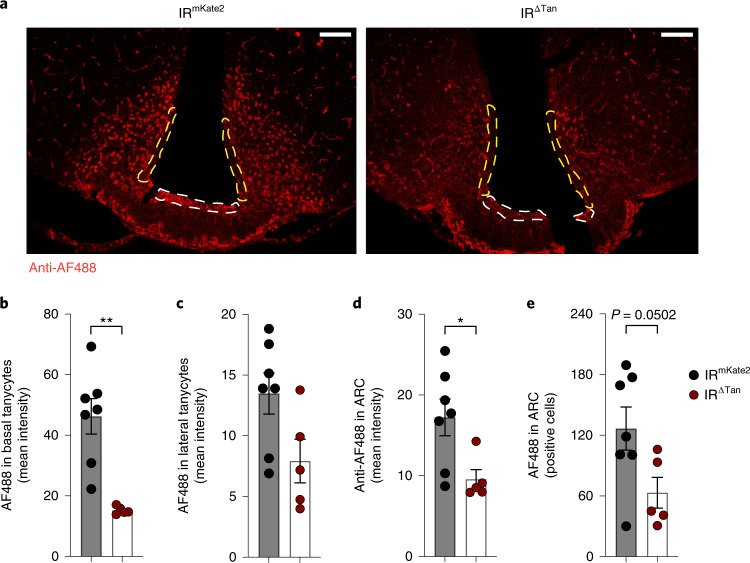
Insulin uptake in tanycytes and MBH is distorted in IR^∆Tan^ mice. **a**, Representative images of immunostaining for fluorescently labelled insulin (anti-Alexa-Fluor-488) 15 min post i.v. injection of 488-insulin (250 nmol kg^−1^) in IR^fl/fl^ mice, which received either AAV-Dio2-mKate2 (IR^mKate2^) control or Cre-recombinase-expressing AAV-Dio2-Cre virus (IR^ΔTan^). 488-insulin was quantified in DAPI-positive tanycyte layer. White and yellow dashed lines indicate quantified ROI in basal and lateral tanycytes, respectively. **b**–**d**, Mean intensity signal of Alexa-Fluor-488 fluorescent insulin in basal, *P* = 0.013, (**b**) and lateral (**c**) tanycytes and ARC, *P* = 0.0251, (**d**) of IR^mKate2^ and IR^ΔTan^ mice. **e**, Quantification of Alexa-Fluor-488-positive cells per hemisphere of ARC of IR^mKate2^ and IR^ΔTan^ mice, *P* = 0.0502. **b**,**d**,**e**, Unpaired, two-sided Student’s *t*-test. **a**–**e**, *n* = 7/IR^mKate2^, 5/IR^ΔTan^. Data are represented as the mean ± s.e.m. Scale bar, 100 µm. Source data

**Extended Data Fig. 4:**
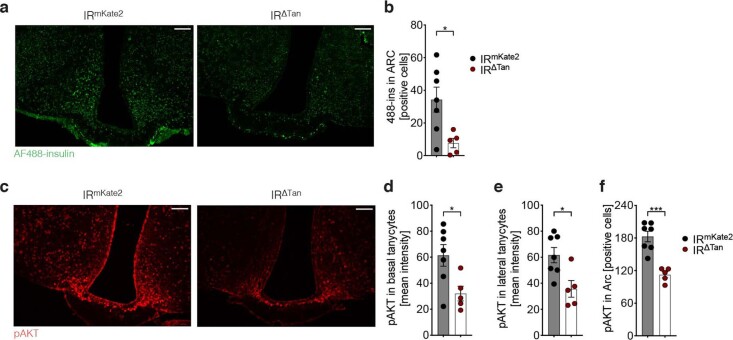
Reduced insulin uptake and insulin receptor signalling in tanycytes in IR^ΔTan^-mice. a, b. Representative images and quantification of spontaneous fluorescence of fluorescently labelled 488-insulin (250 nmol/kg) 15 min post i.p. injection in IR^mKate2^ and IR^ΔTan^ animals, p = 0.0187. c. Representative images of pAKT immunostaining 15 min post injection of 488-insulin (250 nmol/kg) in IR^mKate2^ and IR^ΔTan^ animal. d-f. Immunostaining of pAKT revealed reduced insulin signalling in basal, lateral tanycytes and in ARC, quantified as mean intensity and positive cells per ARC hemisphere, respectively p(basal) = 0.0237, p(lateral) = 0.0145, p(ARC) = 0.0002. a-f. *n* = 7/IR^mKate2^; *n* = 5/IR^ΔTan^, unpaired, two-tailed Student’s *t*-test. Data are represented as the mean ± SEM. Scale bar: 100 µm. Source data

Next, we investigated whether IR ablation in tanycytes may compromise insulin transport in tanycytes and the MBH. To this end, we peripherally administered fluorescently labelled insulin (AF488-insulin) and assessed AF488-insulin fluorescence in tanycytes (Extended Data Fig. 4a,b). These experiments revealed a clear reduction of fluorescent insulin staining intensity in tanycytes of IR^∆Tan^ compared with control mice (Fig. 2a–c). Interestingly, AF488-insulin immunoreactivity was not only reduced in tanycytes lining the ventricle but also in neurons of the ARC (Fig. 2a,d,e). Also, assessment of pAKT immunoreactivity on AF488-insulin injection confirmed a reduction of pAKT activation in tanycytes and ARC neurons of IR^∆Tan^ compared with control mice (Extended Data Fig. 4c–f).

### Altered glucose homoeostasis in IR^∆Tan^ mice

**Fig. 3:**
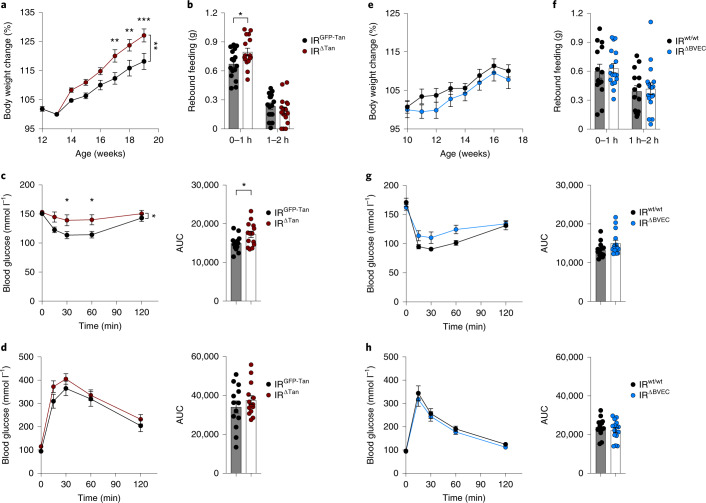
Tanycyte but not endothelial IR is necessary to maintain insulin sensitivity and rebound food intake. **a**,**e**, Body weight change of IR^GFP-Tan^ and IR^ΔTan^ mice normalized to 1 week post i.c.v. injection (13 weeks) (**a**) and IR^wt/wt^ and IR^ΔBVEC^ mice normalized to 1 week post tamoxifen treatment (11 weeks) (**e**), *n* = 13/IR^GFP-Tan^, IR^wt/wt^; *n* = 14/IR^ΔTan^; *n* = 15/IR^ΔBVEC^. **a**, Two-way ANOVA *P* = 0.0064, *P*(17 w) = 0.0055, *P*(18 w) = 0.0046, *P*(19 w) = 0.0008 (two-way ANOVA, Šídák post hoc test). **b**,**f**, Rebound food intake after 16-h fasting of IR^GFP-Tan^ and IR^ΔTan^ mice, *P*(0–1 h) = 0.028, (**b**) and IR^wt/wt^ and IR^ΔBVEC^ mice (**f**); *n* = 16/IR^GFP-Tan^, IR^ΔTan^; *n* = 17/IR^wt/wt^, IR^ΔBVEC^ (unpaired, two-sided Student’s *t*-test). **c**,**g**, Insulin tolerance test and area under the curve (AUC) of IR^GFP-Tan^ and IR^ΔTan^ mice (**c**) and IR^wt/wt^ and IR^ΔBVEC^ mice (**g**), *n* = 13/IR^GFP-Tan^, IR^wt/wt^; *n* = 14/IR^ΔTan^; *n* = 15/IR^ΔBVEC^, **c**, Two-way ANOVA *P* = 0.0334, *P*(30 min) = 0.0327, *P*(60 min) = 0.0318 (two-way ANOVA, Šídák post hoc test), *P*(AUC) = 0.031. **d**,**h**, Glucose tolerance test and AUC of IR^GFP-Tan^ and IR^ΔTan^ mice (**d**) and IR^wt/wt^ and IR^ΔBVEC^ mice (**h**), *n* = 13/IR^GFP-Tan^, IR^wt/wt^; *n* = 14/IR^ΔTan^; *n* = 15/IR^ΔBVEC^. **a**–**h**, Data are represented as the mean ± s.e.m. w, weeks. Source data

**Extended Data Fig. 5:**
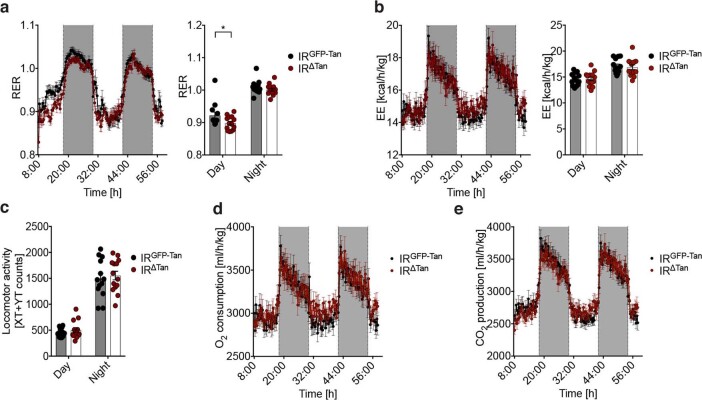
Indirect calorimetry of IR^ΔTan^-mice. a. Respiratory exchange ratio (RER) during day and night, p(Day) = 0.0448, unpaired, two-tailed Student’s t-test. b. Energy expenditure (EE) measurement during day and night. c. Mean locomotor activity during day and night. d. O_2_ consumption. e. CO_2_ production. a-e. n = 13/ IR^GFP-Tan^; n = 14/ IR^ΔTan^. Data are represented as the mean ± SEM. Source data

Insulin action in the ARC and specifically in AgRP neurons is critical for the coordinated regulation of food intake and hepatic gluconeogenesis^5,34,35^. Therefore, we next investigated systemic glucose homoeostasis in IR^∆Tan^ and IR^∆BVEC^ mice and their respective control littermates. At 3 weeks post i.c.v. administration of the respective AAVs, IR^∆Tan^ mice developed a slight increase in body weight compared with the littermate controls (Fig. 3a). While steady-state food intake was not altered in IR^∆Tan^ mice, they exhibited a notable increase during the first hour of re-feeding after fasting (Fig. 3b). Insulin and glucose tolerance tests 4 and 5 weeks post i.c.v. injection of recombinant AAVs (rAAVs) revealed an attenuated ability of exogenously applied insulin to lower blood glucose concentrations in IR^∆Tan^ mice compared with controls, whereas glucose tolerance remained unaltered (Fig. 3c,d). Indirect calorimetry of these animals showed only a minor reduction in respiratory exchange ratio during the light cycle, but no alterations in energy expenditure, oxygen consumption or basal locomotor activity (Extended Data Fig. 5). In contrast, IR^∆BVEC^ mice exhibited no differences in body weight, re-feeding responses, insulin sensitivity or glucose tolerance compared with their control littermates (Fig. 3e–h).

### IR^∆Tan^ mice exhibit systemic insulin resistance

**Fig. 4:**
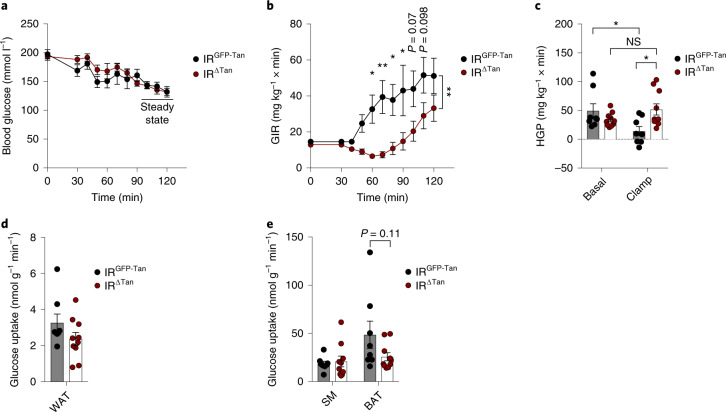
Regulation of glucose homoeostasis in tanycyte-specific IR knockout animals. **a**, Blood glucose levels during the hyperinsulinaemic–euglycaemic clamp period of IR^GFP-Tan^ and IR^ΔTan^ mice. **b**, GIR during the clamp period of IR^GFP-Tan^ and IR^ΔTan^ mice, two-way ANOVA *P* = 0.0048, *P*(60 min) = 0.0287, *P*(70 min) = 0.0027, *P*(80 min) = 0.0213, *P*(90 min) = 0.0131 (two-way ANOVA, Šídák post hoc test). **c**, HGP measured under basal and steady-state conditions during the clamp of IR^GFP-Tan^ and IR^ΔTan^ mice, *P*(IR^GFP-Tan^ (basal) versus IR^GFP-Tan^ (clamp)) = 0.0493, *P*(IR^GFP-Tan^ (clamp) versus IR^ΔTan^ (clamp)) = 0.0227, *P*(IR^ΔTan^ (basal) versus IR^ΔTan^ (clamp)) = 0.4341 (one-way ANOVA, Tukey post hoc test). **d**,**e**, Tissue-specific insulin-stimulated glucose uptake rates of IR^GFP-Tan^ and IR^ΔTan^ mice in WAT (**d**), skeletal muscle (SM) and BAT (**e**), **e** unpaired, two-sided Student’s *t*-test. **a**–**e**, *n* = 8/IR^GFP-Tan^; *n* = 10/IR^ΔTan^. Data are represented as the mean ± s.e.m. NS, not significant. * - P <0.05, ** - P <0.01, *** - P <0.001. Source data

Since IR^∆Tan^ mice exhibited an altered ability of insulin to activate AKT signalling in the ARC and demonstrated impaired systemic insulin sensitivity, we investigated which aspect of systemic glucose homoeostasis is impaired in these mice. To this end, we performed euglycemic, hyperinsulinaemic clamp studies in IR^∆Tan^ mice and their control littermates. Over a 120-min clamp period, IR^∆Tan^ mice required significantly lower glucose-infusion rate (GIR) from 30 min to 90 min to maintain euglycemia (Fig. 4a,b). Only from 100 to 120 min was there an increase in GIR, although they were not able to establish a steady state (Fig. 4b). Thus, these data suggest that tanycyte IR inactivation led to a reduced and delayed systemic response to insulin.

Assessment of the hepatic glucose production (HGP) in control mice revealed an effective suppression of gluconeogenesis in liver, while insulin’s ability to suppress HGP was attenuated in IR^∆Tan^ mice (Fig. 4c). Further analyses showed a tendency of reduced insulin-stimulated glucose uptake in BAT, but not in skeletal muscle or WAT (Fig. 4d,e).

### Insulin action in tanycytes regulates AgRP neuron activity

**Extended Data Fig. 6:**
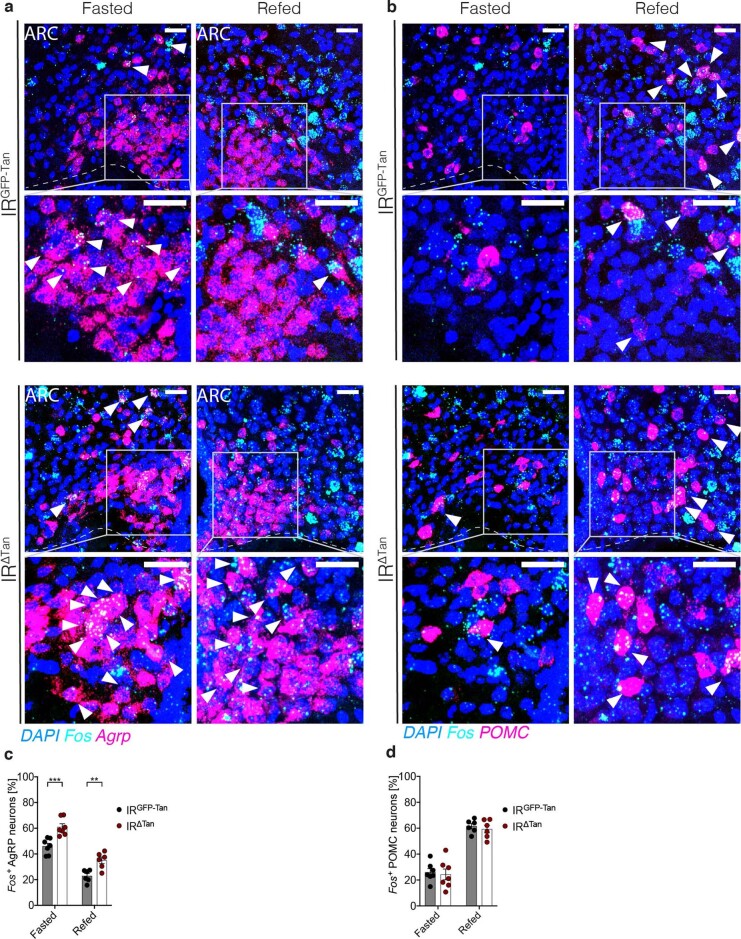
Tanycyte insulin receptor gates insulin dependent activity of AgRP neurons. a, c. *Fos* mRNA expression in *Agrp* (a) and *POMC* (c) mRNA expressing cells of the ARC (*Fos*^+^ positive cells per ARC hemisphere) in 16h fasted animals, which were further fasted for 1 h (Fasted) or refed for 1 h (Refed) of IR^GFP-Tan^ and IR^ΔTan^ mice. Scale bar: 100 µm, Scale bar in insets: 30 µm. b, d. Quantification of *Fos* and *Agrp* (b) and *POMC* (d) neurons (*Fos*^+^ positive cells per ARC hemisphere) in 16 h fasted animals, which were further fasted for 1 h (Fasted) or refed for 1 h (Refed) animals revealed increased AgRP neuron activation. For *Agrp* *p*(Fasted) = 0.0009, *p*(Refed) = 0.0036, For *POMC* *p*(Fasted) = 0.7436, *p*(Refed) = 0.5483, unpaired, two-sided Student’s *t*-test. Data are represented as the mean ± SEM. a, c. Dashed line represent the bottom of the Arcuate nucleus (ARC). Arrows indicate co-localized signal with DAPI and the respective neuronal marker (*Agrp* or *POMC*). *n* = 7/Fasted IR^GFP-Tan^ and IR^ΔTan^; *n* = 6/Refed IR^GFP-Tan^ and IR^ΔTan^. Source data

Given that the phenotype of IR^∆Tan^ mice largely resembled that of mice lacking insulin action in AgRP neurons, we sought to further investigate the regulation of AgRP and POMC neurons by means of *Fos* expression on fasting and 1 h after re-feeding. RNAscope-based assessment of *Fos* and *AgRP* mRNA expression showed that AgRP neuronal activity in the fasted state was suppressed by re-feeding in both IR^GFP-Tan^ and IR^ΔTan^ mice; however, *Fos* expression was significantly higher in IR^ΔTan^ mice compared with the GFP controls (46% versus 61% in fasted state, 23% versus 38% in re-fed state) (Extended Data Fig. 6a,b). *Fos* and *POMC* expression analysis in the same animals revealed an activation of POMC neurons on re-feeding, while there was no difference between the control and IR^∆Tan^ mice (Extended Data Fig. 6c,d).

**Extended Data Fig. 7:**
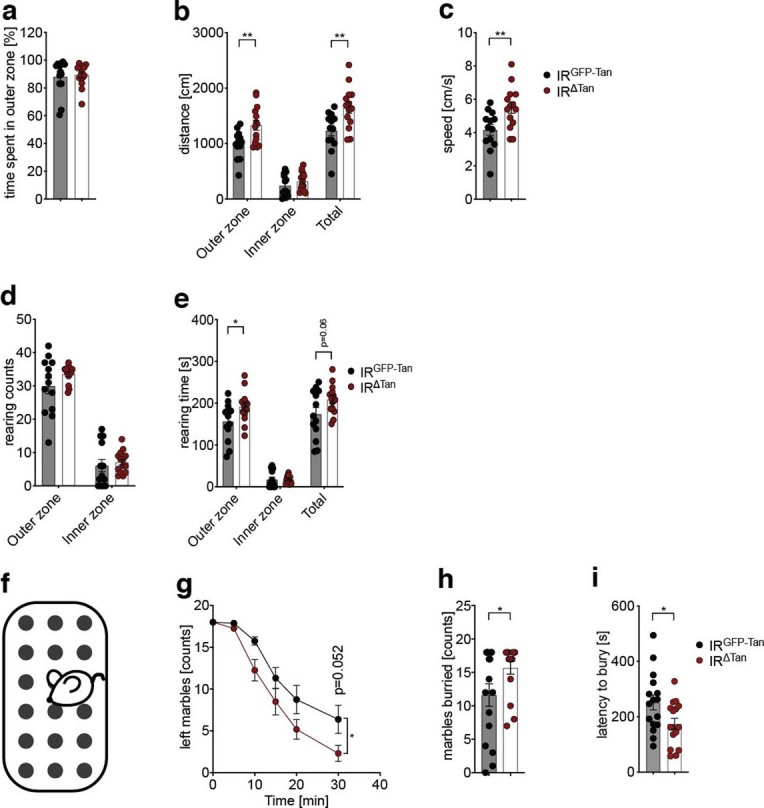
IR^∆Tan^-mice exhibit an increased propensity for repetitive and compulsive behaviours. a-e. Open field test (5 min) of IR^GFP-Tan^ and IR^ΔTan^ mice, n = 13/ IR^GFP-Tan^, n = 14/ IR^ΔTan^. a. Time spent in outer-zone during 5 min open field test. b. Distance travelled in inner, outer and in total during 5 min long open field test. p(outer zone) = 0.0069, p(total) = 0.006. c. Mean speed during open field test, p = 0.0087. d. Rearing counts during 5 min long open field test. e. Rearing time in inner, outer and in total during 5 min open field test p(outer zone) = 0.0366, p(total) = 0.0665. f-i. Layout of marble burying test (f). Burying behaviour of 18 marbles was determined over 30 min in IR^GFP-Tan^ and IR^ΔTan^ mice, n = 16/mice group. g. Burying behaviour of 18 marbles of IR^GFP-Tan^ and IR^ΔTan^ mice during 30 min long marble burying test, Two-way ANOVA p = 0.037, p(30 min) = 0.0519, (two way-ANOVA, Šídák post-hoc test). h. Count of total marbles buried after 30 min measurement, p = 0.0439. i. Latency to bury marbles, p = 0.0294. b, c, e, h, i. unpaired, two-tailed Student’s t-test. a – e, h – i. Data are represented as the mean ± SEM. Source data

Beyond orchestrating feeding behaviour and systemic insulin sensitivity, activation of AgRP neurons also drives food seeking and repetitive behaviours^36–38^. Thus, we further assessed acute locomotor activity, anxiety and the engagement in repetitive behaviours in random-fed IR^∆Tan^ mice compared with controls by employing open-field and marble burying tests (Extended Data Fig. 7). Although both controls and IR^ΔTan^ mice spent similar times in the outer zone and hence did not display differences in anxiogenic parameters (Extended Data Fig. 7a), during the 5-min exposure in the open-field arena IR^ΔTan^ animals covered longer distances in the outer zone and in both zones together, and had higher locomotor speed during exploration in comparison with control littermates (Extended Data Fig. 7b,c). Lastly, there was no difference between rearing counts; however, IR^ΔTan^ animals spend more time on rearing behaviours, suggesting increased exploration in IR^ΔTan^ mice (Extended Data Fig. 7d,e). Animals were further tested in a marble burying test. Here, IR^ΔTan^ mice buried significantly more marbles and initiated the burying behaviour earlier compared with littermate controls (Extended Data Fig. 7f–i). Thus, consistent with an attenuated ability to inhibit AgRP neurons, IR^∆Tan^ mice exhibited an increased propensity for repetitive, compulsive behaviour.

### Altered insulin-evoked brain network regulation in IR^∆Tan^ mice

**Fig. 5:**
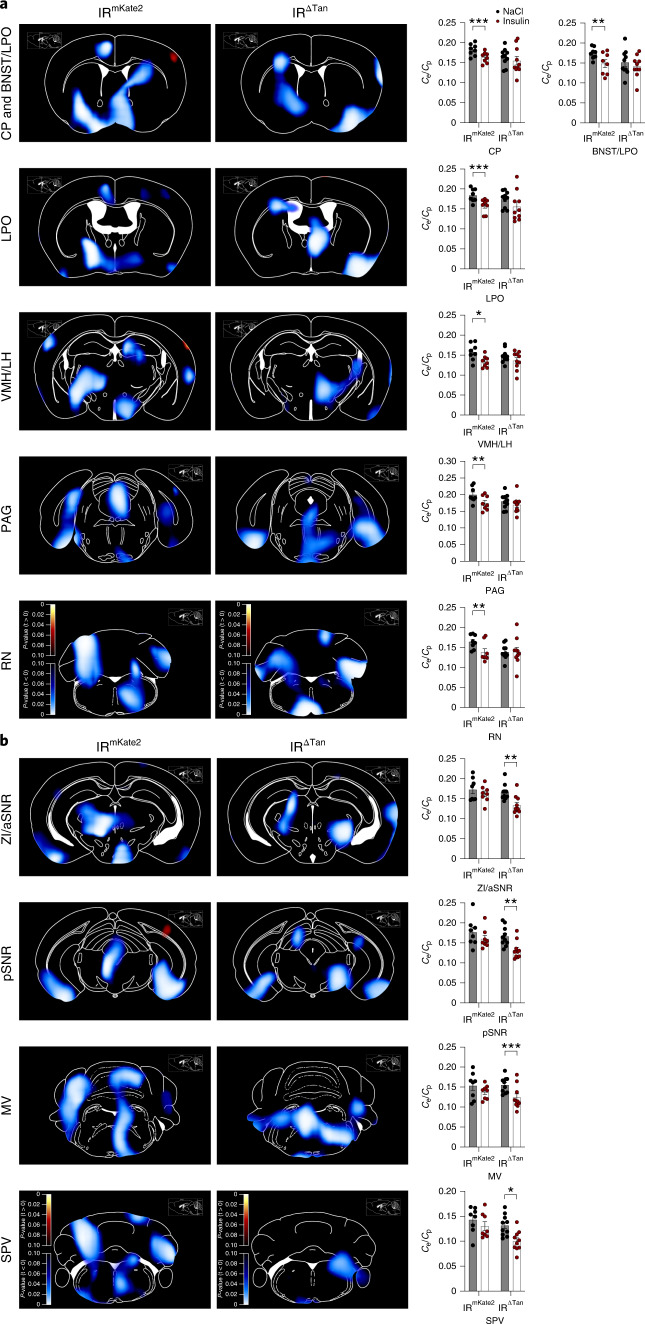
Altered insulin-evoked signalling in IR^ΔTan^ mice. **a**,**b**, Parametric maps of *P* values from paired *t*-test of differences in cumulative glucose metabolism over the recorded time determined by [18 F]FDG PET between insulin-stimulated (16-h fasted, i.p. 0.325 IU kg^−1^ insulin) and NaCl (0.9%)-injected, anaesthetized IR^mKate2^ and IR^ΔTan^ mice (*n* = 8/IR^mKate2^; *n* = 10/IR^ΔTan^). Brain regions that had significantly reduced cumulative glucose metabolism in IR^mKate2^ were not altered in IR^ΔTan^ mice (**a**), and brain regions that had significantly reduced signal in IR^Δtan^ remained unaltered in IR^mKate2^ (**b**). Blue colour scale indicates regions where metabolism at NaCl > insulin (inhibition on insulin injection). Sagittal reference image inserts show location of corresponding coronal plates^86^. *C*_e_/*C*_p_ is the ratio of tissue and blood glucose concentrations, a blood glucose level-insensitive measure for glucose metabolism. CP, caudate putamen; BNST/LPO, bed nucleus of stria terminalis/lateral preoptic area; PAG, periaqueductal grey; RN, reticular nucleus; ZI/aSNR, zona incerta/anterior substantia nigra; pSNR, posterior substantia nigra; MV, medial vestibular nucleus; SPV, spinal vestibular nucleus. Paired, two-sided Student’s *t*-test. **a**, For IR^mKate2^ *P*(CP) = 0.0007, *P*(BNST/LPO) = 0.0056, *P*(LPO) = 0.0003, *P*(VMH/LH) = 0.0109, *P*(PAG) = 0.0015, *P*(RN) = 0.0097, **b**, For IR^ΔTan^ *P*(ZI/aSNR) = 0.003, *P*(pSNR) = 0.0023, *P*(MV) = 0.0006, *P*(SPV) = 0.0105. Data are represented as the mean ± s.e.m. * - P <0.05, ** - P <0.01, *** - P <0.001. Source data

To assess potential brain-wide network alterations in IR^∆Tan^ mice, we performed 18F-fluorodeoxyglucose ([18 F]FDG)-based positron emission tomography (PET)-scanning in control and IR^∆Tan^ mice, comparing cumulative network changes in glucose metabolism between vehicle- and insulin-injected mice. Insulin injection resulted in substantially reduced brain glucose metabolism as a proxy of cumulative neuronal activity over the recording time in the caudate putamen, the bed nucleus of the stria terminalis, the MBH, the periaqueductal grey and in the brainstem of control mice (Fig. 5a). In contrast, insulin failed to suppress glucose metabolism in the same regions of IR^∆Tan^ mice (Fig. 5a). In contrast to control mice, insulin only modulated glucose uptake in the zona incerta, the substantia nigra and a distinct brainstem region of IR^∆Tan^ mice, regions that were not affected by insulin application in control animals (Fig. 5b). Interestingly, in control mice, but not in IR^∆Tan^ mice, there was a strong overlap in regions where insulin reduced glucose metabolism with regions that exhibit increased glucose metabolism on acute chemogenetic activation of AgRP neurons^8^. Similar to what was previously observed on chemogenetic activation of AgRP neurons, the insulin-evoked regulation exhibited often a lateralization in these regions. Collectively, these data indicate that in control animals insulin suppresses activity in brain networks targeted by AgRP neuron activation, and that this regulation was reduced in IR^∆Tan^ mice.

### Tanycyte IR signalling is dispensable for leptin action

**Extended Data Fig. 8:**
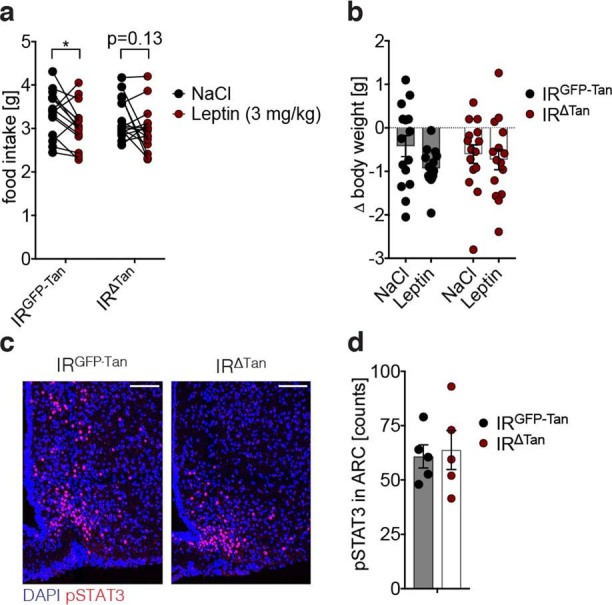
Tanycyte insulin receptor is not required for leptin access in ARC. a. Mean food intake during 3-day leptin (3 mg/kg) or NaCl (0.9%) administration in IR^GFP-Tan^ and IR^ΔTan^ mice, p(IR^GFP-Tan^) = 0.0364, p(IR^ΔTan^) = 0.1372, paired, two-sided Student’s t-test. b. Body weight change during 3-day leptin (3 mg/kg) or NaCl (0.9%) administration in IR^GFP-Tan^ and IRΔTan^ΔTan^ animals. c. Representative images of pSTAT3 in ARC 20 min post i.p. injection of 3 mg/kg leptin of IR^GFP-Tan^ and IR^ΔTan^ animals. Scale bar: 100 µm. c - d. Quantification of pSTAT3 positive cells per side of ARC, n = 5/group. a-b, d. Data are represented as the mean ± SEM. Source data

Tanycytes have been implicated to control leptin transport into the third ventricle, and leptin has been shown to contribute to the postprandial regulation of AgRP neuron activity^19,22^. Hence, we explored whether reduced insulin signalling in tanycytes possibly affects leptin action. Therefore, we compared systemic and cellular leptin sensitivity in IR^∆Tan^ and control mice. Three-day administration of leptin (intraperitoneal (i.p.), 3 mg kg^−1^) slightly suppressed food intake in 13 of 16 IR^GFP-Tan^ mice, in contrast to 4 of 16 IR^ΔTan^ mice (Extended Data Fig. 8a). Nevertheless, there was no overall difference in the ability of leptin to reduce body weight between the two groups of mice (Extended Data Fig. 8b). Moreover, when we assessed the ability of peripherally injected leptin (i.p., 3 mg kg^−1^) to activate Stat3-phosphorlyation in the ARC, quantification of pSTAT3 immunoreactive cells in the ARC revealed no notable differences in cellular leptin sensitivity between IR^GFP-Tan^ and IR^ΔTan^ animals (Extended Data Fig. 8c,d). Collectively, tanycyte IRs appear to be largely dispensable for controlling leptin access and action in the ARC.

### Tanycyte IR signalling regulates ghrelin action

**Fig. 6:**
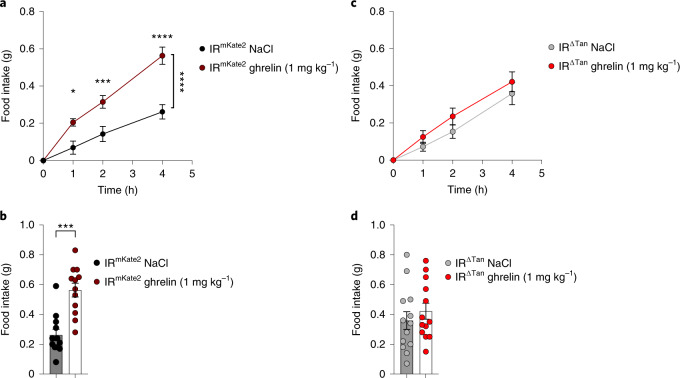
Tanycyte IR is required for ghrelin access in ARC. **a**, Food intake in random-fed IR^mKate2^ control animals after NaCl (0.9%) or ghrelin injection (i.p., 1 mg kg^−1^) over the 4-h measurement time. Two-way ANOVA *P* = 0.0002, *P*(1 h) = 0.013, *P*(2 h) = 0.0009, *P*(4 h) < 0.0001 (two-way ANOVA, Šídák post hoc test). **b**, Food intake 4 h post treatment with NaCl (0.9%) or ghrelin (i.p., 1 mg kg^−1^) in IR^mKate2^ control animals, *P* = 0.0001 (paired, two-tailed Student’s *t*-test). **a**,**b**, *n* = 12/IR^mKate2^. **c**, Mean food intake in random-fed IR^ΔTan^ animals after saline or ghrelin injection (i.p., 1 mg kg^−1^) over the 4-h measurement time. **d**, Food intake 4 h post treatment with NaCl or ghrelin injection (i.p., 1 mg kg^−1^) in IR^ΔTan^ animals. **c**,**d**, *n* = 13/IR^ΔTan^. **a**–**d**, Data are represented as the mean ± s.e.m. * - P <0.05, ** - P <0.01, *** - P <0.001, **** - P <0.0001. Source data

Fasting-induced increases in ghrelin exert critical feeding-regulatory functions via ghrelin action predominantly in AgRP neurons^39–41^. While the mechanisms of ghrelin transport have not been fully elucidated^42,43^, the feeding-regulatory action of peripherally applied ghrelin is blunted in obese, HFD-fed mice^44,45^. Thus, we aimed to investigate ghrelin action in IR^∆Tan^ mice compared with controls. Daytime injection of ghrelin in fed control mice resulted in a clear increase of feeding over 4 h following ghrelin administration, amounting to a 2.5-fold increase in food intake in control mice (Fig. 6a,b). In contrast, i.p. injection of ghrelin failed to increase food intake compared with vehicle-treated animals in IR^∆Tan^ mice (Fig. 6c,d). Collectively, ablation of IR action in tanycytes abrogates the feeding-regulatory effect of peripherally applied ghrelin.

### Altered AgRP neuron calcium dynamics in IR^∆Tan^ mice

**Extended Data Fig. 9:**
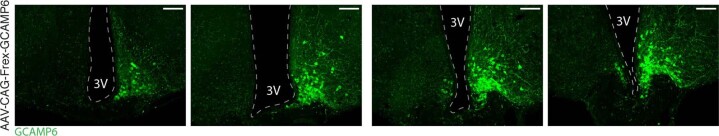
Validation of AAV-CAG-Frex-GCaMP6 expression in AgRP neurons. Coronal sections showing anterior to posterior GCaMP6 expression in the ARC of IR^fl/fl^ AgRP-p2a-Dre^tg/wt^ mice 3 weeks post unilateral injection. n = 2. Scale bar: 100 µm.

We investigated in vivo the dynamic regulation of AgRP neuron activity in awake freely moving IR^∆Tan^ mice and control littermates. To this end, we employed a dual-recombinase-based model^46^, which allows simultaneous Cre-dependent IR inactivation in tanycytes and Dre-dependent expression of the Ca^2+^sensor GCaMP6 in AgRP neurons. First, we generated mice, which express Dre recombinase from the endogenous AgRP locus. Next, we crossed IR^fl/fl^ mice with AgRP-p2a-Dre^tg/wt^ mice to ultimately generate IR^fl/fl^ Agrp-2a-Dre^tg/wt^, which would allow for Cre-recombinase-mediated inactivation of IR and Dre recombinase-mediated expression of GCaMP6. Briefly, we cloned an AAV (AAV-CAG-Frex-GCAMP6) where a CAG promoter drives expression of Frex-GCAMP6, an inverted GCaMP6s sequence flanked by two pairs of rox/mutant rox sites, which are recognized by Dre recombinase. Assessment of AAV-CAG-Frex-GCAMP6 expression 3 weeks post injection unilaterally in the ARC of IR^fl/fl^ Agrp-2a-Dre^tg/wt^ animals revealed a strong spontaneous fluorescence signal in the ARC, consistent with the localization of AgRP neurons (Extended Data Fig. 9).

**Fig. 7:**
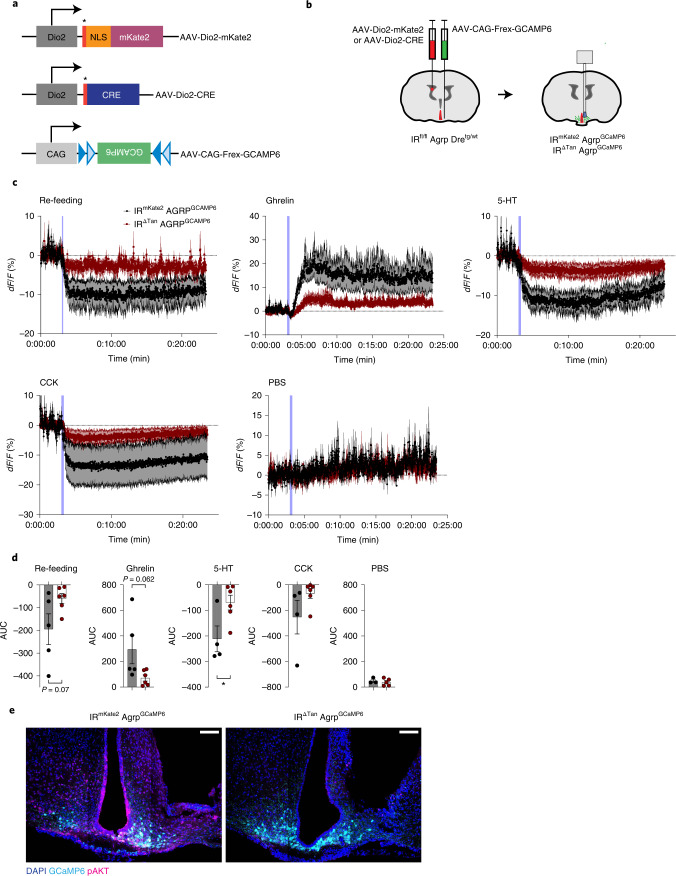
Altered AgRP neuron calcium dynamics in response to food and gut hormones in IR^∆Tan^ mice. **a**, Tanycyte- and AgRP neuron-specific rAAVs, which were employed to record AgRP neuron dynamics. NLS, nuclear localization signal; *Kozak sequence. **b**, Targeting strategy of tanycytes and AgRP to record AgRP neuron dynamics in mice with inactivated IR in tanycytes. At age 10 weeks IR^fl/fl^ Agrp-2a-Dre^tg/wt^ were injected with rAAV into the lateral ventricle expressing either Cre-recombinase (AAV-Dio2-CRE) or mKate2 fluorescent protein (AAV-Dio2-mKate2), and in the ARC unilaterally with an AAV virus carrying Dre-dependent, neuron-specific AAV-CAG-Frex-GCAMP6 flanked by two rox sites. **c**,**d**, Calcium signal traces (**c**) and AUC (**d**) from AgRP neurons in nonfasted IR^mKate2^ AgRP^GCaMP6^ and IR^∆Tan^ AgRP^GCaMP6^ mice treated with ghrelin (60 µg per mouse, i.p.) and in 16-h-fasted IR^mKate2^ AgRP^GCaMP6^ and IR^∆Tan^ AgRP^GCaMP6^ mice exposed to food pellet, treated with CCK (10 µg kg^−1^, i.p.), 5-HT (2 mg kg^−1^, i.p.) or PBS control (10 µl g^−1^ body weight, i.p.). Blue lines in the figure indicate the time of the intervention. Data are represented as the mean ± s.e.m. *n* = 3–6 mice per group. *P*(5-HT) = 0.030 (unpaired, two-tailed Student’s *t*-test (**d**)), *P ≤ 0.05. **e**, Representative images of GCaMP6 expression and pAKT signal in ARC 10 min post i.v. injection of insulin (0.5 IU kg^−1^) of IR^mKate2^ AgRP^GCaMP6^ and IR^∆Tan^ AgRP^GCaMP6^ mice at the end of the fibre photometry recordings, *n* = 3–6 mice per group. Scale bar, 100 µm. *dF*/*F*, represents the change in GCaMPs fluorescence from the mean level before the treatment. Source data

For experimental cohorts IR^fl/fl^ Agrp-2a-Dre^tg/wt^ animals, which received either AAV-Dio2-mKate2 or AAV-Dio2-CRE i.c.v., were also injected with AAV-CAG-Frex-GCAMP6 unilaterally in ARC, generating control (IR^mKate2^ AgRP^GCaMP6^) and experimental (IR^∆Tan^ AgRP^GCaMP6^) mice, which express GCaMP6 in AgRP neurons, but lack IR expression in tanycytes. The expression of GCaMP6 was further validated post hoc in insulin-stimulated (i.v., 10 min, 0.5 IU kg^−1^ insulin) IR^mKate2^ AgRP^GCaMP6^ and IR^∆Tan^ AgRP^GCaMP6^ mice. This analysis revealed a strong pAKT signal in tanycytes and GCAMP6-expressing neurons in ARC of control mice; however, experimental animals exhibited reduced pAKT immunoreactivity in tanycytes and in the ARC (Fig. 7e).

An optical fibre was implanted in the ARC above the site of the AAV-CAG-Frex-GCAMP6 injection, and 4 weeks post surgery recordings of GCaMP6 signals were performed.

Because gastrointestinal and hunger signals originating from gut and re-feeding are the most potent acute modulators of AgRP neuron activity, we investigated whether these responses are altered in IR^∆Tan^ AgRP^GCaMP6^ mice. To this end, animals were challenged with several gut-secreted hormones and food: (1) re-feeding of fasted animals; (2) serotonin (5-HT) injection (2 mg kg^−1^), which transiently inhibits AgRP neurons; (3) cholecystokinin (CCK) injection (10 µg kg^−1^), which transiently inhibits AgRP neurons; and (4) ghrelin (60 µg per mouse) as a potent activator of AgRP neurons^47–49^.

In fasted control animals presentation of food resulted in rapid inhibition of AgRP neurons; however, this response was virtually abolished in IR^∆Tan^ AgRP^GCaMP6^ mice (Fig. 7c,d). Similarly, while ghrelin injection in fed control mice evoked a rapid activation of AgRP neurons, this was largely attenuated in IR^∆Tan^ AgRP^GCaMP6^ mice (Fig. 7c,d). Of note, also the ability of 5-HT and CCK to suppress AgRP neuron activity was diminished in IR^∆Tan^ AgRP^GCaMP6^ compared with AgRP^GCaMP6^ mice (Fig. 7c,d). Taken together, these experiments clearly reveal that IR signalling in tanycytes represents a prerequisite for the feeding-state-dependent, dynamic regulation of AgRP neuron activity.

### Altered mitochondrial quality control in tanycytes of IR^∆Tan^ mice

**Fig. 8:**
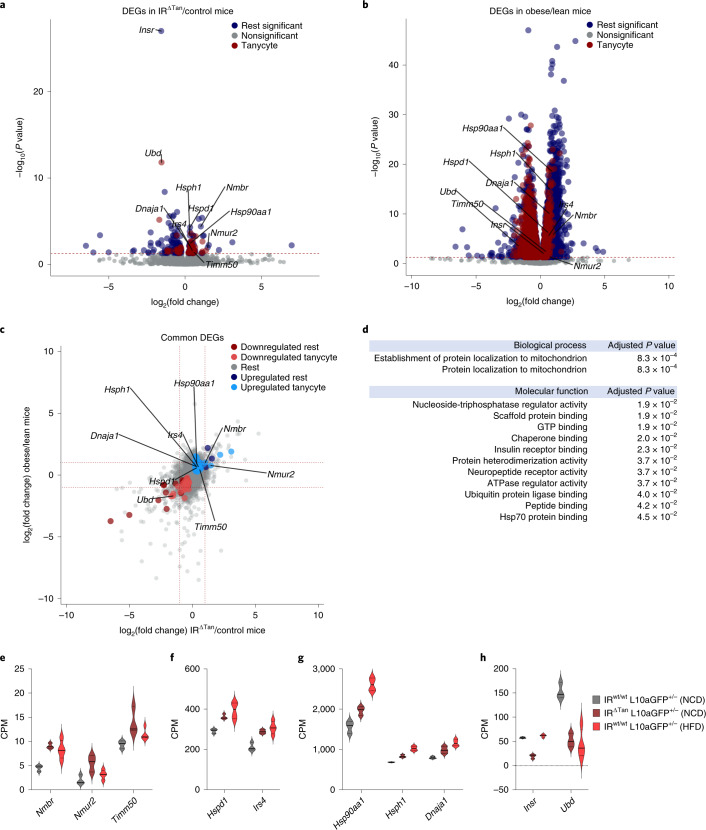
TRAP-based RNA sequencing identifies coordinated regulation of mitochondrial quality control. **a**,**b**, Volcano plots of nonsignificant genes (Nonsignificant, adjusted *P* > 0.05) and significant (adjusted *P* < 0.05) tanycyte-unrelated (Rest significant) and tanycyte-related (Tanycyte) genes in NCD control/IR^ΔTan^ (**a**) and NCD control/HFD control (**b**), shown as DEGs. **c**, Overlap analysis of DEGs in tanycytes of both comparisons (coloured region) revealed 60 out of 74 and 28 out of 50 tanycyte-specific upregulated and downregulated genes, respectively. **d**, GO analysis revealed upregulated biological processes and molecular functions in tanycytes of NCD control/IR^ΔTan^ versus NCD control/HFD control. *P* values are FDR-adjusted using Benjamini–Hochberg correction. **a**,**b**, Significantly differentially enriched transcripts (*P* ≤ 0.05) are indicated in the coloured region (*P* values were FDR-adjusted for multiple comparisons using Wald test). Red dashed line indicates the significance level (adjusted *P* < 0.05). Highlighted genes were selected from upregulated GO terms: protein localization to mitochondrion (*Timm50*, *Dnaja1*, *Hsp90aa1*, *Hspd1*, *Hsph1*), neuropeptide receptor activity (*Nmbr*, *Nmur2*), IR binding (*Irs4*). **c**, Red dashed line indicates the log_2_(fold change) = ±1. **e**–**h**, Expression values of selected upregulated genes with low (**e**), medium (**f**) or high (**g**) expression and of selected downregulated (**h**) genes. *n* = 3/NCD control mice and NCD IR^ΔTan^, *n* = 4/HFD control. CPM, counts per million reads; FDR, false discovery rate. Source data

**Extended Data Fig. 10:**
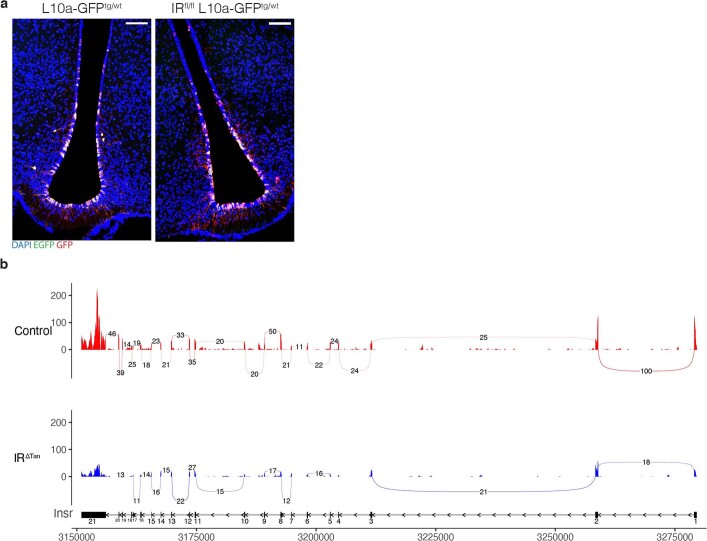
Validation of L10a-GFP tagged ribosome expression and Cre-recombinase mediated splicing of Exon 4 of IR in IR^∆Tan^-mice. a. Expression of GFP-tagged ribosomes in control L10a-GFP^tg/wt^ and experimental IR^fl/fl^ L10a-GFP^tg/wt^-mice 3 weeks post i.c.v. injection of AAV-Dio2-Cre virus. Endogenous GFP (EGFP, green) signal was amplified via immunostaining (GFP, red). n = 3/group. Scale: 100 µm. b. ggShashimi visualization of splicing events and read coverage of insulin receptor gene Insr (Chromosome 8: 3,172,061-3,329,617) by displaying exon spanning reads in pulldowns of L10a-GFP^tg/wt^ (control) and IR^fl/fl^ L10a-GFP^tg/wt^ (IR^ΔTan^) mice. For visual clarity, we require a minimum of 10 exon spanning reads to be displayed. The plots show a splicing event in exon 4 of the IR^ΔTan^ condition as well as a lower read coverage compared to wild-type.

Given the comparable development of insulin resistance and impaired action of insulin and ghrelin in the ARC of IR^∆Tan^ and obese, HFD-fed mice, we next aimed at comparing molecular changes occurring in tanycytes of both mouse models. To this end, we crossed IR^fl/fl^ mice with mice allowing for Cre-dependent expression of the ribosomal protein L10a fused to GFP (ROSA26lSTOPlL10a-GFP). Resulting IR^fl/fl^ ROSA26lSTOPlL10a-GFP^+/−^ or IR^wt/wt^ ROSA26lSTOPlL10-GFP^+/−^ mice received an i.c.v. injection of AAV-Dio2-Cre, resulting in tanycyte-specific expression of L10a-GFP in the presence or absence of tanycyte IR expression (IR^wt/wt^ L10a-GFP^+/−^ versus IR^∆Tan^ L10a-GFP^+/−^). GFP immunohistochemistry revealed tanycyte-specific expression of L10a-GFP (Extended Data Fig. 10). In parallel, control mice expressing L10a-GFP in tanycytes were exposed to HFD feeding for 12 weeks. At the age of 16 weeks, animals were killed and ribosomes from L10a-GFP-expressing tanycytes were immunoprecipitated with an anti-GFP antiserum, and ribosome-associated RNA was extracted and subjected to deep mRNA sequencing. In parallel, input hypothalamic RNA was subjected to mRNA sequencing. Comparing the ratio of reads detected in the immunoprecipitation versus input allowed for identification of tanycyte-specifically expressed genes, as well as their differential regulation in IR^∆Tan^ mice under normal chow diet (NCD) feeding and in control mice under NCD and HFD feeding. Investigating the expression of *Insr* in immunoprecipitations of NCD-fed IR^∆Tan^ and control mice revealed reduced *Insr* expression (Fig. 8a,h). Moreover, analysing specifically exon-spanning reads in the *Insr* gene revealed efficient exclusion of exon 4 from the targeted allele in immunoprecipitations of IR^∆Tan^ mice (Extended Data Fig. 10b). Next, we overlaid the datasets to identify commonly regulated pathways of insulin resistance in IR^∆Tan^ mice and diet-induced obesity. Additionally, we determined tanycyte-related genes by extracting differentially upregulated genes between tanycyte ribosomal pulldown (immunoprecipitation, IP) and the hypothalamic background (input) per sample (adjusted *P* ≤ 0.05, log_2_(fold change) > 0). This analysis revealed 124 commonly differentially expressed genes (DEGs) (adjusted *P* < 0.05 IR^∆Tan^/NCD control and adjusted *P* < 0.05 HFD control/NCD control). Of 74 upregulated and 50 downregulated genes, 60 and 28, respectively, were tanycyte enriched (Fig. 8c). Gene ontology (GO) analysis of commonly significantly upregulated genes identified mitochondrial protein localization as a significantly enriched biological process. Additionally, the upregulated genes were classified to have molecular function of Hsp70, IR and scaffold protein binding and neuropeptide receptor activity, among others (Fig. 8d). Interestingly, genes belonging to protein localization to mitochondrion GO terms included the mitochondrial import inner membrane translocase Tim50 (*Timm50*) and heat shock proteins Hsp40 (*Dnaja1*), Hsp60 (*Hspd1*), Hsp90a (*Hsp90aa1*) and Hsp110 (*Hsph1*) (Fig. 8e–h). Together, these experiments indicate an increased stress response and dysregulated control of mitochondrial function in IR^∆Tan^ mice and in the obese mouse model.

## Discussion

Brain vascular endothelial cells have long been thought to contain transporters that translocate insulin from blood to brain in a saturable manner^12,13,50–52^. It has been assumed to be the same protein that functions as its signalling receptor^53,54^. However, pharmacokinetic studies in brain- and tissue-specific IR knockout mice suggest that insulin transport across the BBB can occur independent of the IR^16^. Extensive investigations of BVEC involvement in insulin access in the brain have largely overshadowed the fact that the MBH does not rely on tight BBB function^17,52,55,56^. This is also supported by early kinetic studies, indicating a twice as high insulin uptake in the hypothalamus than in other brain regions^14^. In light of discoveries that tanycytes shuttle circulating metabolic signals, we investigated whether tanycytes could be involved in control of insulin access to the ARC^18^. Our data indicate that in the MBH insulin access and regulation of systemic insulin sensitivity rely on IR expression in tanycytes, whereas insulin action in BVECs is dispensable for the regulation of systemic metabolism.

At the MBH fenestrated vessels and tanycytes form the blood–CSF and blood–arcuate interfaces^57^. Here tanycytes have a unique localization, which allows them to integrate peripheral inputs^22,42,58^. We demonstrate that after peripheral injection of fluorescently labelled insulin, a clear labelling of basal and lateral tanycytes as well as distinct cell nuclei of ARC was observed, and this signal was significantly reduced in IR^∆Tan^ mice. Regarding possible mechanisms to enter tanycytes and MBH, it had been shown that leptin transport involves endocytosis and transcytosis mechanisms of tanycytes^22,59^. Leptin is internalized through clathrin-coated vesicles at tanycyte end-feet and transported to tanycyte cell bodies^22^. Although it was reported that this mechanism relies on leptin receptor (LepR) expression, other studies had failed to detect LepR in tanycytes and thus hypothalamic leptin signalling may also involve tanycyte-independent processes^25,60^. In contrast, tanycytes clearly express IRs, but whether insulin transport directly involves IR-mediated transcytosis remains to be elucidated.

In recent years tanycytes have been implicated in regulation of systemic metabolism^29,31^. Tanycyte-derived Fgf21 is necessary for its central action to regulate lipid metabolism in subcutaneoeus WAT and liver^29^. Similarly, application of a single dose of Fgf1 leads to long-lasting reversal of diabetes in mice and rats^61^, and the primary Fgf1-responsive cell types include tanycytes^62^. Importantly, inducible ablation of β-tanycytes increased adiposity, rebound food intake after fasting and systemic insulin resistance without body weight changes, and enhanced fat accumulation at thermoneutrality^31^. Our findings align with the aforementioned study, since IR^∆Tan^ mice exhibited decreased insulin sensitivity and increased food intake after fasting, accompanied with unaltered ad libitum food intake, intact hypothalamic leptin sensitivity and only a mild body weight increase. The fact that IR^∆Tan^ mice largely phenocopy the effects of hypothalamic tanycyte ablation thus clearly further underlines the fundamental dependence of tanycytes on IR expression and function.

Interestingly, the phenotype of IR^∆Tan^ mice mimics that of mice lacking the IR in AgRP neurons^1,5^. Here, the failure of IR^∆Tan^ mice to efficiently suppress HGP pointed towards a disinhibition of AgRP neurons, which is substantiated by smFISH experiments revealing increased *Fos* expression in these cells. In addition, ghrelin, which acts predominantly via AgRP neurons, exhibited a blunted ability to induce feeding in IR^∆Tan^ mice. Lack of ghrelin-induced food intake in IR^∆Tan^ mice demonstrated a profound resistance towards peripherally applied ghrelin, which is a hallmark of dysregulated NPY/AgRP circuits^44^. Lastly, the PET imaging of IR^∆Tan^ mice exhibited a reduced ability of peripherally applied insulin to inhibit glucose metabolism in the MBH, the bed nucleus of the stria terminalis and the periaqueductal grey, which all receive prominent projections from AgRP neurons, and the activation of these regions has been directly linked to AgRP neuron activation^8,63^. In addition, IR^∆Tan^ mice displayed insulin-sensitive regions, zona incerta and anterior to posterior substantia nigra, which do not receive direct input from AgRP neurons, but express IRs and have been implicated in regulation of hedonic and reward-related aspects of feeding^64–66^. The mechanisms of increased insulin sensitivity in these regions remain unclear at this point and deserve further attention. Nevertheless, our study suggests that functional tanycyte IR is indispensable for insulin access in the ARC, for regulation of systemic insulin resistance and for AgRP neurons to mediate their action to downstream regions in the brain. Of note, our Ca^2+^ imaging analysis revealed a decreased ability of AgRP neurons to adapt their activity acutely in response not only to re-feeding and ghrelin, but also to 5-HT and CCK, in IR^∆Tan^ AgRP^GCaMP6^ mice. This points to a fundamental change in dynamic AgRP neuron regulation in the absence of tanycyte IR signalling, the underlying mechanism for which requires further investigation. The fact that already the rapid re-feeding-induced inhibition of AgRP neurons is clearly blunted points to the possibility that the chronic lack of insulin action in tanycytes may have caused more profound alterations in AgRP neuron network connectivity and/or cellular regulation, which we are currently investigating in follow-up studies. What appears striking, however, is the profound effect of tanycyte IR signalling on AgRP neuron regulation, while POMC neuron regulation appears to remain largely intact. These findings are consistent with a recent study revealing that tanycyte protrusions contact more frequently NPY/AgRP neurons than POMC neurons^67^. Thus, besides directly regulating insulin access to AgRP neurons, insulin signalling may alter more fundamental aspects of tanycyte functions, the functional consequences of which depend on the cell types predominantly regulated by tanycytes, that is, AgRP/NPY neurons.

Interestingly, when assessing insulin signalling in obese and insulin-resistant animals, we observed a similar impairment of insulin action in tanycytes and in ARC cells as in IR^∆Tan^ mice. These experiments clearly point towards impaired insulin action in tanycytes of obese mice, and further suggest an unexplored relevance of tanycyte insulin resistance in the manifestation of obesity-associated phenotypes. Of note, ribosome profiling of tanycytes in HFD-fed mice revealed unaltered expression of the IR in tanycytes compared with NCD-fed lean control animals. Thus, insulin resistance in tanycytes in obesity likely results at a level downstream of the IR as extensively documented in peripheral tissues of insulin resistance mouse models and humans^68^. Candidate pathways include inflammatory responses, lipotoxicity and de-regulated endoplasmic reticulum-stress signalling^69^.

RNA sequencing of the translatome of NCD-fed control, NCD-fed IR^∆Tan^ and HFD-fed control mice indicated that IR knockout and diet-induced insulin resistance exhibited fundamental changes in genes related to protein localization to mitochondria and heat shock protein binding, and that are part of a mitochondrial stress response. These experiments point towards coordinately dysregulated control of mitochondrial function in mice lacking the IR in tanycytes and obese mouse models. Directly investigating the role of mitochondrial function in tanycytes may help to identify novel targets to indirectly modulate neuronal function in the MBH.

Finally, our study provides a potential explanation for recent findings of AgRP neuron regulation in obesity. A recent study has revealed that HFD feeding devaluates NCD intake and shifts intake towards HFD consumption, which is encoded at the level of hypothalamic AgRP neurons and mesolimbic dopamine signalling, although the alterations in AgRP neuron activity observed after HFD exposure remained mechanistically unexplained in this study^70^. The observed changes in Ca^2+^ dynamics on loss of IR signalling in tanycytes of lean mice largely resemble those observed in HFD-fed mice. Thus, HFD-induced insulin resistance in tanycytes may link to NCD devaluation in obesity.

Importantly, the hereto reported function of tanycyte IR signalling to gate ARC neuronal responses is well in line with data on the regulation of brain insulin access and action in lean subjects and humans with obesity. In healthy humans insulin concentrations in CSF are correlated with plasma insulin levels^71^. However, as plasma insulin levels increase with body fat mass, waist circumference, hip circumference and the degree of insulin resistance, the CSF/plasma insulin ratio is negatively correlated with the same parameters^72^. This can be partially attributed to reduced entry of insulin into the brain/CSF compartment^72^. In healthy and mostly normal weight individuals intranasal insulin activates occipital regions, prefrontal cortex and hypothalamus^73^. Randomized clinical trials in lean men have shown that intranasal insulin administration, which circumvents peripheral effects of insulin and thus ensures higher concentration in the brain, improved whole body insulin sensitivity^74^. Consistent with the well-described action of insulin via AgRP neurons to suppress HGP in mice, this effect on blood glucose was dependent on hypothalamic activity as assessed by functional magnetic resonance imaging. Of note, in separate studies using a lower intranasal insulin dose to avoid insulin spillover into systemic circulation, this led to an increase in hepatic ATP and decreased hepatic triglyceride concentrations in glucose-tolerant controls, but not in patients with type 2 diabetes^75^. Thus, our study points towards a role for insulin action and resistance in tanycytes possibly contributing to multiple manifestations of obesity-associated insulin resistance.

## Methods

### Animal care

All animal procedures were conducted in compliance with protocols approved by local government authorities (Bezirksregierung Köln). Mice were housed in groups of 3–5 at 22–24 °C using a 12 h light/1 h dark cycle in individually ventilated cages. Animals were fed ad libitum chow diet ssniff (V1554, Sniff Spezialdiäten), containing 57% of calories from carbohydrates, 34% calories from protein and 9% calories from fat, or HFD (EF D12492-(I), Sniff Spezialdiäten), containing 21% calories from carbohydrates, 19% calories from protein and 60% calories from fat, starting at the age of 4 weeks until the age of 16–18 weeks, and water and food were only withdrawn if required for an experiment.

### Genetic mouse models

All mouse lines were established on a C57Bl/6 background. We used only males, unless otherwise indicated; littermate mice were age-matched between experimental groups. IR^∆BVEC^ mice were generated by crossing IR^fl/fl^(ref. ^32^) with mice expressing a CreERT2-fusion protein under control of the Slco1c1-promoter (ref. ^33^). Cre-negative IR^fl/fl^ Slco1c1-CreERT2^wt/wt^ littermates were used as controls. Tamoxifen was administered as follows: 1 g of tamoxifen (T5648; Sigma) was suspended in 1 ml of ethanol and dissolved in 9 ml of peanut oil (Sigma). The tamoxifen solution was shaken rigorously at 55 °C and sonicated in an ultrasonic bath sonicator until dissolved. Then, 10 mg per mouse per day was administered per oral gavage (p.o.) to 10-12 week-old male mice via a feeding needle (Fine Science Tools) for 5 consecutive days. Tamoxifen was re-administered after 5 weeks for 2 d. Experimental procedures were performed at least 1 week after the tamoxifen administration.

IR^∆Tan^ mice were induced by injecting 10 to 12-week-old IR^fl/fl^ mice with AAV-Dio2-iCRE-GFP or AAV-Dio2-Cre of mixed serotype 1/2 (ref. ^30^). IR^fl/fl^ littermates that received injection of AAV-Dio2-GFP or AAV-Dio2-mKate2 of mixed serotype 1/2 were used as controls. To test the Cre activity of rAAV vectors, we used ROSA26|STOP|tdTomato^fl/fl^ (ref. ^76^) and ROSA26lSTOPlZsGreen^fl,D/fl,D^ lines^46,77^.

Mice were excluded from analysis if they did not survive during surgical procedures or fibre placement missed the ARC.

For BacTRAP-based ribosomal profiling experiments, ROSA26lSTOPlL10a-GFP ^fl,rox/fl,rox^ mice^46^ were first crossed with Deleter-Dre (CAGGS-Dre) line^46,77,78^ to remove the rox-flanked STOP cassette and generate Cre-dependent ROSA26lSTOPlL10a-GFP^fl,D/fl,D^ (L10a-GFP^+/+^) mice. L10a-GFP^+/+^ mice were crossed with IR^f^^l/fl^ mice to generate IR^wt/wt^ L10a-GFP^+/−^ and IR^fl/fl^ L10a-GFP^+/−^ mice. Twelve-week-old male and female mice were injected with AAV-Dio2-Cre to inactivate IR and induce expression of L10a-GFP specifically in tanycytes (IR^wt/wt^ L10a-EGFP^+/−^, IR^∆Tan^ L10a-GFP^+/−^).

To record in vivo calcium transients in AgRP neurons we employed double recombinase system to specifically inactivate IR in tanycytes and parallelly express GCaMP6 calcium indicator in AgRP neurons. To this end, we generated Agrp-p2a-Dre ^tg/tg^ mice. AgRP-p2A-Dre mice were created using the Efficient additions with ssDNA inserts–CRISPR (Easi-CRISPR) method for generating knock-in mice^79^. To insert the p2a-Dre cassette at the site of the AgRP gene stop codon, a 1,450-base-pair (bp) single-stranded DNA (ssDNA) donor containing a p2A and codon-optimized Dre sequence^78^ flanked by 150-bp left and right homology arms corresponding to the sequence on either side of the stop codon of the AgRP gene was synthesized by Integrated DNA Technologies. A single-guide RNA (sgRNA) designed to cut the genome at the site of the homology arms was synthesized using the Guide-it sgRNA In Vitro Transcription Kit (632635, Takara Bio). The ssDNA, sgRNA and Cas9 protein were injected into one-cell embryos of FVB mice by the BIDMC Transgenic Core. PCR reactions were performed to confirm the sequence of the inserted ssDNA and that the insert was in the correct region of the genome in founder mice. Here, we first crossed IR^fl/fl^ mice with mice to generate IR^fl/fl^ Agrp-p2a-Dre^tg/wt^, which at the age of 10 weeks received either i.c.v. control virus AAV-Dio2-mKate2 or AAV-Dio2-CRE, and AAV-CAG-Frex-GCAMP6 in ARC, thereby generating IR^mKate2^ AgRP^GCaMP6^ and IR^∆Tan^ AgRP^GCaMP6^ control and experimental groups.

### Generation of AAV-Dio2-mKate2 and AAV-Dio2-CRE virus

Tanycyte-specific genetic tools were generated to allow for Cre-mediated modulation of gene expression in tanycytes as well as to generate a control virus with far-red reporter protein (mKate2). Furthermore, to investigate whether modulation of tanycytes interferes with AgRP neuron functionality, we generated a GCaMP6 calcium indicator depending on the Dre recombinase system. AAV-CAG-Frex-GCAMP6 consists of a ubiquitous CAG promoter, and Frex-GCAMP6 an inverted GCaMP6s sequence flanked by two pairs of rox sites on 5ʹ and 3ʹ ends.

AAV-Dio2-mKate2 was generated to replace GFP with a far-red fluorescent protein containing a nuclear localization signal. mKate2 reporter protein sequence was amplified from pAAV-hSynapsin1-GCaMP6s-P2A-mKate2 sequence (Addgene no. 112006) using 5SpemKate2: actagtgccaccatgggtaagaagaagagaagGTGAGCGAG-CTGATTAAGGAGAAC and 3NotmKate2: gcggccgctTATCTGTGCCCCAGTTTGC-AGG primers with the High Fidelity Master PCR (no. 12140314001, Roche) kit.

AAV-Dio2-Cre virus was generated to replace iCRE-GFP with the CRE open-reading frame. AAV-Dio2-iCRE-GFP vector^30^ was digested with NheI and NotI. A Cre-recombinase-containing insert was amplified from TW1 plasmid using 5SpeCre: ACTAGTGCCACCATGGGTAAGAAGAAGAGGAAGGTGTC CAATTT ACTGAC and 3NotCre: GCGGCCGCTAATCGCCATCTTCCAGCAGGC with the High Fidelity Master PCR kit.

Each amplicon (mKate2, Cre) was subcloned into pGem-Teasy via T/A cloning (Promega, A1360), creating two intermediate plasmids: pGEM-Teasy-mKate2, and pGEM-Teasy-Cre. After verifying the correct pGEM-Teasy clones via t7 and S6 primer-based sequencing, correct inserts of interest were released with SpeI and NotI (NEB) from pGEM-Teasy, purified via gel extraction (SmartPure Gel Kit, Eurogentec) and subsequently ligated (NEB) into pAAV-Dio2 vector.

An AAV for Dre recombinase-dependent expression of GCaMP6 was cloned by restriction digesting AAV-CAG-ZsGreen-WPRE with EcoRI and BamHI to remove ZsGreen and generate a backbone. GCaMP6 sequence was custom produced (ThermoFisher Scientific) and digested with EcoRI and BamHI to generate the insert, followed by gel extraction and ligation to finally generate AAV-CAG-Frex-GCAMP6.

### Recombinant AAV production

rAAVs with mosaic capsid of serotypes 1 and 2 (1:1) were generated as previously described^80^ and purified by AVB Sepharose affinity chromatography^81,82^. For each vector, the genomic titre was determined by quantitative PCR using primers against WPRE (WPRE forward primer: 5′-TGCCCGCTGCTGGAC-3′; WPRE reverse primer: 5′-CCGACAACACCACG GAATTG-3′), as described previously^30^. AAV-Dio2-mKate2, AAV-Dio2-Cre and AAV-CAG-Frex-GCAMP6 were produced at Vector Biolabs, USA.

### Stereotactical surgical procedures

Three days before surgery, mice received oral analgesia from tramadol (Tramal, Gruenenthal), provided in the drinking water. Before surgery mice received 0.1 mg kg^−1^ buprenorphine in 0.9% sodium chloride (NaCl) (i.p.) for analgesia. Animals were anesthetized with 4–5% isofluorane and maintained at 1.5–2% throughout the surgical procedure. After loss of reflexes, animals were fixed in a stereotaxic frame (Kopf Instruments). Isoflurane was delivered through a nose cone mounted on the stereotaxic apparatus. Body temperature was maintained throughout the surgery with the use of a heating pad. Eyes were protected by application of eye cream (Bepanthen, Bayer). The head surface was anesthetized using the local anaesthetics lidocaine/prilocaine (Emla Salbe, Aspen Germany) and cleaned with antiseptics (Octenisept, Schülke & Mayr), and a small incision was made to expose the skull. The following coordinates for lateral ventricle relative to Bregma were used: anteroposterior +0.6 mm, mediolateral −0.9 mm, dorsoventral from skull surface −2.8 mm. At defined positions small holes were made using a dental drill. Viral vectors (maximum 3 µl) of AAV1/2-Dio2-iCRE-GFP, AAV1/2-Dio2-GFP, AAV1/2-Dio2-mKate2 or AAV1/2-Dio2-Cre were injected at a rate of 100 nl min^−1^. For fluorescently labelled insulin injections, ghrelin sensitivity test, PET measurement and BacTRAP-based ribosomal profiling of tanycytes experiments, mice received AAV1/2-Dio2-mKate2 or AAV1/2-Dio2-Cre.

For in vivo fibre photometry recordings, AAV1/2-Dio2-CRE or AAV-Dio2-mKate2 was i.c.v. injected and 2 × 500 nl of AAV-CAG-Frex-GCAMP6 (1.1 × 10^13^ genomic particles per ml) was injected into ARC relative to Bregma: Anterior-Posterior (AP): −1.4 mm, Medial-Lateral (ML): + 0.20 mm, Dorsal-Ventral (DV): −5.85 mm and at −5.70 mm. For recordings, a commercially available photometry canula (400 µm, MFC 400/430-0.66 6 mm SM3(P)_FLT, no. B280-4681-6, Doric Lenses) was implanted unilaterally over the ARC: AP: −0.6 mm, ML: +0.25 mm, DV: −5.85, with DV angle of 8°.

After injection the scalp was sutured. Post operation animals received analgesia with meloxicam 5 mg kg^−1^ and tramadol provided in the drinking water (twice a day 1 mg ml^−1^) for 3 consecutive days. Body weights were continuously monitored during recovery.

### Insulin tolerance test and glucose tolerance test

Insulin and glucose tolerance tests were performed in random-fed or overnight-fasted animals, respectively, as previously described^5,46^.

### Indirect calorimetry

Indirect calorimetry was performed using an open-circuit, indirect calorimetry system (PhenoMaster, TSE Systems) as previously described^83^. Mice 14–17 weeks old were acclimatized in training cages for 3 d before data acquisition to adapt to food and water dispensers of the system.

### Marble burying test

A marble burying test was used to assess obsessive-compulsive behaviour. Eighteen marbles were evenly distributed on bedding. The behaviour of each mouse was monitored by an observer blinded to the genotypes, and the number of marbles, of which the surface was covered by 3/4 in bedding material at each time point (every 5 min for 30 min) was registered.

### Open-field test

An open-field test was used to assess locomotor behaviour. Experiments were performed in polycarbonate boxes of 50 × 50 × 30 cm^3^ (length, width, height) (TSE Systems), which were illuminated with white/red light from above. Behaviour was recorded over a period of 5 min during light cycle using an automated camera- and video-based system, VideoMot 2 (TSE Systems).

### Hyperinsulinaemic–euglycaemic clamp studies in awake mice

Implantation of catheters into the jugular vein and the clamp procedure employed were performed in 16-week-old mice as described before^5^. After 5–6 d of recovery, mice that had lost less than 15% of their preoperative weight were subjected to the clamp. On the day of experimentation, each animal was deprived of food for 4 h in the morning. All solutions infused were prepared with 3% plasma added, obtained from donor mice of the same genetic background that had been fasted for 4 h.

A primed-continuous infusion of tracer d-[3-3H]-glucose (Perkin Elmer) was initiated 90 min before the clamping (0.8 μCi) and then infused continuously at a rate of 0.04 µCi min^−1^.

During the clamp period, insulin (Insunam Rapid, Sanofi) was infused at a fixed rate of 4 µU g^−1^ min^−1^ together with clamp solution 0.04 µCi µl^−1^ in 40% glucose (Delta Select). Blood glucose concentrations were monitored regularly according to a fixed scheme from tail vein bleedings (Hemocue Glucose 201 RT), and maintained around 120–140 mg dl^−1^ by adjusting the clamp solution. Steady state was considered achieved when a fixed glucose-infusion rate kept the glucose concentration in blood constant for 30 min. During the steady state, blood samples were collected for the measurement of steady-state parameters. When the steady state was reached after 120 min, an infusate of 2-deoxy-d-[1-14 C]-glucose (10 μCi; American Radiolabeled Chemicals) was given for tissue-specific glucose uptake. Clamps were continued by initially giving a d-[3-3H]-glucose bolus of 1.6 µCi in 40% glucose and then switching to the previous GIR and insulin rate of 4 µU g^−1^ min^−1^. At the end of the experiment, mice were killed by decapitation and gonadal WAT, BAT and skeletal muscle were dissected and stored at −80 °C until further analysis.

The [3-3H]-glucose content in serum during basal conditions and at steady state was measured as described earlier^5^. Tissue uptake rates of 2-deoxy-d-[1-14 C]-glucose were assessed as previously described^5^.

### Food intake measurements

At least 14 d before food intake measurements, mice were acclimatized to food hoppers in their home cage. Ad libitum food intake was measured for 3 constitutive days in the morning just after the light cycle started at 7:00–7:30 and just before the dark cycle started at 18:30–19:00. Food intake for re-feeding experiments was measured at the indicated time points after overnight fast.

### Insulin stimulation in vena cava

Mice 17 weeks (IR^∆BVEC^ mice) to 19 weeks (IR^∆Tan^ mice) old were fasted overnight for 16 h and anesthetized with ketamine/xylazine (100 mg kg^−1^/22 mg kg^−1^). Insulin at 0.5 IU kg^−1^ (Insunam Rapid, Sanofi) was injected in the vena cava and animals were perfused at 5, 10, 20 at 30 min post injection as described below. Mice for time point 0 min did not receive an injection, but were perfused after being deeply anesthetized. Brains were further processed for pAKT immunohistochemistry as described below.

### Fluorescently labelled insulin injection and imaging

The 17-week-old mice were fasted overnight for 16 h and anesthetized with ketamine/xylazine (100 mg kg^−1^/22 mg kg^−1^). Fluorescently labelled insulin (F488-labelled insulin) (250 nmol kg^−1^) (Novo Nordisk) was injected in the vena cava and the animals were perfused after 15 min as described below. To retain spontaneous fluorescence signal and avoid overfixation, the paraformaldehyde (PFA) was infused for 1 min (~10 ml). After postfixation, mouse brains were further cut on a cryostat and some sections were directly collected on a SuperFrost (ThermoFisher Scientific) and mounted with Vectashield (Vectorlabs) for imaging, whereas the rest of the sections were collected in anti-freeze solution (30% ethylene glycol and 20% glycerol in PBS) and used for immunohistochemistry with Alexa-Fluor-488 (no. A11094, ThermoFisher Scientific) and pAKT as described below.

### Leptin sensitivity test

Sixteen-week-old mice were acclimatized to food hoppers in their home cage for 2 weeks before the leptin sensitivity test. At 2 d before the first experimental day, mice were picked daily to acclimatize to short fixation and mimic injection. Mice received i.p. 0.9% NaCl twice daily at 7:00–8:00 and 18:00–19:00 for 3 constitutive days and then i.p. 2 mg kg^−1^ leptin (no. 450-31, Peprotech) for 3 d twice daily. Before each injection body weight and food intake were measured.

### Ghrelin sensitivity test

Eighteen-week-old random-fed animals in a cross-over study design were used for ghrelin sensitivity tests. At 9:00 the animals were placed in a fresh home cage and food was removed for 1 h before the injection. Mice, which were randomly assigned to the groups, received either i.p. ghrelin (1 mg kg^−1^) (031-31, Peprotech) or 0.9% NaCl. The food intake was measured at 1 h, 2 h and 4 h. One week later a cross-over experiment was performed.

### Leptin stimulation

The 17–18-week-old animals were fasted overnight for 16 h. The next morning, mice received i.p. 3 mg kg^−1^ leptin (Peprotech) and 20 min later were decapitated. Brains were postfixed only for 2 h in 2% PFA and placed in 20% sucrose at 4 °C until the brain sank. Mouse brains were further processed for pSTAT3 immunohistochemistry as described below.

### In vivo fibre photometry studies

#### Photometry set-up

Fibre photometry was performed using an RZ5P real-time processor (Tucker-Davis Technologies). RZ5P outputs were connected to an LED Driver (Doric Lenses) for external modulation of the light sources. Light from connected 405-nm (isosbestic control) and 465-nm (GCaMP6) light-emitting diodes (P/N CLED_405, P/N CLED_465) was passed through a four-port fluorescence minicube (FMC_AE(405)_E1(460-490)_F1(500-550)_S, Doric Lenses) and collected with a photoreceiver (Model 2151, New Focus).

A fibre optic cable was attached to the implanted fibre optic canula. IR^mKate2^ AgRP^GCaMP6^ and IR^∆Tan^ AgRP^GCaMP6^ mouse groups were acclimatized to the set-up 3 weeks post surgery and 1 week before recordings. For the measurement, mice were placed in a regular type 2 cage at room temperature. Water and food were removed during the recordings and introduced only if the experimental settings allowed. The location of the fibre tip was identified post hoc using histology. Mice with missed injections or wrong fibre placement were excluded from the analysis.

#### Hormone injections and food presentation during in vivo fibre photometry

One week before the experiments, mice were habituated to the room and experimental set-up. Before recordings mice were habituated for 5 min, then a 10-min baseline was recorded, followed by hormone injections or food presentation and further recording for 20 min. All experiments were performed at 8:00–12:30. CCK, 5-HT and PBS control were injected and food was presented after a 16-h overnight fast, whereas ghrelin was administered in nonfasted animals. The following doses were used: ghrelin, 60 µg per mouse (1465, Tocris); CCK octapeptide, 10 µg kg^−1^ (4033010.0001, Bachem); 5-hydroxytryptamine hydrochloride (5-HT), 2 mg kg^−1^ (H9523, Sigma Aldrich)^47^. All compounds were diluted in NaCl.

#### Photometry analysis

Changes in the calcium-dependent GCaMP6 fluorescence (465 nm) were compared with a 405-nm isosbestic control, to provide internal control for movement and auto bleaching artefacts. Fluorescence measurements were recorded employing Synapse software (v.95-43718P, Tucker-Davis Technologies) and analysed using a custom MATLAB script. The fluorescence signal was defined as ratio of fluorescence at 465 nm to the fluorescence measured at 405 nm. For i.p. hormone injection and food exposure experiments, the median of the baseline recording before treatment was defined as *F*_0_. The post-treatment signal (d*F*/*F*_0_) was calculated by comparing the fluorescence signal with the pretest baseline (d*F*(*t*)/*F*_0_ = (*F*(*t*) − *F*_0_)/*F*_0_). For all experiments photobleaching correction was not necessary, due to the low laser power used and optimized patchcords, which prevent photobleaching of the optical system. In the figures, d*F*/*F*(%) represents the mean d*F*(*t*)/*F*_0_ × 100.

### Perfusion fixation

Anesthetized mice were perfused transcardially with ice-cold PBS followed by ice-cold 4% PFA dissolved in PBS (pH 7.4). Brains were postfixed in 4% PFA overnight at 4 °C, if not stated otherwise, and then moved to 20% sucrose in PBS at 4 °C until the brain sank. Brains were cut in a cryostat 20-µm thick for RNA in situ hybridization or 30-µm thick for pAKT immunohistochemistry. Cut brain slices were collected in anti-freeze solution (30% ethylene glycol and 20% glycerol in PBS) and stored at −20 °C.

### RNA in situ hybridization

All reagents and materials were purchased from Advanced Cell Diagnostics, if not otherwise stated. *Agrp* (no. 40071-C2), *POMC* (no. 31408-C3), *Fos* (no. 316921), *Insr* (no. 401011), *Dio2* (no. 479331), *GFAP* (313211-C2) and *ZsGreen* (no. 461251-C3) mRNA was detected using a FISH technique (RNAscope) following the manufacturer’s protocol (Advanced Cell Diagnostics). As a control, 3-plex (no. 320881) or 2-plex (no. 321651) positive and 3-plex (no. 320871) or 2-plex (no. 320751) negative probes were run in parallel with the target probes. At the first day of the assay, sections were mounted on SuperFrost Plus Gold slides (ThermoFisher Scientific) and air-dried for at least 2 h at 60 °C. Briefly, sections were exposed to H_2_O_2_ (no. 322330) for 10 min at room temperature, submerged in Target Retrieval at ~99 °C for 15 min, then shortly rinsed in distilled water, dehydrated in 100% ethanol for 30 s and finally air-dried for 5 min. After creating a hydrophobic barrier, the slides were incubated in Protease Plus for 15 min. Subsequent hybridization and amplification steps were carried out using RNAscope Fluorescent Multiplex Detection Reagent kit (no. 320851) according to the manufacturer’s protocol. During the hybridization, *Fos* and *Insr* probes were diluted 1:2, and *Agrp* and *POMC* probes were diluted 1:200. Probes were detected using 1:1,000 diluted Opal fluorophores from Perkin Elmer, where *Fos* and *Dio2* were detected with Opal 690 (no. FP1497001KT); *POMC* with Opal 620 (no. FP1495001KT); *Agrp*, *Insr* and *GFAP* with Opal 570 (no. FP1488001KT); and *ZsGreen* with Opal 520 (no. FP1487001KT).

### Immunohistochemistry

The immunohistochemistry assays of pAKT and pSTAT3 were performed using the TSA Plus Cyanine 3 and Fluorescein System (no. NEL753001KT, Perkin Elmer) according to the manufacturer’s recommendations.

pSTAT3 immunohistochemical staining was performed on brain slices of leptin (3 mg kg^−1^) (Peprotech)-stimulated (20 min) mice. The 20-µm thick floating sections were mounted on glass slides (SuperFrost, ThermoFisher Scientific) and fixed for 5 min with 2% PFA, treated with 0.3% H_2_O_2_ for 15 min and incubated for 5 min in ice-cold methanol. Between each step slides were washed twice for 10 min with PBS. Sections were incubated for 1 h in TSA blocking solution (Perkin Elmer), directly followed with incubation for 2 d at 4 °C in primary antibody pSTAT3 (1:100, no. 9145, Cell Signalling) diluted in TSA blocking solution. In between the upcoming steps, slides were washed three times for 10 min in 0.3% TritonX in PBS. Next, slides were incubated in HRP (1:100, no. NEF812001EA, Perkin Elmer) and DAPI (1:1,000, no. 62248, ThermoFisher Scientific) diluted in 0.25% TritonX in PBS for 30 min at room temperature. Immunocomplexes were revealed with Cy3 fluorophore incubated for 10 min in amplification buffer (1:100, Perkin Elmer).

pAKT immunohistochemical staining was performed on brain slices of insulin (0.5 IU kg^−1^)- and 488-insulin (250 nmol kg^−1^)-stimulated mice. All immunohistochemistry steps were performed on free floating sections. In between steps sections were washed in 0.05% Tween20 in Tris-buffered saline. Briefly, brain slices were rinsed in Tris-buffered saline for 5 min, washed and then incubated in 1% H_2_O_2_ for 30 min. Sections were blocked with TNB buffer, consisting of 0.1 M Tris-HCl, pH 7.5, 0.15 M NaCl and 0.5% (w/v) Blocking Reagent (no. FP1020, Perkin Elmer). pAKT primary antibody (1:500, no. 4060, Cell Signalling) diluted in TNB buffer was incubated overnight at room temperature. HRP-labelled anti-rabbit-IgG (no. NEF812E001EA, Perkin Elmer) antibody and DAPI (1:1,000, no. 62248, ThermoFisher Scientific) were incubated for 30 min at room temperature. Immunocomplexes were revealed with Cy3 fluorophore by incubation for 3 min in amplification buffer (1:100, Perkin Elmer). Brain slices were then mounted on glass slides (SuperFrost, ThermoFisher Scientific) with Vectashield (Vectorlabs).

The signals of fluorescently labelled insulin (488-insulin) and mKate2 fluorophore and endogenous GFP were amplified via immunohistochemistry staining against the fluorescent tag Alexa-Fluor-488 (1:500, no. A11094, ThermoFisher Scientific), red fluorescent protein (1:100, no. R10367, ThermoFisher Scientific) and GFP (1:1,000, no. 13970, Abcam), respectively. All immunohistochemistry steps were performed on free floating sections. In between steps sections were washed in PBS. Briefly, brain slices were rinsed in PBS twice for 10 min, washed and then incubated in 0.3% glycine for 10 min, followed by 0.03% SDS for 10 min. Sections were blocked in 3% donkey serum for 60 min at room temperature. The primary antibody was incubated overnight at room temperature and afterwards washed three times for 10 min. Immunocomplexes were revealed with Alexa-594 (1:500, A21207, ThermoFisher Scientific) by incubation for 60 min. Brain slices were then mounted on glass slides (SuperFrost, ThermoFisher Scientific) with Vectashield (Vectorlabs).

### Microscopy and quantification

Images were captured using a Leica TCS confocal microscope and LasX software, equipped with ×20/0.75 immersion liquid and ×40/1.30 oil objectives. Images of ARC were captured at median eminence area, rendering 3–5 sections per animal. Images were imported into Fiji (v.2.0.0-rc-69/1.52n, NIH) for manual quantification of pAKT- and pSTAT3-positive cells per side of the hypothalamus. pAKT signal was measured as mean intensity in basal tanycytes and lateral tanycytes (average of lateral tanycytes of right and left hemisphere). pAKT in lectin-positive vessels was quantified as described before^84^. *Fos*-positive AGRP and POMC neurons were manually quantified using Fiji software from one side of ARC from 3–5 sections per animal.

### PET imaging

PET imaging was performed in a cross-over study using an Inveon Preclinical PET/CT system (Siemens) as previously described^84^. Mice were anaesthetized with 2% isoflurane in 65%/35% nitrous oxide/oxygen gas and positioned on a dedicated mouse carrier (MEDRES, Germany), carrying two mice. For injection of the radiotracer, a catheter consisting of a 30-G cannula connected to polythene tubing (internal diameter = 0.28 mm) was inserted into the tail vein and fixated by a drop of glue. Mice were injected either with i.p. 0.325 IU kg^−1^ insulin (Insunam Rapid, Sanofi) or with saline, and 1 week later the groups were switched in a cross-over design. After starting the PET scan, 7–8 MBq of [18 F]FDG in 50−100 µl saline was injected per mouse. Emission data were acquired for 45 min. Thereafter, animals were automatically moved into the computer tomography (CT) gantry and a CT scan was performed (180 projections per 360°, 200 ms, 80 kV, 500 µA). The CT data were used for attenuation correction of the PET data and the CT image of the skull was used for image coregistration. PET data were histogrammed in time frames of 12 × 30 s, 3 × 60 s, 3 × 120 s and 7 × 240 s, Fourier rebinned, and images were reconstructed using the maximum a posteriori shifted Poisson (MAP-SP) algorithm provided by the manufacturer. For coregistration the imaging analysis software Vinci was used^85^. Images were coregistered to a three-dimensional (3D) mouse brain atlas constructed from the two-dimensional mouse brain atlas published by Paxinos^86^.

#### Kinetic modelling

An image-derived input function was extracted from the PET data of the aorta, which could be identified in the image of the first time frame of each animal. Input function data were corrected for partial volume effect by assuming a standardized volume fraction of 0.6 (ref. ^87^). Parametric images of the [18 F]FDG kinetic constants *k*1, *k*2, *k*3 and *k*4 were determined by a voxel-by-voxel (voxel size = 0.4 × 0.4 × 0.8 mm^3^) fitting of data to a two-tissue-compartment kinetic model. The ratio of tissue and plasma glucose concentrations (*C*_e_/*C*_p_) is a measure for glucose transport and is given by *C*_e_/*C*_p_ = *k*1/(*k*2 + *k*3/0.26) (refs. ^84,88^). Since neuronal activation is accompanied by increased glucose transport and this parameter is less sensitive to changes in plasma glucose level, we use alterations of glucose transport (*C*_e_/*C*_p_) as a surrogate for alterations in neuronal activation.

#### Statistics

Statistical testing was performed by application of a voxel-wise *t*-test between groups. 3D maps of *P* values allow for identification of regions with significant differences in the parameters. From these regions we defined volumes of interest and performed additional statistical testing for these volumes of interest. For presentation only, 3D maps of *P* values were re-calculated on a 0.1 × 0.1 × 0.1-mm^3^ grid from the original dataset using trilinear interpolation.

### BacTRAP-based ribosomal profiling of tanycytes

Affinity purifications of translating ribosomes were performed as published before^46,89–92^. Twelve-week-old NCD- and HFD-fed IR^wt/wt^ L10a-EGFP^+/−^ mice and NCD-fed IR^fl/fl^ L10a-GFP^+/−^ mice were injected in the lateral ventricle with AAV-Dio2-Cre virus and killed 4 weeks post virus injection. Hypothalami were quickly extracted using an ice-cold stainless brain matrix (World Precision Instruments). Arcuate and median eminence regions were collected, frozen on dry ice and stored at −80 °C. Four to seven hypothalami were pooled per replicate. Further steps of the immunopurification of L10a-GFP-tagged ribosomes and subsequent extraction of RNA were performed exactly as previously described^91^. RNA was eluted in 10 µl of nuclease-free water. RNA integrity was assessed using an Agilent 2100 bioanalyzer and RNA concentration was measured using a Qubit fluorometer (ThermoFisher Scientific).

#### RNA sequencing

RNA sequencing of mRNA immunoprecipitated from translating ribosomes was performed at Cologne Centre for Genomics as previously described^91,93,94^. Pre-amplification using the Ovation RNASeq System V2 was performed as previously described. Total RNA was used for first-strand complementary DNA synthesis, using both poly(T) and random primers, followed by second-strand synthesis and isothermal strand-displacement amplification. For library preparation, the Illumina Nextera XT DNA sample preparation protocol was used, with 1 ng of cDNA input. After validation (Agilent 2200 TapeStation) and quantification (Invitrogen Qubit System), transcriptome libraries were pooled. The pool was quantified using the Peqlab KAPA Library Quantification Kit and Applied Biosystems 7900HT Sequence Detection and sequenced on an Illumina NovaSeq sequencing instrument with a 2 × 100-bp paired-end read length.

#### Analysis of RNA-sequencing data

We applied the community-curated nfcore^95,96^ rnaseq analysis pipeline (v.1.4). The gene-level quantification was carried out using Salmon^97^ (v.0.14.1) using the reference genome GRCm38. The differential gene expression analysis was done using the DESeq2 (ref. ^98^) (v.1.30.0) R package, R studio v.1.1.463.

We filtered for protein-coding genes using the Ensembl^99,100^ biomaRt package. Furthermore, we excluded lowly abundant genes by requiring a minimum transcripts per million count of 1. We determine tanycyte-related genes by extracting differentially upregulated genes between tanycyte ribosomal pulldown (immunoprecipitation) and the hypothalamic background (input) per sample (adjusted *P* ≤ 0.05, log_2_(fold change) > 0). DEGs between the tanycyte immunoprecipitation samples are calculated for conditions of NCD control/IR^ΔTan^ and NCD control/HFD control (adjusted *P* ≤ 0.05). We then restricted these DEGs to our tanycyte-related gene list derived from the immunoprecipitation versus input comparisons to ensure we are focusing on effects happening in tanycytes, and visualized it employing R studio, v.3.5.3. (https://www.R-project.org/). The regulated GO terms were derived using the clusterProfiler R package from tanycyte-related genes and are visualized in a table.

We incorporated ggsashimi^96^ for visualizing splicing events and read coverage of the IR gene *Insr* (Chromosome 8: 3,172,061–3,329,617) by displaying exon-spanning reads of our IR^ΔTan^ versus NCD control bacTRAP tanycyte pulldowns. For visual clarity, we require a minimum of ten exon-spanning reads to be displayed.

### Statistical analysis

Numbers of replicates (*n*) and performed statistical tests are indicated in the figure legends. For quantitative analyses, littermates were used. For RNA-sequencing experiments, *n* represents the number of biological replicates (details on the number of mice pooled for each replicate can be found in the BacTRAP-based ribosomal profiling of tanycytes section). For Hyperinsulinaemic–euglycaemic clamp studies in awake mice experiments, only mice for which sufficient blood for analysis could not be collected during the clamp procedure were excluded from analysis.

Data presentation with scatter dot plot graphs includes values of individual data points, with mean values as a bar ± s.e.m. error bars. Data are presented in violin plots with upper and lower quartiles and mean. Error bars in the line graphs show s.e.m. For pairwise comparisons of dependent and independent normal distributions, paired and unpaired *t*-tests were used, respectively. One-way analysis of variance (ANOVA) with Tukey post hoc test was used for pairwise comparisons of more than two groups. Two-way ANOVA with Šídák post hoc test was used to compare genotypes and treatments over time points.

Tests were executed using GraphPad Prism 8 (GraphPad Software). For all statistical tests, significance was measured against an alpha value of 0.05 unless otherwise stated. *P < 0.05, **P < 0.01, ***P < 0.001, ****P<0.0001.

### Reporting Summary

Further information on research design is available in the Nature Research Reporting Summary linked to this article.

## Supplementary information

Reporting Summary

## Data Availability

Raw RNA-seq data have been deposited in the NCBI Gene Expression Omnibus under accession code: GSE185074. Other raw data are available under reasonable request to the corresponding author. Source data are provided with this paper.

## Source data

Source Data Fig. 1 Statistical source data.
Source Data Images Fig. 1 Source data images.
Source Data Fig. 2 Statistical source data.
Source Data Images Fig. 2 Source data images.
Source Data Fig. 3 Statistical source data.
Source Data Fig. 4 Statistical source data.
Source Data Fig. 5 Statistical source data.
Source Data Fig. 6 Statistical source data.
Source Data Fig. 7 Statistical source data.
Source Data Fig. 8 Statistical source data.
Source Data Images Fig. 8 Source data images.
Source Data Extended Data Fig. 2 Statistical source data.
Source Data Extended Data Fig. 3 Unprocessed gels.
Source Data Extended Data Fig. 4 Statistical source data.
Source Data Extended Data Fig. 5 Statistical source data.
Source Data Extended Data Fig. 6 Statistical source data.
Source Data Images Extended Data Fig. 6 Source data images.
Source Data Extended Data Fig. 7 Statistical source data.
Source Data Extended Data Fig. 8 Statistical source data.
Source Data Images Extended Data Fig. 8 Source data images.

## Code availability

The code for RNA-seq analysis is available at https://github.com/bruening-lab/ insulin-receptors-in-tanycytes-gate- hypothalamic-insulin-uptake-and- regulate-agrp-neuronal-activity.

## References

[1] Könner AC, Brüning JC (2012). Selective insulin and leptin resistance in metabolic disorders. Cell Metab..

[2] Brüning JC (2000). Role of brain insulin receptor in control of body weight and reproduction. Science.

[3] Koch L (2008). Central insulin action regulates peripheral glucose and fat metabolism in mice. J. Clin. Invest..

[4] Scherer T (2011). Brain insulin controls adipose tissue lipolysis and lipogenesis. Cell Metab..

[5] Könner AC (2007). Insulin action in AgRP-expressing neurons is required for suppression of hepatic glucose production. Cell Metab..

[6] Klöckener T (2011). High-fat feeding promotes obesity via insulin receptor/PI3K-dependent inhibition of SF-1 VMH neurons. Nat. Neurosci..

[7] Hausen AC (2016). Insulin-dependent activation of MCH neurons impairs locomotor activity and insulin sensitivity in obesity. Cell Rep..

[8] Steculorum SM (2016). AgRP neurons control systemic insulin sensitivity via myostatin expression in brown adipose tissue. Cell.

[9] Ruud J, Steculorum SM, Brüning JC (2017). Neuronal control of peripheral insulin sensitivity and glucose metabolism. Nat. Commun..

[10] Dodd GT (2017). A hypothalamic phosphatase switch coordinates energy expenditure with feeding. Cell Metab..

[11] Dodd GT (2021). Insulin signaling in AgRP neurons regulates meal size to limit glucose excursions and insulin resistance. Sci. Adv..

[12] Banks WA, Jaspan JB, Huang W, Kastin AJ (1997). Transport of insulin across the blood-brain barrier: saturability at euglycemic doses of insulin. Peptides.

[13] Schwartz MW (1990). Insulin binding to brain capillaries is reduced in genetically obese, hyperinsulinemic Zucker rats. Peptides.

[14] Banks WA, Kastin AJ (1998). Differential permeability of the blood–brain barrier to two pancreatic peptides: insulin and amylin. Peptides.

[15] Hersom M (2018). The insulin receptor is expressed and functional in cultured blood-brain barrier endothelial cells but does not mediate insulin entry from blood to brain. Am. J. Physiol. Endocrinol. Metab..

[16] Rhea EM, Rask‐Madsen C, Banks WA (2018). Insulin transport across the blood–brain barrier can occur independently of the insulin receptor. J. Physiol..

[17] Konishi M (2017). Endothelial insulin receptors differentially control insulin signaling kinetics in peripheral tissues and brain of mice. Proc. Natl Acad. Sci. USA.

[18] Prevot V (2018). The versatile tanycyte: a hypothalamic integrator of reproduction and energy metabolism. Endocr. Rev..

[19] García-Cáceres C (2019). Role of astrocytes, microglia, and tanycytes in brain control of systemic metabolism. Nat. Neurosci..

[20] Mullier A, Bouret SG, Prevot V, Dehouck B (2010). Differential distribution of tight junction proteins suggests a role for tanycytes in blood-hypothalamus barrier regulation in the adult mouse brain. J. Comp. Neurol..

[21] Banks WA, Kastin AJ, Pan W (1999). Uptake and degradation of blood-borne insulin by the olfactory bulb. Peptides.

[22] Balland E (2014). Hypothalamic tanycytes are an ERK-gated conduit for leptin into the brain. Cell Metab..

[23] Schaeffer M (2013). Rapid sensing of circulating ghrelin by hypothalamic appetite-modifying neurons. Proc. Natl Acad. Sci. USA.

[24] Uriarte M (2019). Evidence supporting a role for the blood-cerebrospinal fluid barrier transporting circulating ghrelin into the brain. Mol. Neurobiol..

[25] Duquenne, M. et al. Leptin brain entry via a tanycytic LepR-EGFR shuttle controls lipid metabolism and pancreas function. *Nat. Metab*. 10.1038/s42255-021-00432-5 (2021). 10.1038/s42255-021-00432-5 PMC7611554 34341568

[26] Parkash J (2015). Semaphorin7A regulates neuroglial plasticity in the adult hypothalamic median eminence. Nat. Commun..

[27] García MdelosA (2003). Hypothalamic ependymal-glial cells express the glucose transporter GLUT2, a protein involved in glucose sensing: GLUT2 expression in hypothalamic glial tanycytes. J. Neurochem..

[28] Lazutkaite G, Soldà A, Lossow K, Meyerhof W, Dale N (2017). Amino acid sensing in hypothalamic tanycytes via umami taste receptors. Mol. Metab..

[29] Geller S (2019). Tanycytes regulate lipid homeostasis by sensing free fatty acids and signaling to key hypothalamic neuronal populations via FGF21 secretion. Cell Metab..

[30] Müller-Fielitz H (2017). Tanycytes control the hormonal output of the hypothalamic–pituitary–thyroid axis. Nat. Commun..

[31] Yoo S (2020). Tanycyte ablation in the arcuate nucleus and median eminence increases obesity susceptibility by increasing body fat content in male mice. Glia.

[32] Brüning JC (1998). A muscle-specific insulin receptor knockout exhibits features of the metabolic syndrome of NIDDM without altering glucose tolerance. Mol. Cell.

[33] Ridder DA (2011). TAK1 in brain endothelial cells mediates fever and lethargy. J. Exp. Med..

[34] Gropp E (2005). Agouti-related peptide–expressing neurons are mandatory for feeding. Nat. Neurosci..

[35] Lin HV (2010). Divergent regulation of energy expenditure and hepatic glucose production by insulin receptor in agouti-related protein and POMC neurons. Diabetes.

[36] Krashes MJ (2011). Rapid, reversible activation of AgRP neurons drives feeding behavior in mice. J. Clin. Invest..

[37] Dietrich MO, Zimmer MR, Bober J, Horvath TL (2015). Hypothalamic Agrp neurons drive stereotypic behaviors beyond feeding. Cell.

[38] Chen Y (2019). Sustained NPY signaling enables AgRP neurons to drive feeding. eLife.

[39] Nakazato M (2001). A role for ghrelin in the central regulation of feeding. Nature.

[40] Cowley MA (2003). The distribution and mechanism of action of ghrelin in the CNS demonstrates a novel hypothalamic circuit regulating energy homeostasis. Neuron.

[41] Seoane LM (2003). Agouti-related peptide, neuropeptide Y, and somatostatin-producing neurons are targets for ghrelin actions in the rat hypothalamus. Endocrinology.

[42] Fuente-Martín E (2016). Ghrelin regulates glucose and glutamate transporters in hypothalamic astrocytes. Sci. Rep..

[43] Rhea EM (2018). Ghrelin transport across the blood–brain barrier can occur independently of the growth hormone secretagogue receptor. Mol. Metab..

[44] Briggs DI, Enriori PJ, Lemus MB, Cowley MA, Andrews ZB (2010). Diet-induced obesity causes ghrelin resistance in arcuate NPY/AgRP neurons. Endocrinology.

[45] Briggs DI (2014). Evidence that diet-induced hyperleptinemia, but not hypothalamic gliosis, causes ghrelin resistance in NPY/AgRP neurons of male mice. Endocrinology.

[46] Biglari, N. et al. Functionally distinct POMC-expressing neuron subpopulations in hypothalamus revealed by intersectional targeting. *Nat. Neurosci*. 10.1038/s41593-021-00854-0 (2021). 10.1038/s41593-021-00854-0 PMC8249241 34002087

[47] Beutler LR (2017). Dynamics of gut-brain communication underlying hunger. Neuron.

[48] Chen Y, Lin Y-C, Kuo T-W, Knight ZA (2015). Sensory detection of food rapidly modulates arcuate feeding circuits. Cell.

[49] Beutler LR (2020). Obesity causes selective and long-lasting desensitization of AgRP neurons to dietary fat. eLife.

[50] Hachiya, H. L., Halban, P. A. & King, G. L. Intracellular pathways of insulin transport across vascular endothelial cells. *Am. J. Physiol. Cell Physiol*. 10.1152/ajpcell.1988.255.4.C459 (1988). 10.1152/ajpcell.1988.255.4.C459 3052101

[51] King GL, Johnson SM (1985). Receptor-mediated transport of insulin across endothelial cells. Science.

[52] Banks WA, Owen JB, Erickson MA (2012). Insulin in the brain: there and back again. Pharmacol. Ther..

[53] Duffy KR, Pardridge WM (1987). Blood-brain barrier transcytosis of insulin in developing rabbits. Brain Res..

[54] Frank HJL, Jankovic-Vokes T, Pardridge WM, Morris WL (1985). Enhanced insulin binding to blood-brain barrier in vivo and to brain microvessels in vitro in newborn rabbits. Diabetes.

[55] Kondo T, Hafezi-Moghadam A, Thomas K, Wagner DD, Kahn CR (2004). Mice lacking insulin or insulin-like growth factor 1 receptors in vascular endothelial cells maintain normal blood-brain barrier. Biochem. Biophys. Res. Commun..

[56] Porte D, Baskin DG, Schwartz MW (2005). Insulin signaling in the central nervous system: a critical role in metabolic homeostasis and disease from *C. elegans* to humans. Diabetes.

[57] Langlet F (2014). Tanycytes: a gateway to the metabolic hypothalamus. J. Neuroendocrinol..

[58] Langlet F (2013). Tanycytic VEGF-A boosts blood-hypothalamus barrier plasticity and access of metabolic signals to the arcuate nucleus in response to fasting. Cell Metab..

[59] Peruzzo B, Pastor FE, Blázquez JL, Amat P, Rodríguez EM (2004). Polarized endocytosis and transcytosis in the hypothalamic tanycytes of the rat. Cell Tissue Res..

[60] Yoo S, Cha D, Kim DW, Hoang TV, Blackshaw S (2019). Tanycyte-independent control of hypothalamic leptin signaling. Front. Neurosci..

[61] Scarlett JM (2016). Central injection of fibroblast growth factor 1 induces sustained remission of diabetic hyperglycemia in rodents. Nat. Med..

[62] Bentsen MA (2020). Transcriptomic analysis links diverse hypothalamic cell types to fibroblast growth factor 1-induced sustained diabetes remission. Nat. Commun..

[63] Garfield AS (2015). A neural basis for melanocortin-4 receptor–regulated appetite. Nat. Neurosci..

[64] Könner AC, Hess S, Tovar S, Mesaros A, Sánchez-Lasheras C, Evers N, Verhagen LA, Brönneke HS, Kleinridders A, Hampel B, Kloppenburg P, Brüning JC (2011). Role for insulin signaling in catecholaminergic neurons in control of energy homeostasis. Cell Metab..

[65] Figlewicz DP, Evans SB, Murphy J, Hoen M (2003). Expression of receptors for insulin and leptin in the ventral tegmental area/substantia nigra (VTA/SN) of the rat. Brain Res..

[66] Zhang X, van den Pol AN (2017). Rapid binge-like eating and body weight gain driven by zona incerta GABA neuron activation. Science.

[67] Pasquettaz R (2021). Peculiar protrusions along tanycyte processes face diverse neural and nonneural cell types in the hypothalamic parenchyma. J. Comp. Neurol..

[68] Roden M, Shulman GI (2019). The integrative biology of type 2 diabetes. Nature.

[69] Hotamisligil GS (2006). Inflammation and metabolic disorders. Nature.

[70] Mazzone CM (2020). High-fat food biases hypothalamic and mesolimbic expression of consummatory drives. Nat. Neurosci..

[71] Wallum BJ (1987). Cerebrospinal fluid insulin levels increase during intravenous insulin infusions in man. J. Clin. Endocrinol. Metab..

[72] Kern W (2006). Low cerebrospinal fluid insulin levels in obese humans. Diabetologia.

[73] Kullmann S, Heni M, Fritsche A, Preissl H (2015). Insulin action in the human brain: evidence from neuroimaging studies. J. Neuroendocrinol..

[74] Heni M (2014). Central insulin administration improves whole-body insulin sensitivity via hypothalamus and parasympathetic outputs in men. Diabetes.

[75] Gancheva S (2015). Effects of intranasal insulin on hepatic fat accumulation and energy metabolism in humans. Diabetes.

[76] Madisen L (2010). A robust and high-throughput Cre reporting and characterization system for the whole mouse brain. Nat. Neurosci..

[77] Löhr H (2018). Diet-induced growth is regulated via acquired leptin resistance and engages a Pomc-somatostatin-growth hormone circuit. Cell Rep..

[78] Anastassiadis K (2009). Dre recombinase, like Cre, is a highly efficient site-specific recombinase in *E. coli*, mammalian cells and mice. Dis. Model. Mech..

[79] Miura H, Quadros RM, Gurumurthy CB, Ohtsuka M (2018). Easi-CRISPR for creating knock-in and conditional knockout mouse models using long ssDNA donors. Nat. Protoc..

[80] Tang W (2009). Faithful expression of multiple proteins via 2A-peptide self-processing: a versatile and reliable method for manipulating brain circuits. J. Neurosci..

[81] Smith RH, Levy JR, Kotin RM (2009). A simplified baculovirus-AAV expression vector system coupled with one-step affinity purification yields high-titer rAAV stocks from insect cells. Mol. Ther..

[82] Heinonen A-M (2014). Neuroprotection by rAAV-mediated gene transfer of bone morphogenic protein 7. BMC Neurosci..

[83] Tsaousidou E (2014). Distinct roles for JNK and IKK activation in agouti-related peptide neurons in the development of obesity and insulin resistance. Cell Rep..

[84] Jais A (2016). Myeloid-cell-derived VEGF maintains brain glucose uptake and limits cognitive impairment in obesity. Cell.

[85] Cízek J (2004). Fast and robust registration of PET and MR images of human brain. NeuroImage.

[86] Paxinos, G., Keith, B. J. & Franklin, M. A. *Paxinos and Franklin’s the Mouse Brain in Stereotaxic Coordinates*, *Compact* 4th edn (Academic Press, 2012).

[87] Green LA (1998). Noninvasive methods for quantitating blood time-activity curves from mouse PET images obtained with fluorine-18-fluorodeoxyglucose. J. Nucl. Med..

[88] Backes H (2011). Whiskers area as extracerebral reference tissue for quantification of rat brain metabolism using 18F-FDG PET: application to focal cerebral ischemia. J. Nucl. Med..

[89] Heiman M (2008). A translational profiling approach for the molecular characterization of CNS cell types. Cell.

[90] Heiman M, Kulicke R, Fenster RJ, Greengard P, Heintz N (2014). Cell-type-specific mRNA purification by translating ribosome affinity purification (TRAP). Nat. Protoc..

[91] Jais A (2020). PNOCARC neurons promote hyperphagia and obesity upon high-fat-diet feeding. Neuron.

[92] Knight ZA (2012). Molecular profiling of activated neurons by phosphorylated ribosome capture. Cell.

[93] Paeger L (2017). Antagonistic modulation of NPY/AgRP and POMC neurons in the arcuate nucleus by noradrenalin. eLife.

[94] Jiang H (2020). MCH neurons regulate permeability of the median eminence barrier. Neuron.

[95] Ewels, P. et al. *nf-core/rnaseq: nf-core/rnaseq version 1.4 ‘Gray Crocus Dachshund’* (Zenodo, accessed 15.01.2021); 10.5281/zenodo.3490660

[96] Garrido-Martín D, Palumbo E, Guigó R, Breschi A (2018). ggsashimi: sashimi plot revised for browser- and annotation-independent splicing visualization. PLoS Comput. Biol..

[97] Patro R, Duggal G, Love MI, Irizarry RA, Kingsford C (2017). Salmon provides fast and bias-aware quantification of transcript expression. Nat. Methods.

[98] Love MI, Huber W, Anders S (2014). Moderated estimation of fold change and dispersion for RNA-seq data with DESeq2. Genome Biol..

[99] Yates AD (2020). Ensembl 2020. Nucleic Acids Res..

[100] Durinck S, Spellman PT, Birney E, Huber W (2009). Mapping identifiers for the integration of genomic datasets with the R/Bioconductor package biomaRt. Nat. Protoc..

