# Supplementary material for: Insulin signalling in tanycytes gates hypothalamic insulin uptake and regulation of AgRP neuron activity
Source: Nat Metab. 2021 Dec 20;3(12):1662–79. doi: 10.1038/s42255-021-00499-0 (PMC8688146; doi:10.1038/s42255-021-00499-0)
Supplement: Supplementary file 1 — Reporting Summary [file 42255_2021_499_MOESM1_ESM.pdf]

nature research

Corresponding author(s): Jens C. Brüning

Last updated by author(s): Oct 7, 2021

# Reporting Summary

Nature Research wishes to improve the reproducibility of the work that we publish. This form provides structure for consistency and transparency in reporting. For further information on Nature Research policies, see our Editorial Policies and the Editorial Policy Checklist.

## Statistics

For all statistical analyses, confirm that the following items are present in the figure legend, table legend, main text, or Methods section.

| n/a | Confirmed | |
|---|---|---|
| ☐ | ☒ | The exact sample size (*n*) for each experimental group/condition, given as a discrete number and unit of measurement |
| ☐ | ☒ | A statement on whether measurements were taken from distinct samples or whether the same sample was measured repeatedly |
| ☐ | ☒ | The statistical test(s) used AND whether they are one- or two-sided<br>*Only common tests should be described solely by name; describe more complex techniques in the Methods section.* |
| ☐ | ☒ | A description of all covariates tested |
| ☐ | ☒ | A description of any assumptions or corrections, such as tests of normality and adjustment for multiple comparisons |
| ☐ | ☒ | A full description of the statistical parameters including central tendency (e.g. means) or other basic estimates (e.g. regression coefficient) AND variation (e.g. standard deviation) or associated estimates of uncertainty (e.g. confidence intervals) |
| ☐ | ☒ | For null hypothesis testing, the test statistic (e.g. *F*, *t*, *r*) with confidence intervals, effect sizes, degrees of freedom and *P* value noted<br>*Give P values as exact values whenever suitable.* |
| ☒ | ☐ | For Bayesian analysis, information on the choice of priors and Markov chain Monte Carlo settings |
| ☒ | ☐ | For hierarchical and complex designs, identification of the appropriate level for tests and full reporting of outcomes |
| ☒ | ☐ | Estimates of effect sizes (e.g. Cohen's *d*, Pearson's *r*), indicating how they were calculated |

*Our web collection on statistics for biologists contains articles on many of the points above.*

## Software and code

Policy information about availability of computer code

| | |
|---|---|
| Data collection | Indirect calorimetry data were collected using PhenoMaster, TSE systems ver. 5.6.6<br>Confocal images were collected on Leica SP8 microscope, controlled by LasX software.<br>PET images were acquire using Inveon Aquisition Workstation (IAW) ver 2.0.0.1050, Vinci ver. 5.06<br>Fiber photometry were acquired in RZ5P real-time processor (Tucker-Davis Technologies, TDT) and Synapse (ver. 95-43718P). |
| Data analysis | Data analysis and visualization: Prism ver. 8 (GraphPad)<br>RNA-sequencing and data visualization: R ver 3.5.3. (https://www.R-project.org/), R studio ver 1.1.463, nforce ver 1.4, Salmon ver 0.14.1, DESeq ver 1.30.0.<br>Image analysis: Fiji ver 2.0.0-rc-69/1.52n.<br>Data analysis: Microsoft Excel ver. 16.45.<br>For PET we use our own software for kinetic modeling and image analysis written in IDL and C, IDL ver. 8.5.1, C-compiler gcc ver.7.5.0 and used as described previously (Jais et al., 2016, 2020).<br>Fiber photometry data were analysed using custom MATLAB script, MATLAB 2014b (8.4.0.150421). |

For manuscripts utilizing custom algorithms or software that are central to the research but not yet described in published literature, software must be made available to editors and reviewers. We strongly encourage code deposition in a community repository (e.g. GitHub). See the Nature Research guidelines for submitting code & software for further information.

# Data

Policy information about availability of data

All manuscripts must include a data availability statement. This statement should provide the following information, where applicable:

- Accession codes, unique identifiers, or web links for publicly available datasets
- A list of figures that have associated raw data
- A description of any restrictions on data availability

Raw RNA-seq data have been deposited in the NCBI Gene Expression Omnibus under accession code: GSE185074. The gene-level quantification was carried out using the reference genome GRCm38. Other raw data is available under reasonable request to the corresponding author.

# Field-specific reporting

Please select the one below that is the best fit for your research. If you are not sure, read the appropriate sections before making your selection.

☒ Life sciences ☐ Behavioural & social sciences ☐ Ecological, evolutionary & environmental sciences

For a reference copy of the document with all sections, see nature.com/documents/nr-reporting-summary-flat.pdf

# Life sciences study design

All studies must disclose on these points even when the disclosure is negative.

| | |
|---|---|
| Sample size | No statistical methods were used to pre-determine sample sizes.<br>Sample size for each study was determined based on prior work by authors and according to the standard in the field and previously reported: (Biglari et al. 2021, Jiang et al. 2020, Jais et al. 2016, Vogt et al. 2015, Könner et al. 2007). |
| Data exclusions | Mice were excluded from sample, if they did not survive stereotactical surgical procedures or fiber placement were wrong for in vivo fiber photometry studies. For “Hyperinsulinemic-euglycemic clamp studies in awake mice” experiments only mice, for which sufficient blood for analysis could not be collected during the clamp procedure, were excluded from analysis. |
| Replication | For all experiments, unless otherwise stated, every mouse represents a replicate (n). The number of replicates is mentioned in th legends of figures and extended data. Furthermore, to further substantiate findings and confirm the findings across methodologies and cohorts, metabolic phenotyping experiments were performed in at least 2 independent cohorts and this was successful. For RNA-seq individual mice were collected from several cohorts and four to seven mie were pooled per n.<br>For IHC (pAKT, pSTAT3, AF488) and RNAscope experiments requiring quantification 3 to 5 slices per individual animal were quantified and averaged for data point. For other microscopy experiments generating representative images 2 to 5 individual mice (as indicated in the legends) were assessed. |
| Randomization | Mice were randomly assigned to the experimental or control group. Further allocations were based on body weight to match the mean of the group. |
| Blinding | During the phenotyping (GTT, ITT, food intake, calorimetry, leptin, ghrelin sensitivity and behavioral testing, in vivo fiber photometry studies ) as well as analysis of immunohistochemistries and in situ hybridizations were performed blinded, and genotype was only disclosed after data analysis.  BacTRAP-based ribosomal profiling of tanycytes experiment were blinded until the immunopurification step, were the extracted hypothalami of individual mice were randomly pooled (4-7/n) according to their genotypes or treatment (HFD vs. NCD). RNA-seq was then further performed in a blinded manner.<br>PET experiments were not blinded, due to careful randomization to treatment, set-up and measurement time of the day. Here animals were distributed based on their genotype, treatment (saline vs. insulin), place in the scanner during the day of the measurement to control for any confounding effects. |

# Reporting for specific materials, systems and methods

We require information from authors about some types of materials, experimental systems and methods used in many studies. Here, indicate whether each material, system or method listed is relevant to your study. If you are not sure if a list item applies to your research, read the appropriate section before selecting a response.

## Materials & experimental systems

| n/a | Involved in the study |
| --- | --- |
| ☐ | ☒ Antibodies |
| ☒ | ☐ Eukaryotic cell lines |
| ☒ | ☐ Palaeontology and archaeology |
| ☐ | ☒ Animals and other organisms |
| ☒ | ☐ Human research participants |
| ☒ | ☐ Clinical data |
| ☒ | ☐ Dual use research of concern |

## Methods

| n/a | Involved in the study |
| --- | --- |
| ☒ | ☐ ChIP-seq |
| ☒ | ☐ Flow cytometry |
| ☒ | ☐ MRI-based neuroimaging |

# Antibodies

Antibodies used

rabbit anti-pAKT (#4060, Cell Signalling), rabbit anti-pSTAT3 ( #9145, Cell Signalling), Anti-Rabbit IgG (Goat) HRP-Labeled (#NEF812001EA, Perkin Elmer), rabbit Alexa-Flour 488 ( #A11094, ThermoFisher Scientific), rabbit anti-RFP (#R10367, Thermo Fisher), chicken anti-GFP (#13970, Abcam)

Validation

rabbit anti-pAKT (#4060, Cell Signalling): cited 4784 times on manufacturers webstie, validated for WB, IP, IHC, IF, F.
https://www.cellsignal.com/products/primary-antibodies/phospho-akt-ser473-d9e-xp-rabbit-mab/4060

rabbit anti-pSTAT3 ( #9145, Cell Signalling): 1669 citations on manufacturers website, validated for WB, IP, IHC, IF, F.
https://www.cellsignal.com/products/primary-antibodies/phospho-stat3-tyr705-d3a7-xp-rabbit-mab/9145

Anti-Rabbit IgG (Goat) HRP-Labeled (#NEF812001EA, Perkin Elmer): 88 citations, validated for WB, IHC, IF.
https://www.citeab.com/antibodies/8893045-nef812001ea-anti-rabbit-igg-goat-hrp-labeled-1mg
https://www.perkinelmer.com/product/anti-rabbit-igg-hrp-labeled-goat-nef812001ea

rabbit Alexa-Flour 488 ( #A11094, ThermoFisher Scientific): 96 references on manufacturers website, validated for WB, IP, ICC, IHC, IF, F.
https://www.thermofisher.com/antibody/product/Alexa-Fluor-488-Antibody-Polyclonal/A-11094

rabbit anti-RFP (#R10367, Thermo Fisher): 55 citations on manufacturers website, validated for WB, IP, ICC, IHC-P, IHC-F, IF, F
https://www.thermofisher.com/antibody/product/RFP-Antibody-Polyclonal/R10367

chicken anti-GFP (#ab13970, Abcam): 90 citations on manufacturers website, validated for WB, ICC, F
https://www.abcam.com/gfp-antibody-ab13970.html

# Animals and other organisms

Policy information about studies involving animals; ARRIVE guidelines recommended for reporting animal research

Laboratory animals

Mice were housed in groups of 3–5 at 22°C – 24°C using a 12 h light/1h dark cycle in individually ventilated cages (IVCs). Animals were fed ad libitum chow diet ssniff® (V1554, Sniff Spezialdiäten GmbH) or high-fat diet (HFD; EF D12492-(I), Sniff Spezialdiäten GmbH) starting age of 4 weeks for until age of 16 – 18 weeks, and water, and food was only withdrawn, if required for an experiment. All mouse lines were established on a C57Bl/6 background. We used only male, unless otherwise indicated, littermate mice that were age-matched between experimental groups.
IR fl/fl mice were purchased from Jackson Laboratories. Slco1c1-CreERT2 (Ridder et al. 2011) were kindly provided by Prof. M. Schwaninger. ROSA26 tdTomato fl was purchased form Jackson Laboratories. ROSA26lSTOPlZsGreen fl,D/fl,D and ROSA26lSTOPlL10a-GFP fl,D/fl,D have been recently generated in the lab and described (Biglari et. al. 2021). Agrp-p2a-Dre were kindly provided by Prof. B. B. Lowell.

Wild animals

not used

Field-collected samples

not used

Ethics oversight

All animal procedures were conducted in compliance with protocols approved by local government authorities (Bezirksregierung Köln). Permissions for experiments and to maintain and breed mice was issued by the Department for Environment and Consumer Protection-Veterinary Section, Cologne, North Rhine-Westphalia, Germany.

Note that full information on the approval of the study protocol must also be provided in the manuscript.
