# Supplementary figures and images for: Insulin signalling in tanycytes gates hypothalamic insulin uptake and regulation of AgRP neuron activity
Source: Nat Metab. 2021 Dec 20;3(12):1662–79. doi: 10.1038/s42255-021-00499-0 (PMC8688146; doi:10.1038/s42255-021-00499-0)

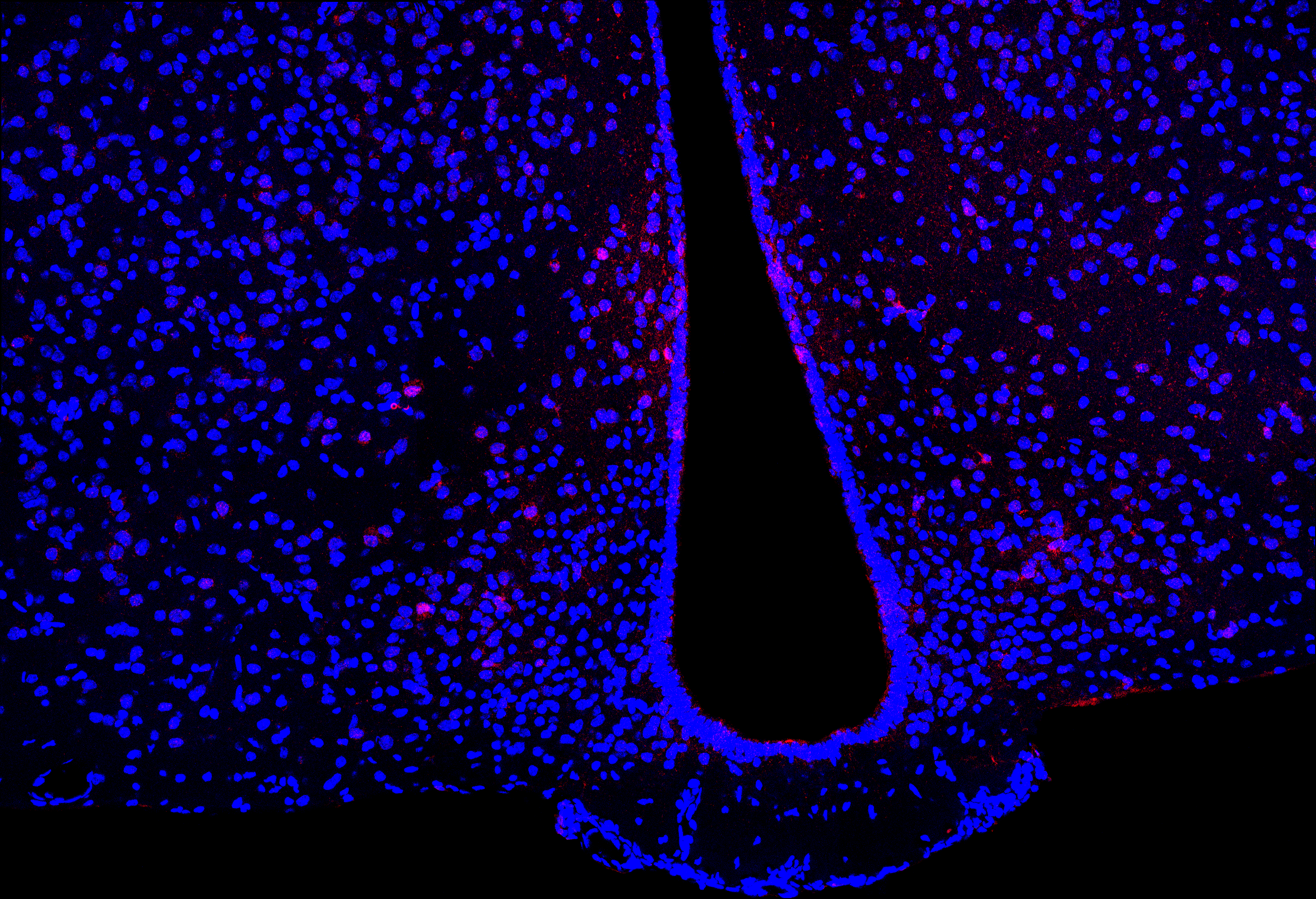

Supplement: Source Data Images Fig. 1 — Source data images. [file 42255_2021_499_MOESM3_ESM.zip › Fig 1h IR-GFP control HFD 0 min.tif]

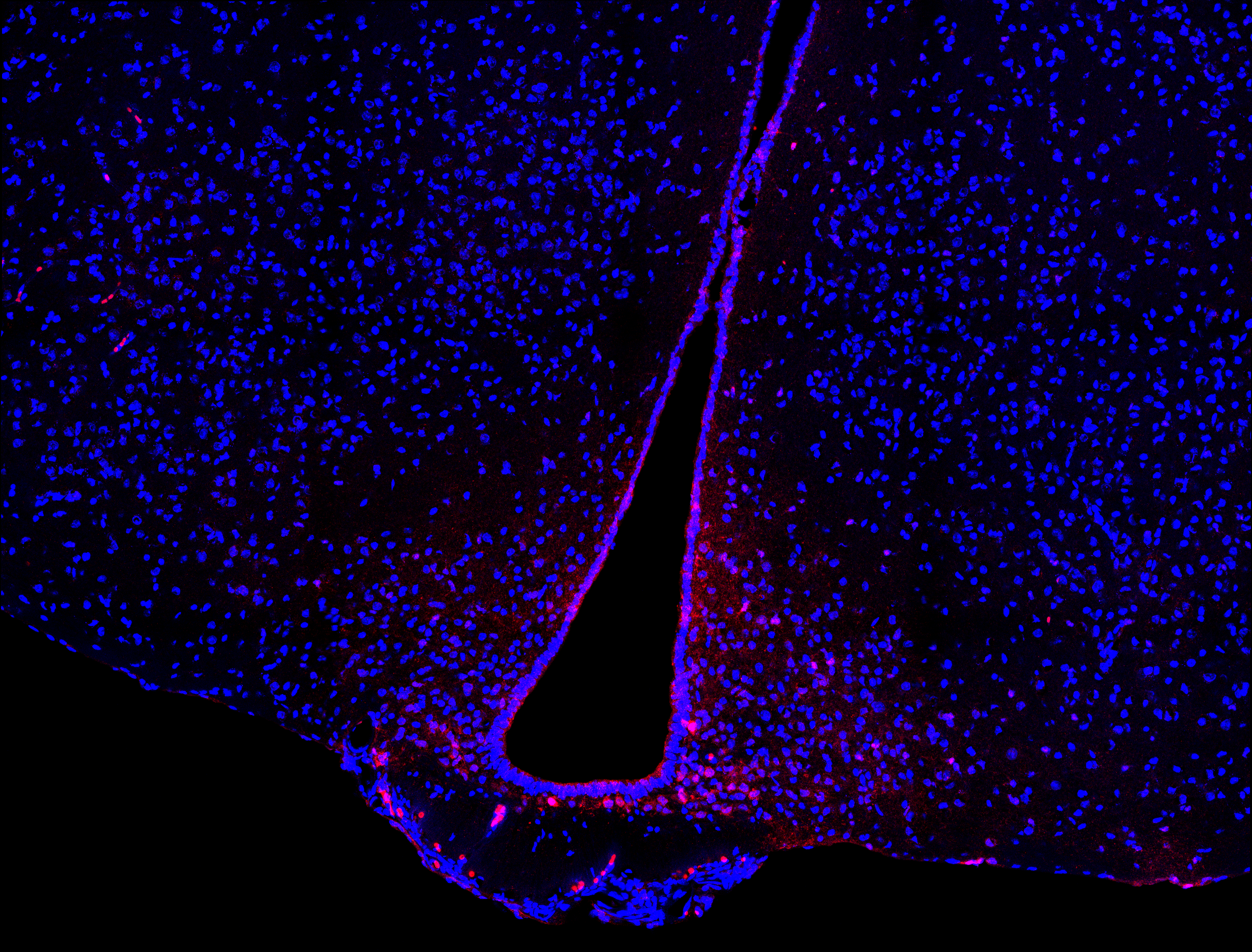

Supplement: Source Data Images Fig. 1 — Source data images. [file 42255_2021_499_MOESM3_ESM.zip › Fig 1h IR-Tan KO 30 min.tif]

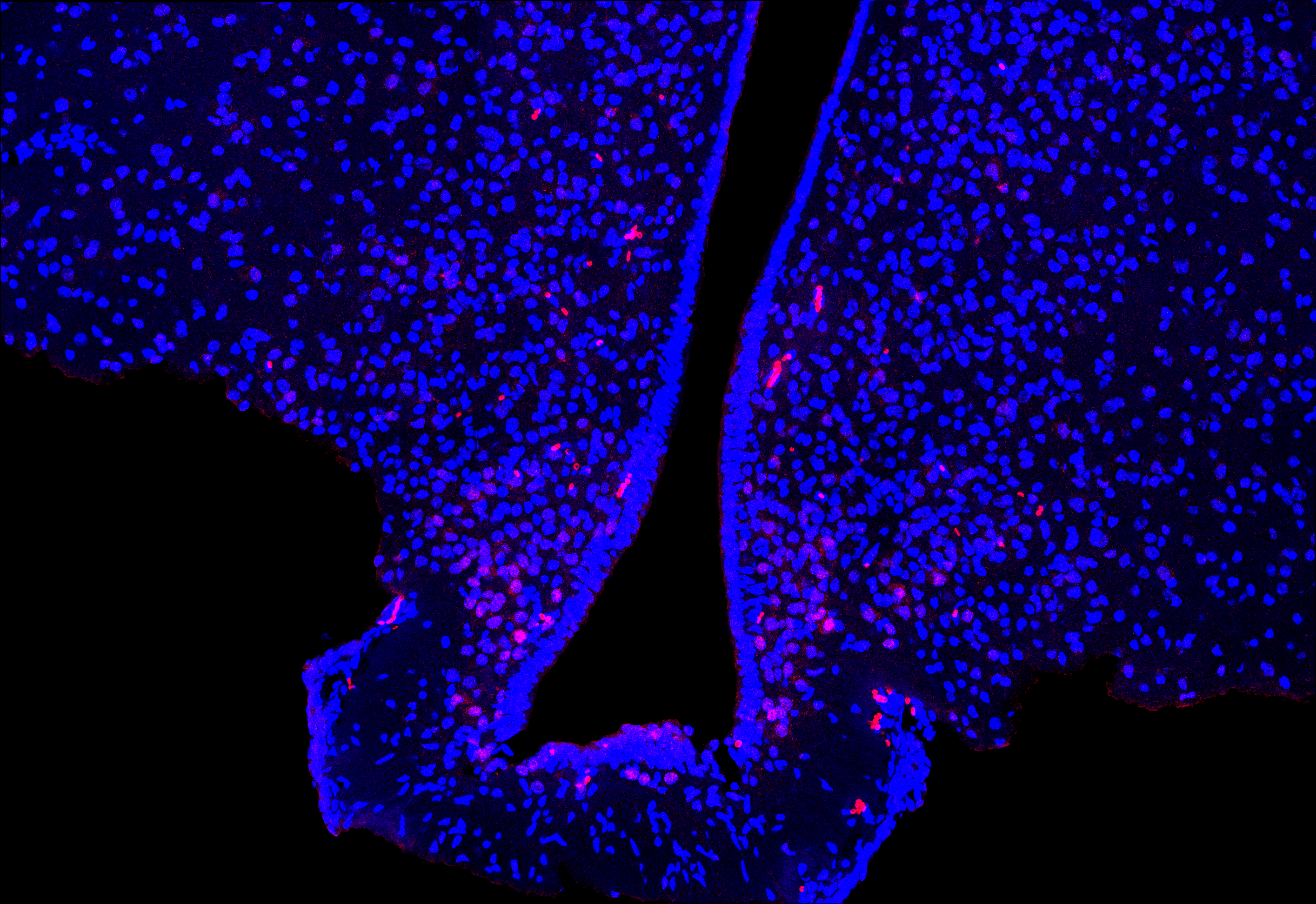

Supplement: Source Data Images Fig. 1 — Source data images. [file 42255_2021_499_MOESM3_ESM.zip › Fig 1b IR-GFP control HFD 10 min.tif]

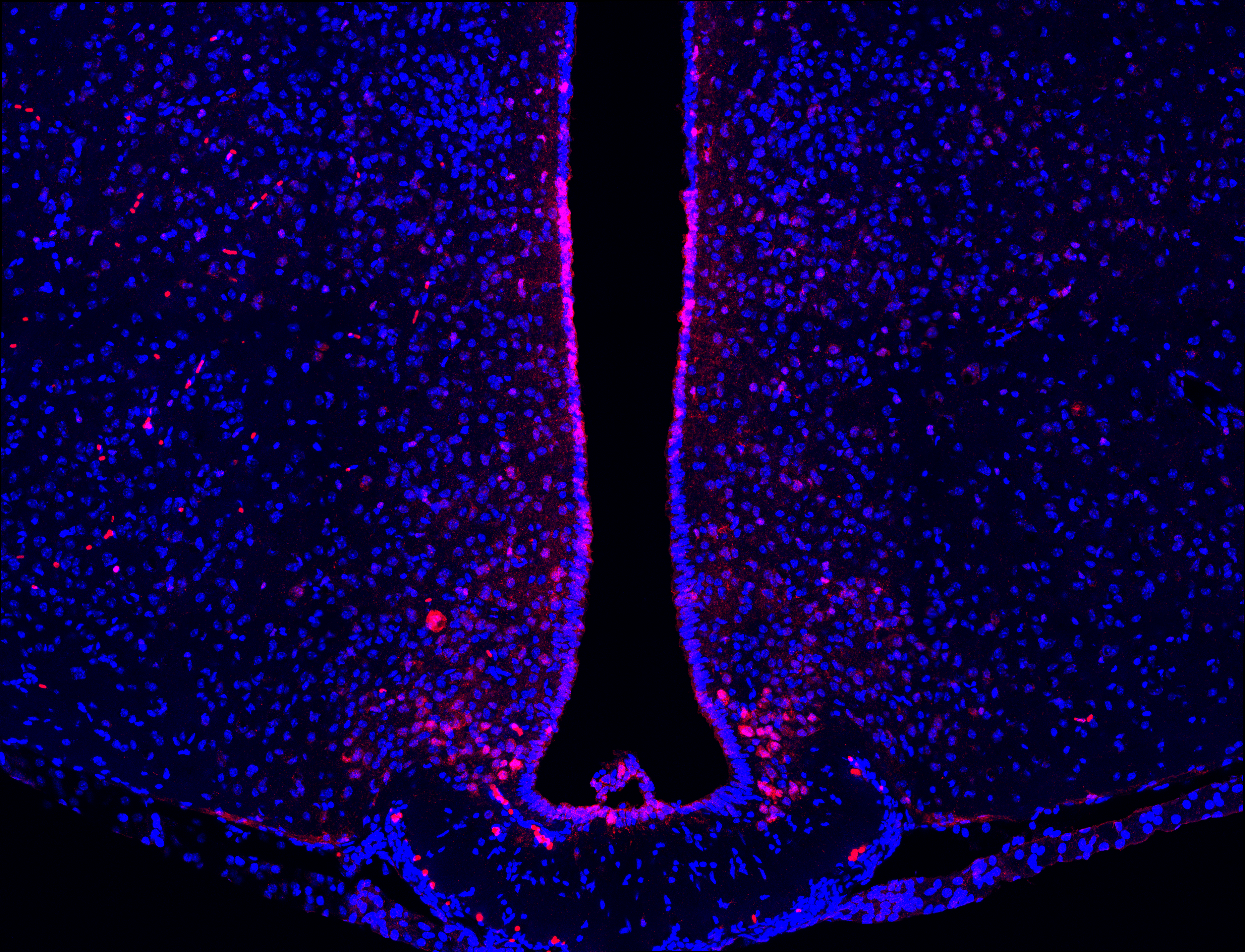

Supplement: Source Data Images Fig. 1 — Source data images. [file 42255_2021_499_MOESM3_ESM.zip › Fig 1h IR-Tan KO 20 min.tif]

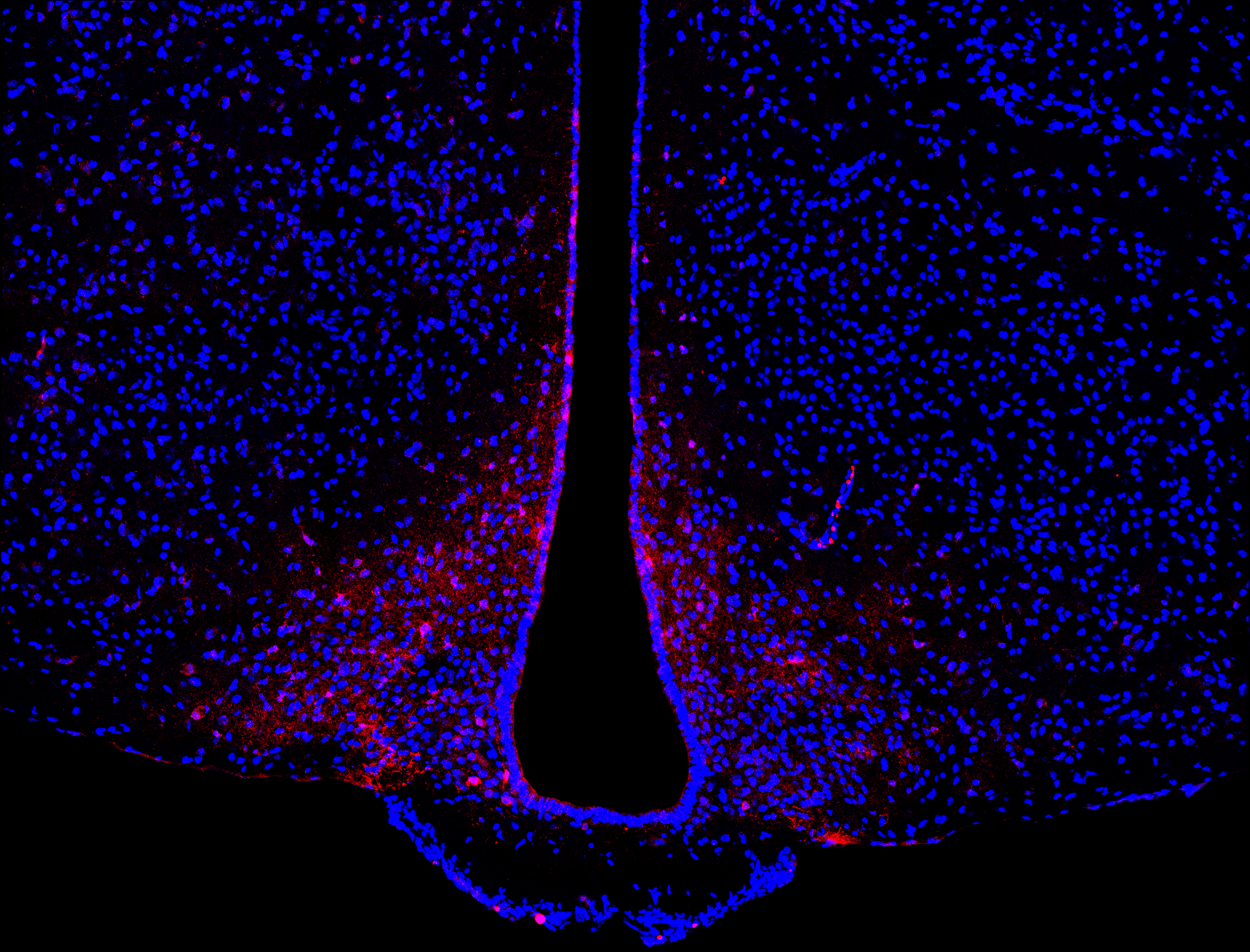

Supplement: Source Data Images Fig. 1 — Source data images. [file 42255_2021_499_MOESM3_ESM.zip › Fig 1h IR-GFP control 0 min.tif]

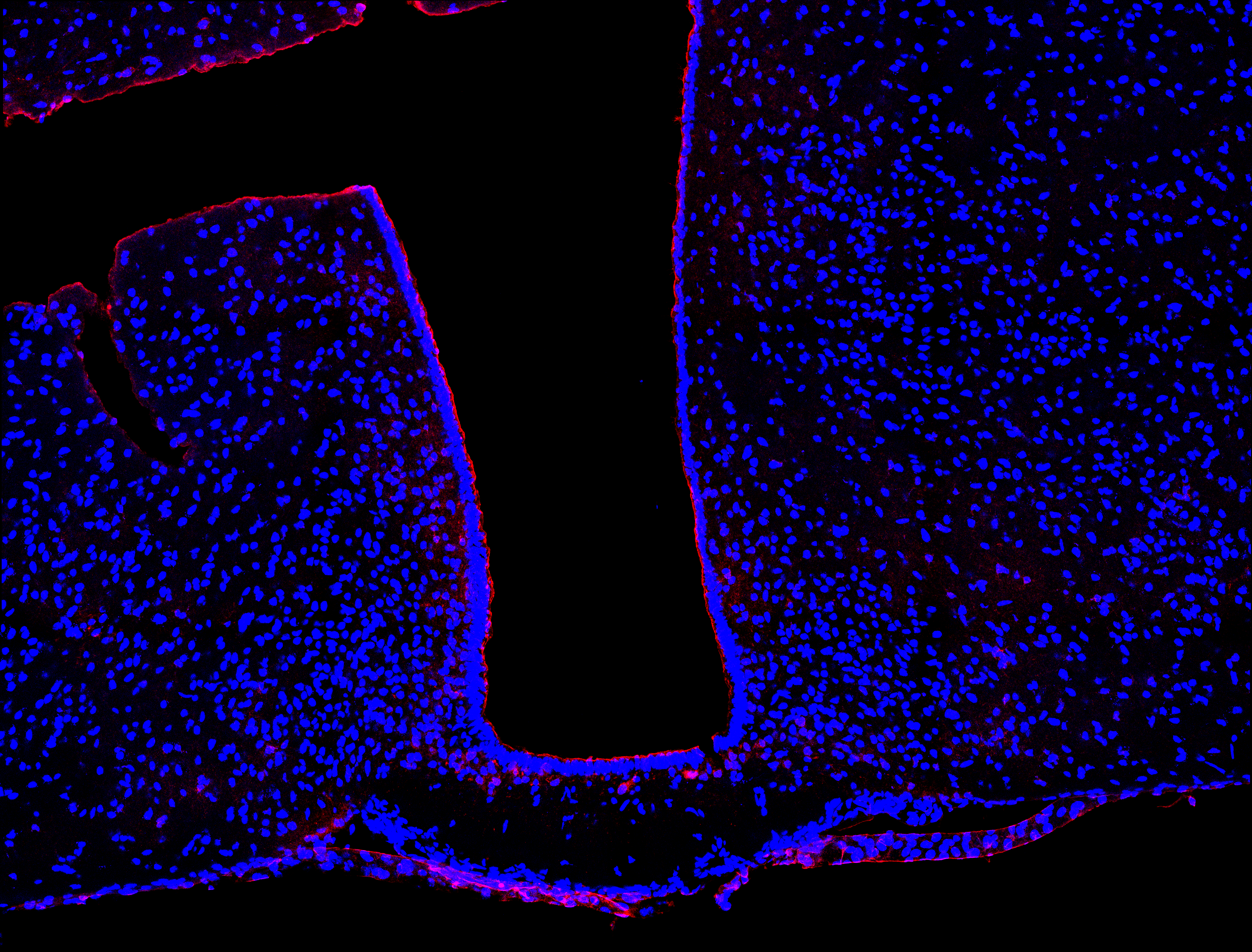

Supplement: Source Data Images Fig. 1 — Source data images. [file 42255_2021_499_MOESM3_ESM.zip › Fig 1h IR-Tan KO 5 min.tif]

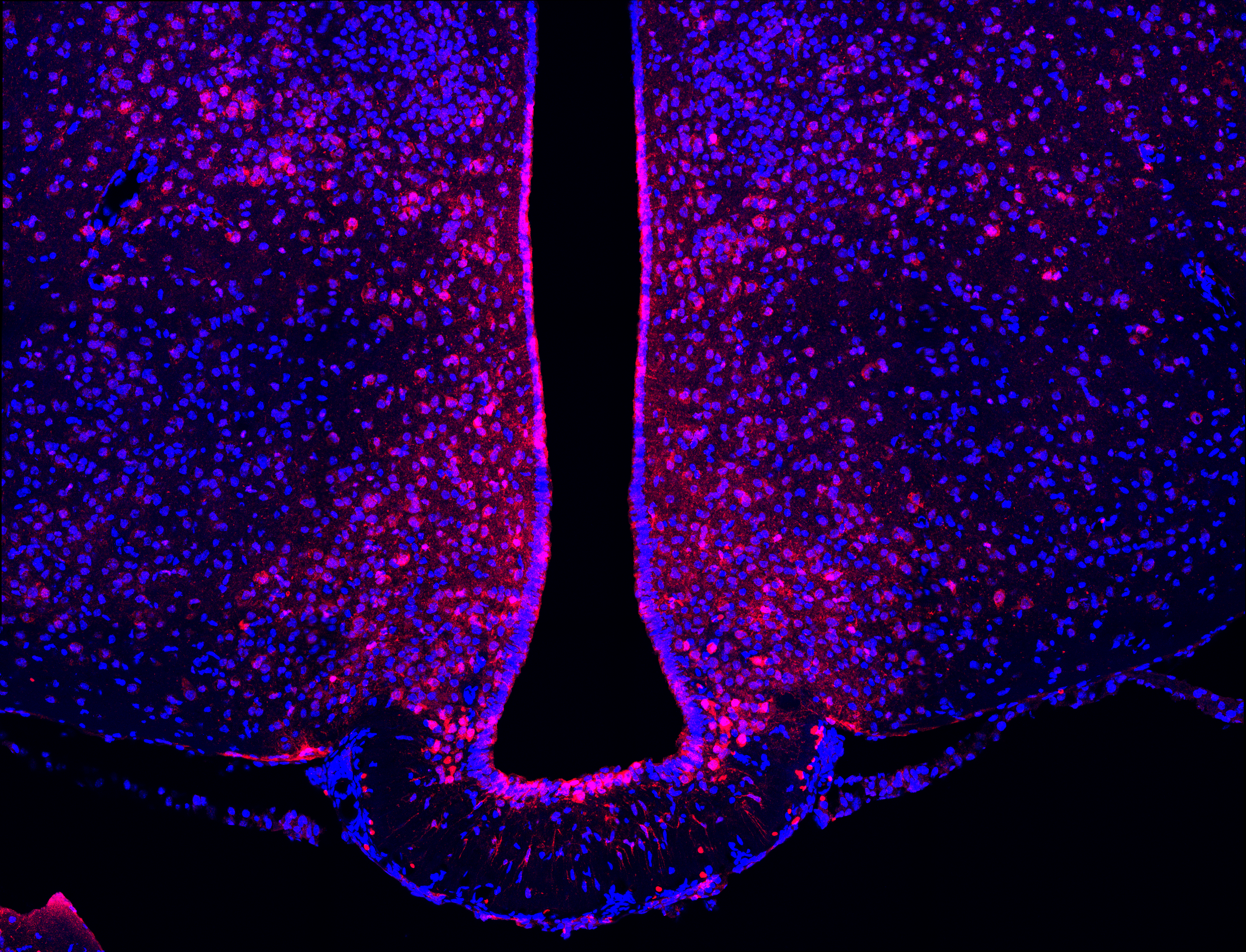

Supplement: Source Data Images Fig. 1 — Source data images. [file 42255_2021_499_MOESM3_ESM.zip › Fig 1h IR-GFP control 10 min.tif]

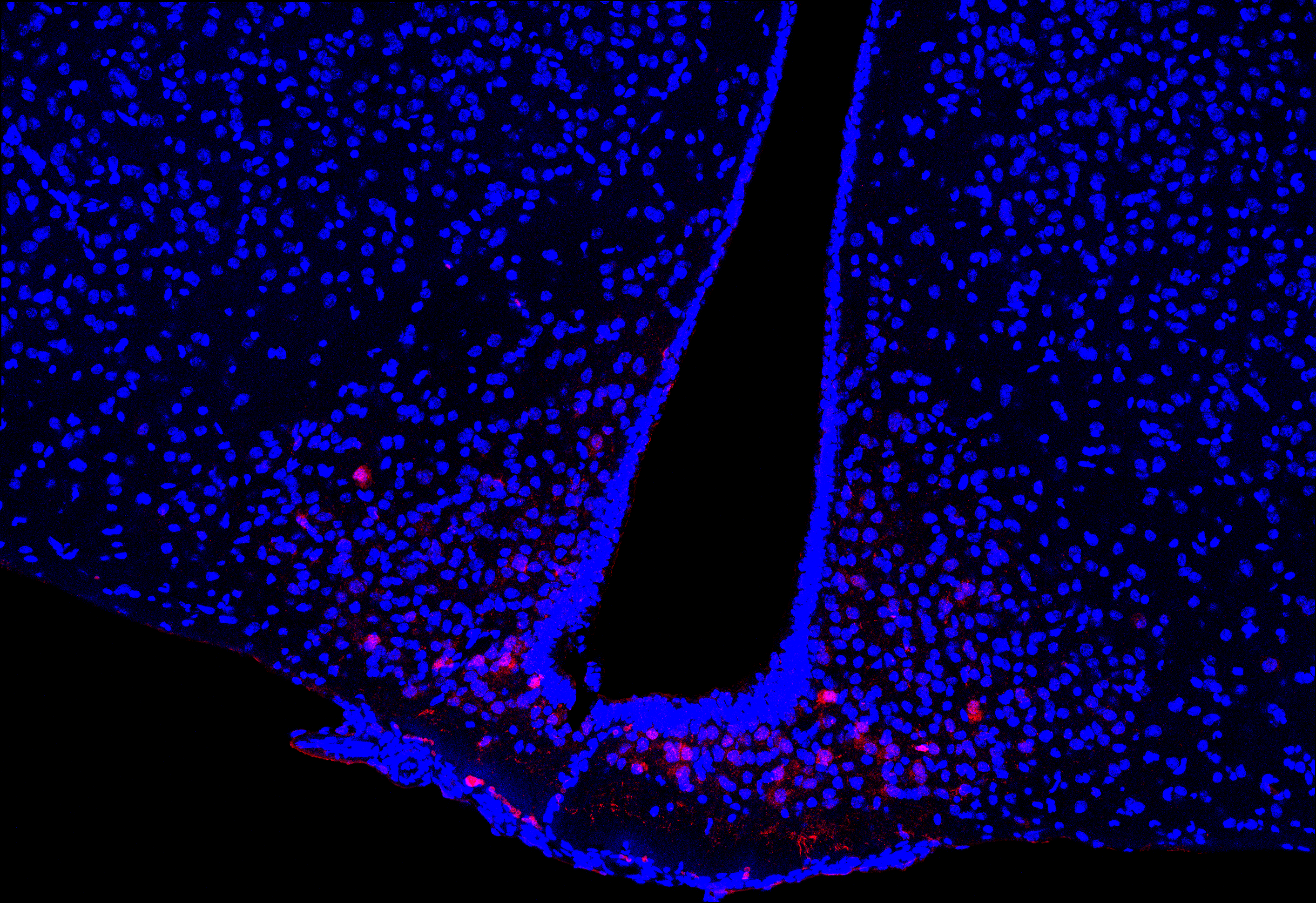

Supplement: Source Data Images Fig. 1 — Source data images. [file 42255_2021_499_MOESM3_ESM.zip › Fig 1h IR-GFP control HFD 30 min.tif]

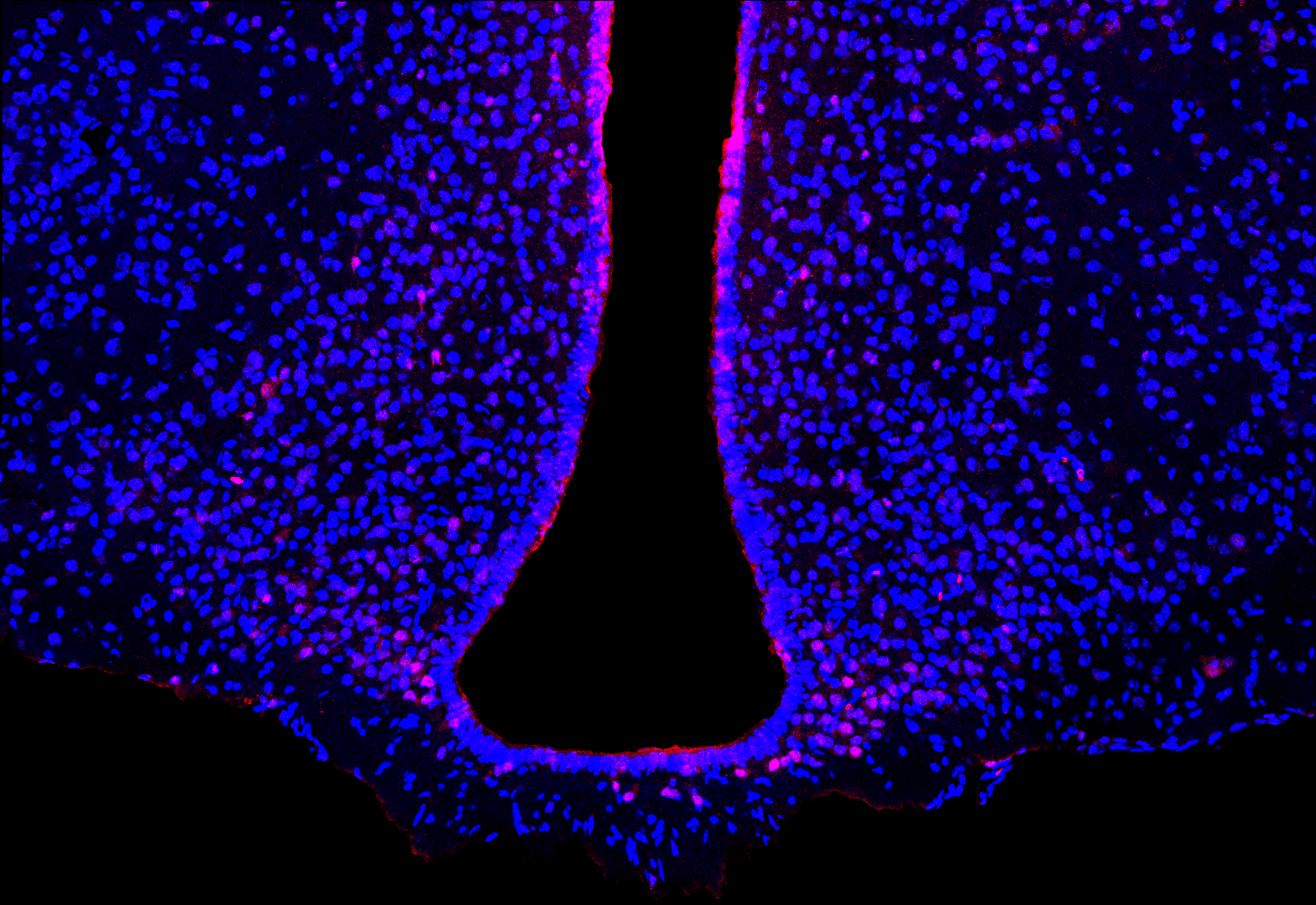

Supplement: Source Data Images Fig. 1 — Source data images. [file 42255_2021_499_MOESM3_ESM.zip › Fig 1h IR-GFP control HFD 20 min.tif]

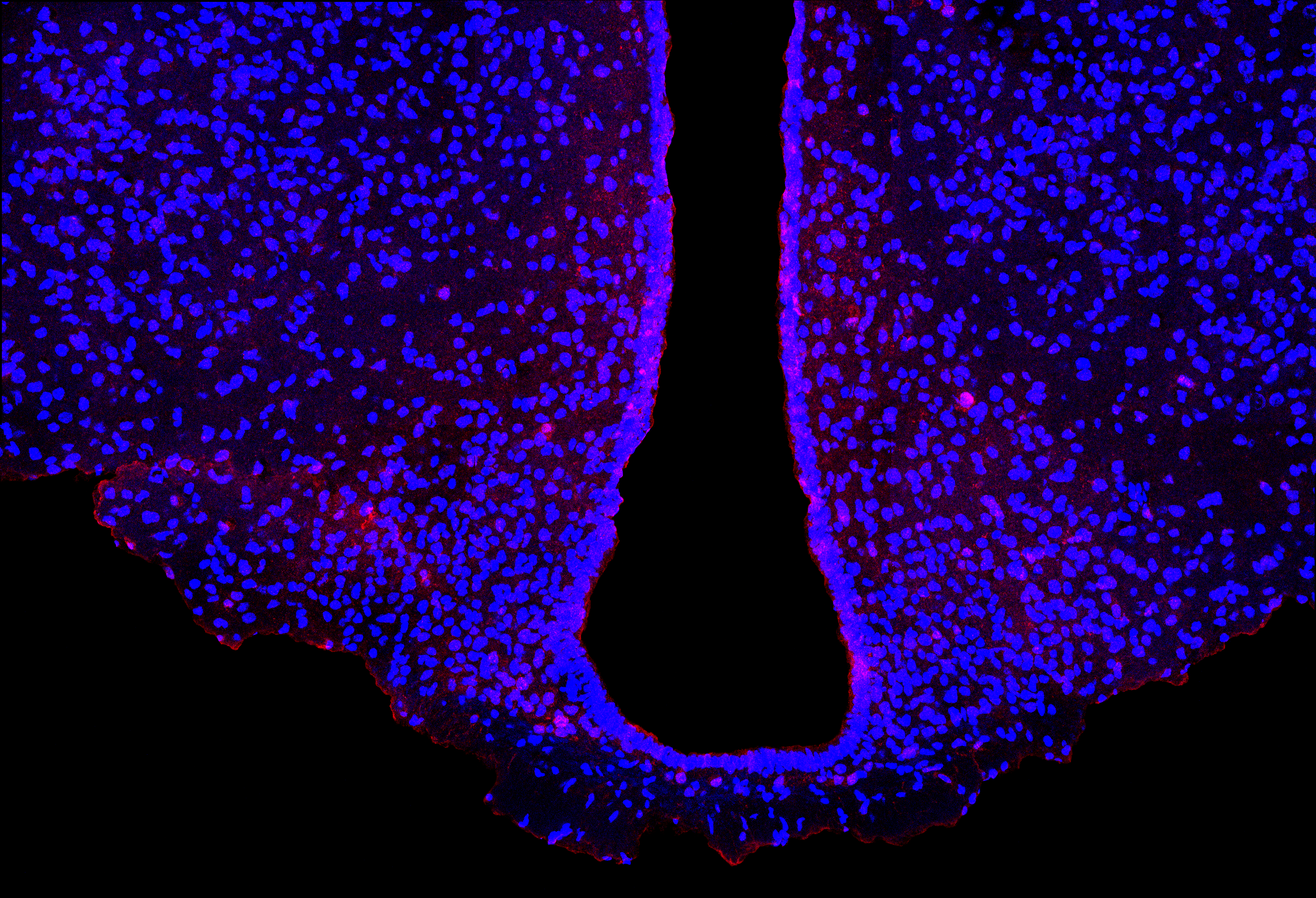

Supplement: Source Data Images Fig. 1 — Source data images. [file 42255_2021_499_MOESM3_ESM.zip › Fig 1b IR-GFP control HFD 5 min.tif]

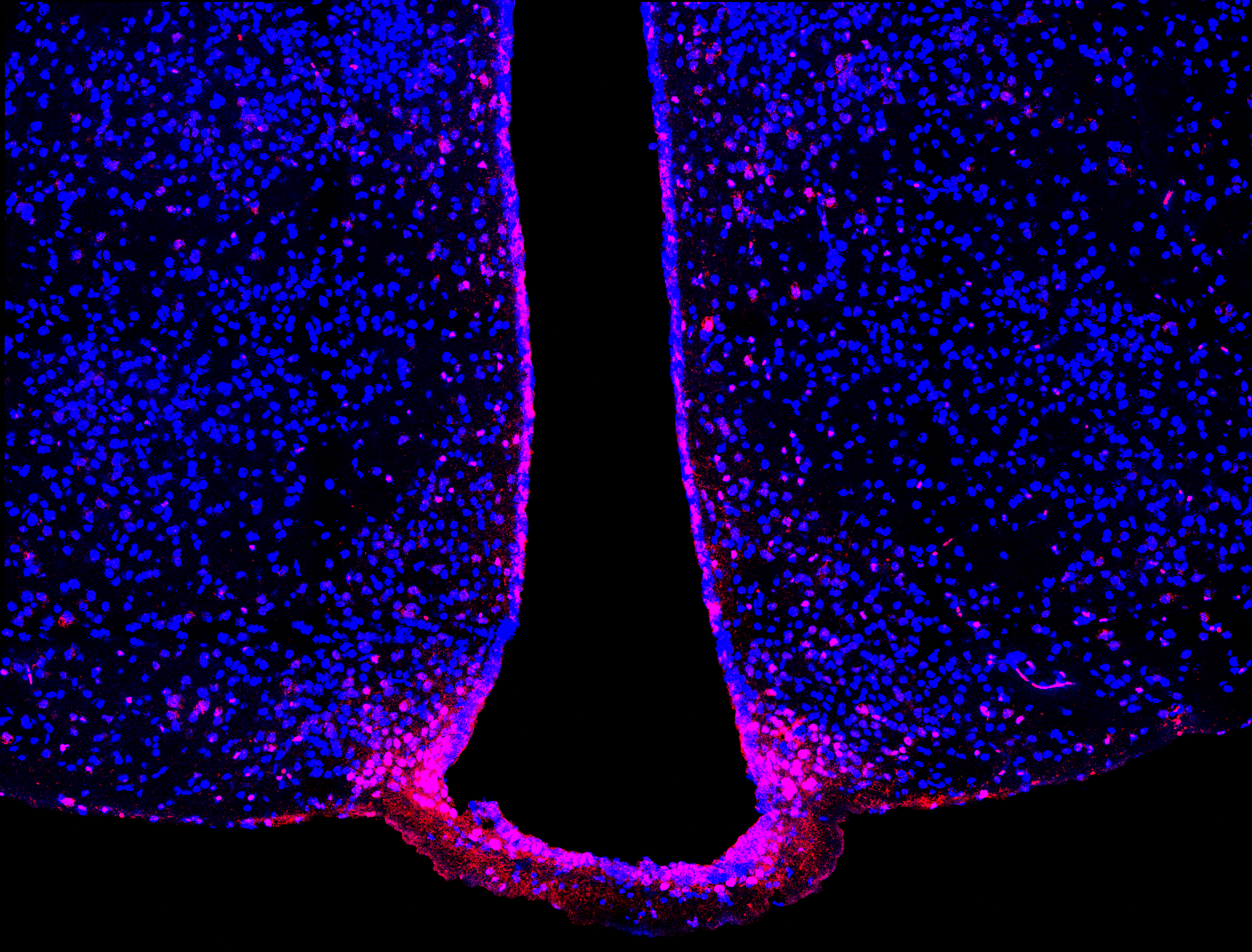

Supplement: Source Data Images Fig. 1 — Source data images. [file 42255_2021_499_MOESM3_ESM.zip › Fig 1h IR-GFP control 20 min 2.tif]

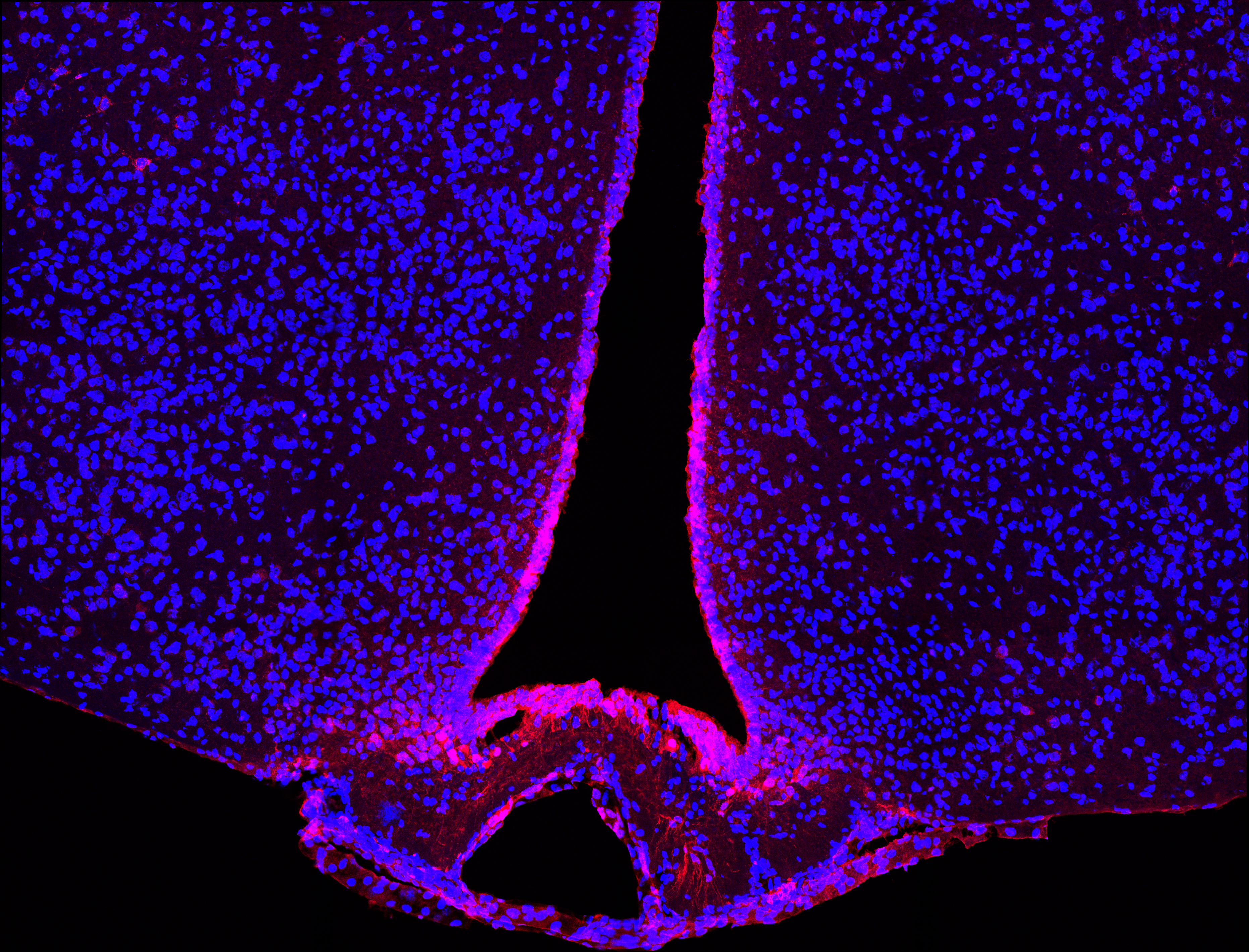

Supplement: Source Data Images Fig. 1 — Source data images. [file 42255_2021_499_MOESM3_ESM.zip › Fig 1b IR-GFP control 10 min.tif]

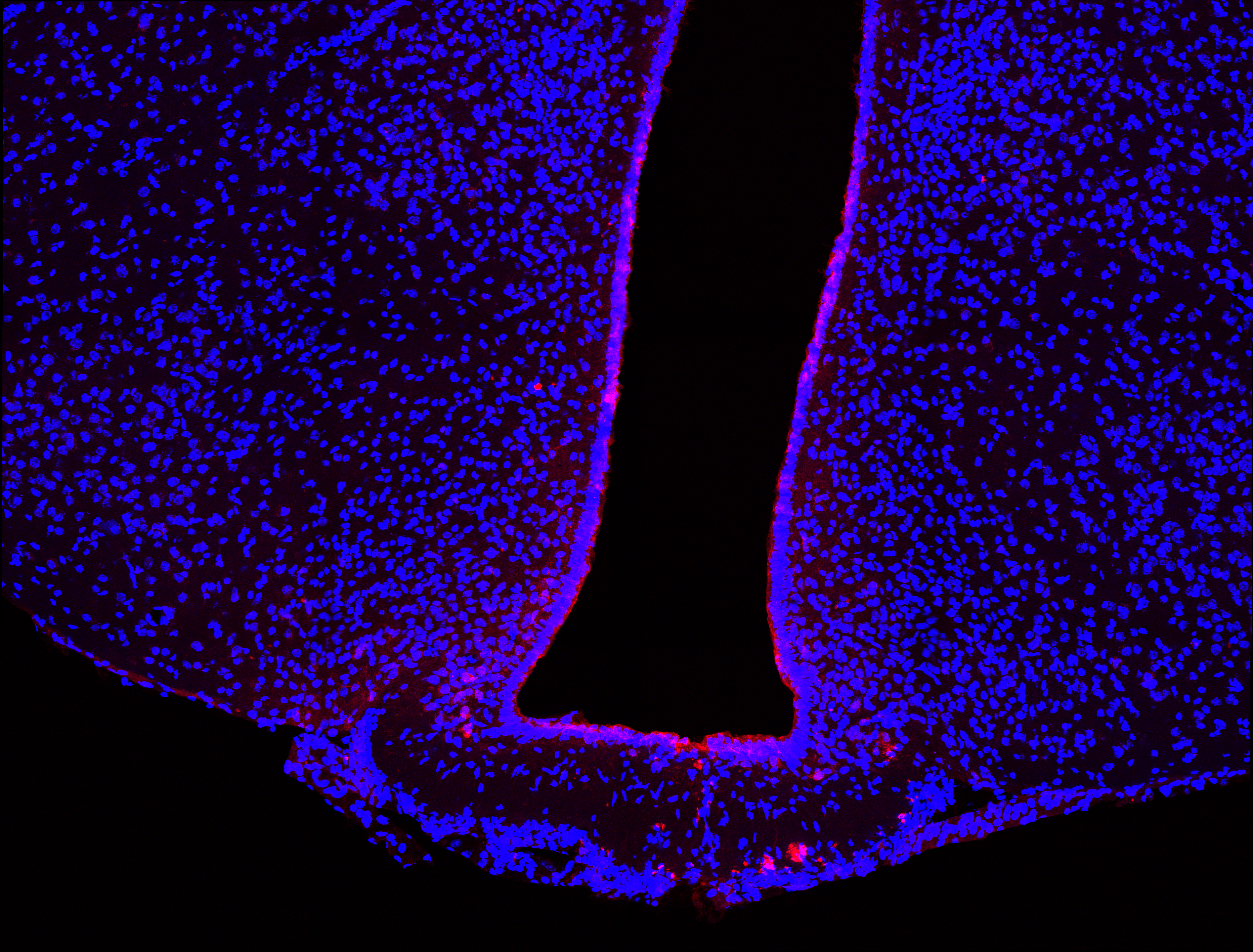

Supplement: Source Data Images Fig. 1 — Source data images. [file 42255_2021_499_MOESM3_ESM.zip › Fig 1b IR-Tan KO 5 min.tif]

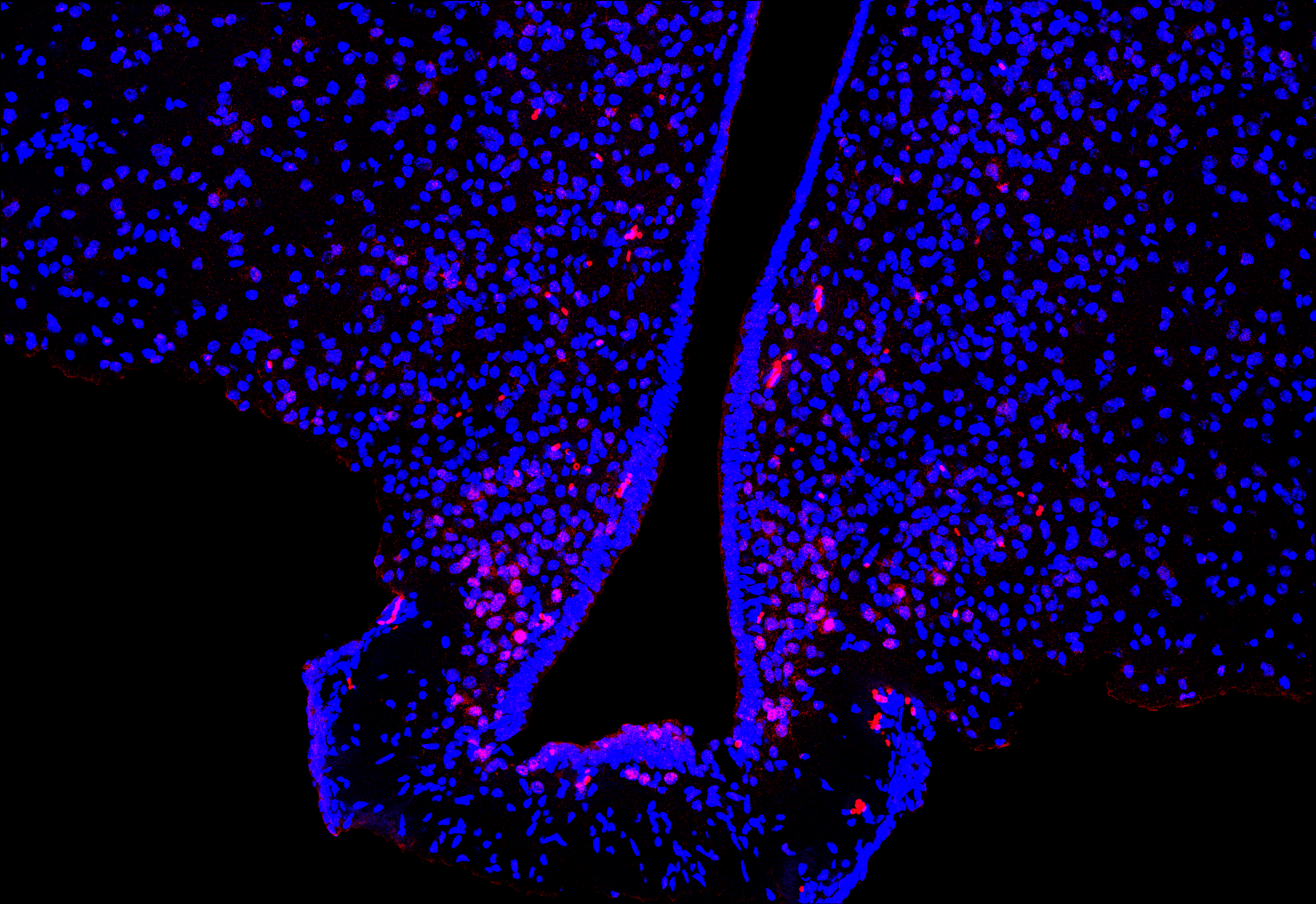

Supplement: Source Data Images Fig. 1 — Source data images. [file 42255_2021_499_MOESM3_ESM.zip › Fig 1h IR-GFP control HFD 10 min.tif]

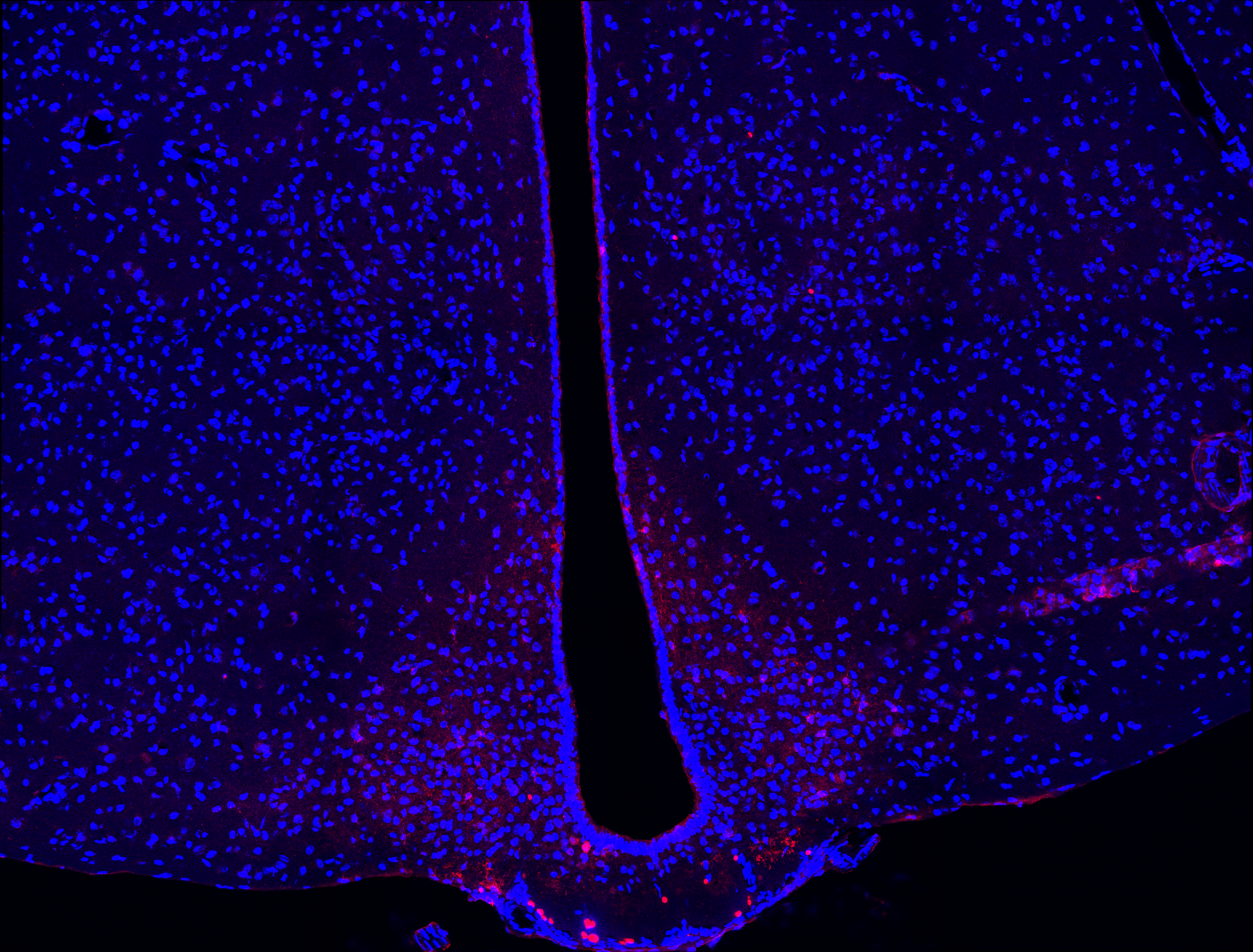

Supplement: Source Data Images Fig. 1 — Source data images. [file 42255_2021_499_MOESM3_ESM.zip › Fig 1b IR-Tan KO 0 min.tif]

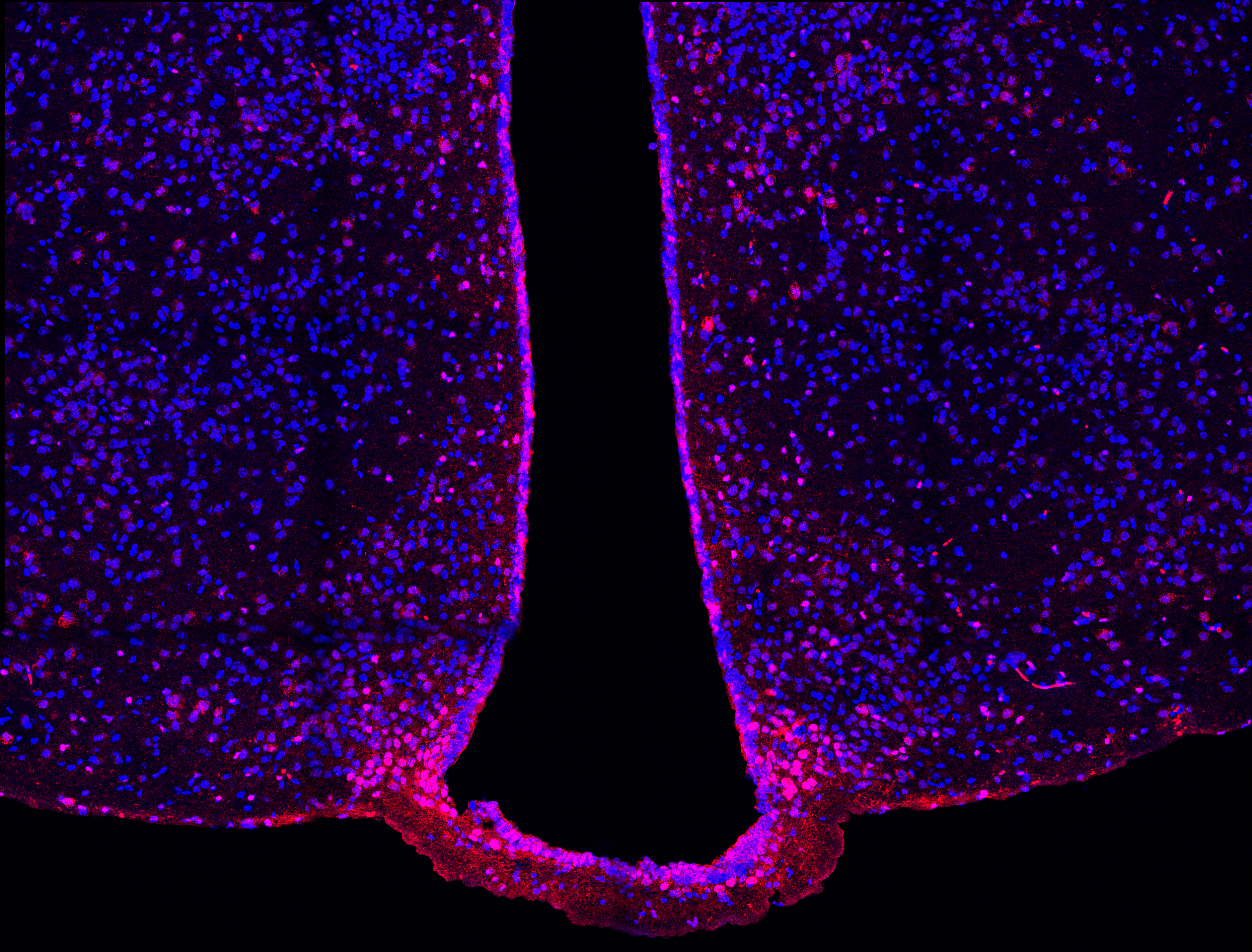

Supplement: Source Data Images Fig. 1 — Source data images. [file 42255_2021_499_MOESM3_ESM.zip › Fig 1h IR-GFP control 20 min.tif]

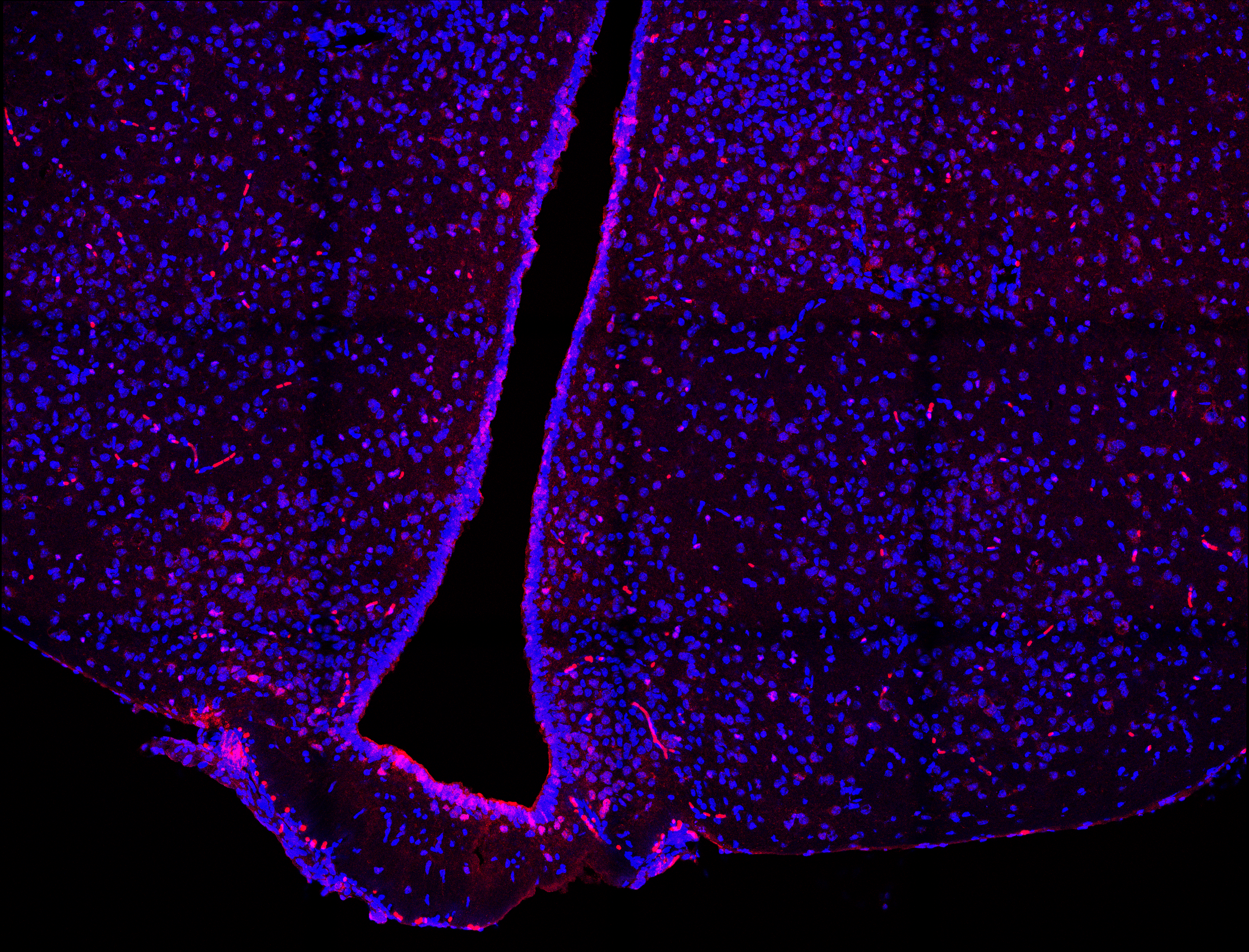

Supplement: Source Data Images Fig. 1 — Source data images. [file 42255_2021_499_MOESM3_ESM.zip › Fig 1h IR-GFP control 30 min.tif]

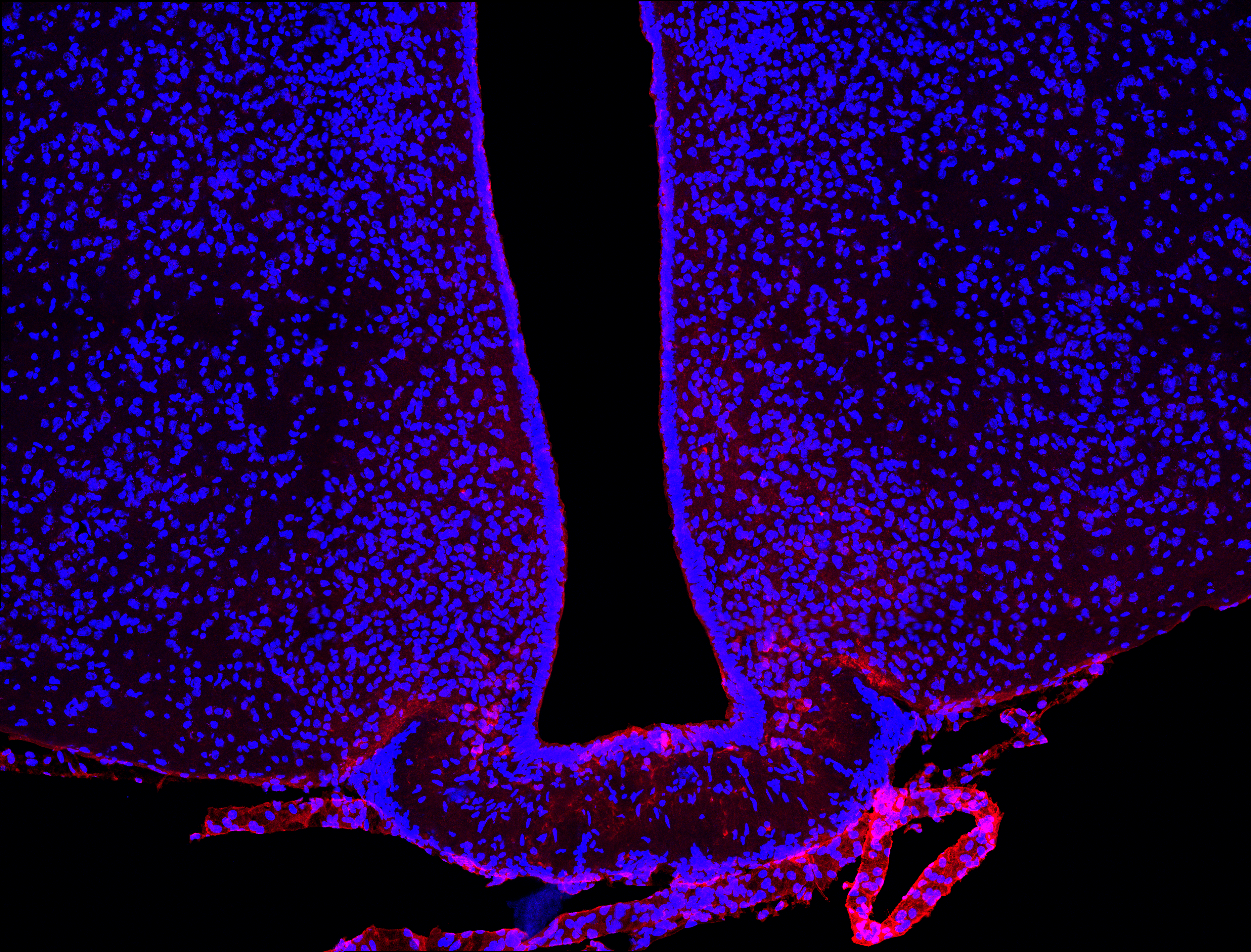

Supplement: Source Data Images Fig. 1 — Source data images. [file 42255_2021_499_MOESM3_ESM.zip › Fig 1b IR-Tan KO 10 min.tif]

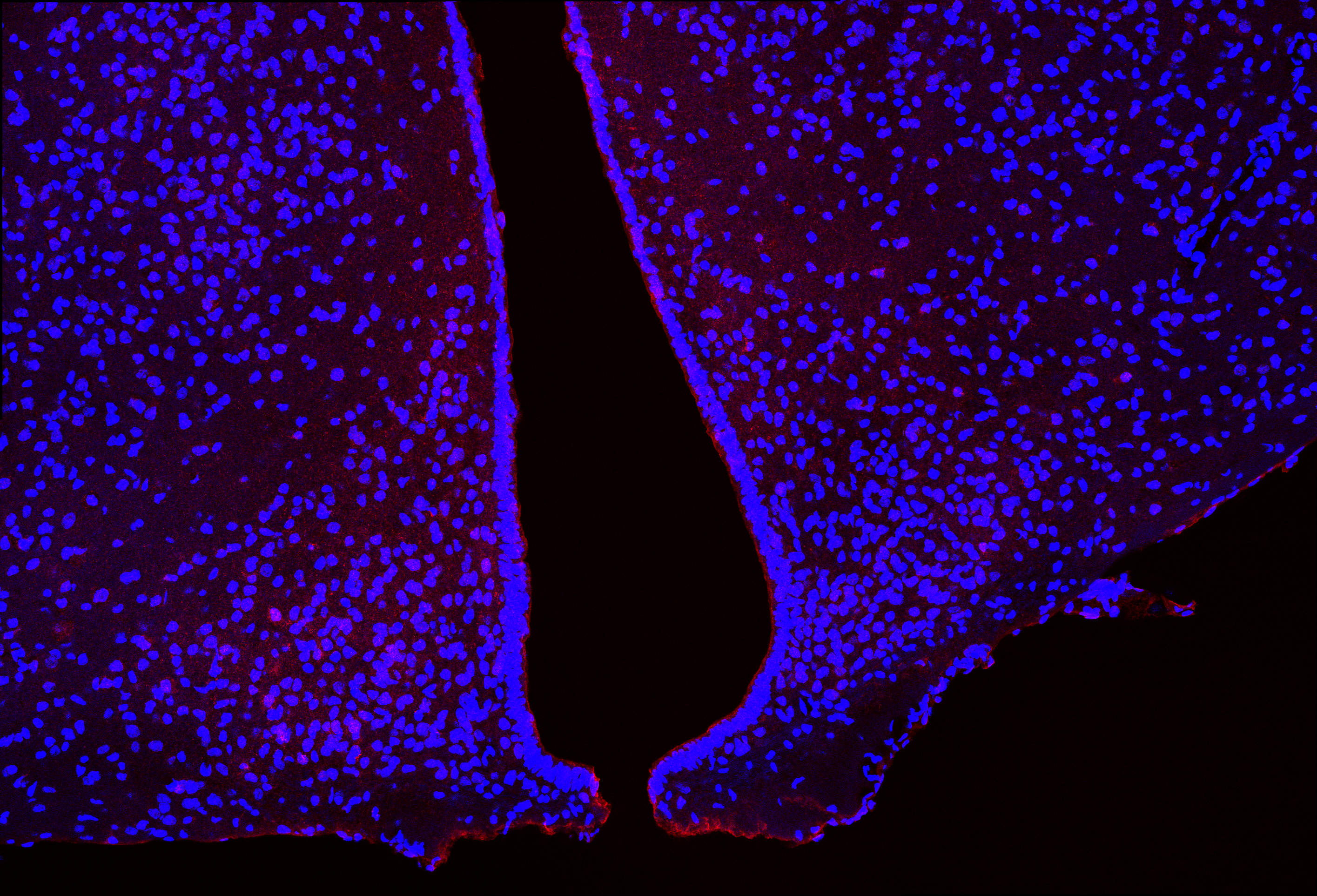

Supplement: Source Data Images Fig. 1 — Source data images. [file 42255_2021_499_MOESM3_ESM.zip › Fig 1b IR-GFP control HFD 0 min.tif]

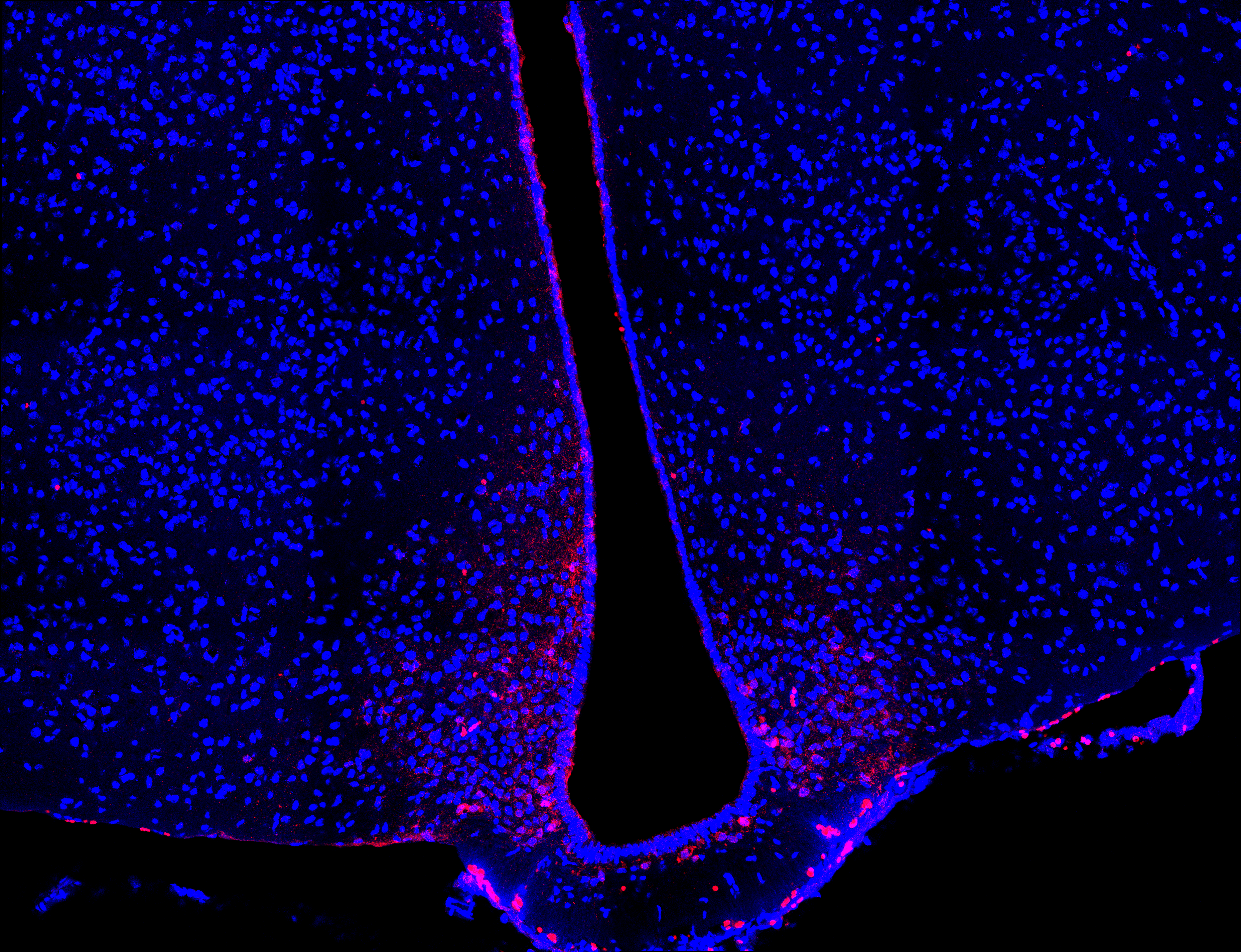

Supplement: Source Data Images Fig. 1 — Source data images. [file 42255_2021_499_MOESM3_ESM.zip › Fig 1h IR-Tan KO 10 min.tif]

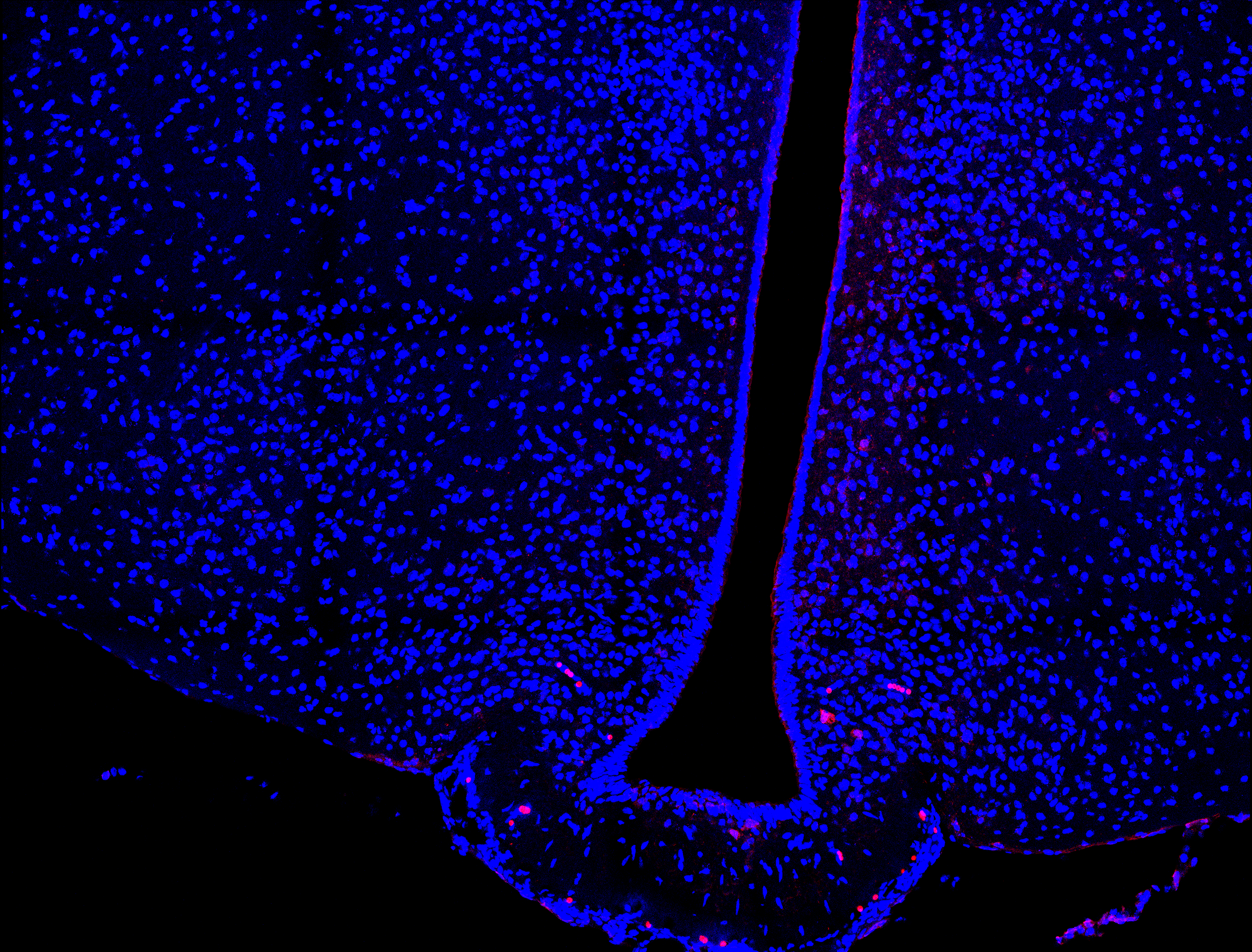

Supplement: Source Data Images Fig. 1 — Source data images. [file 42255_2021_499_MOESM3_ESM.zip › Fig 1h IR-Tan KO 0 min.tif]

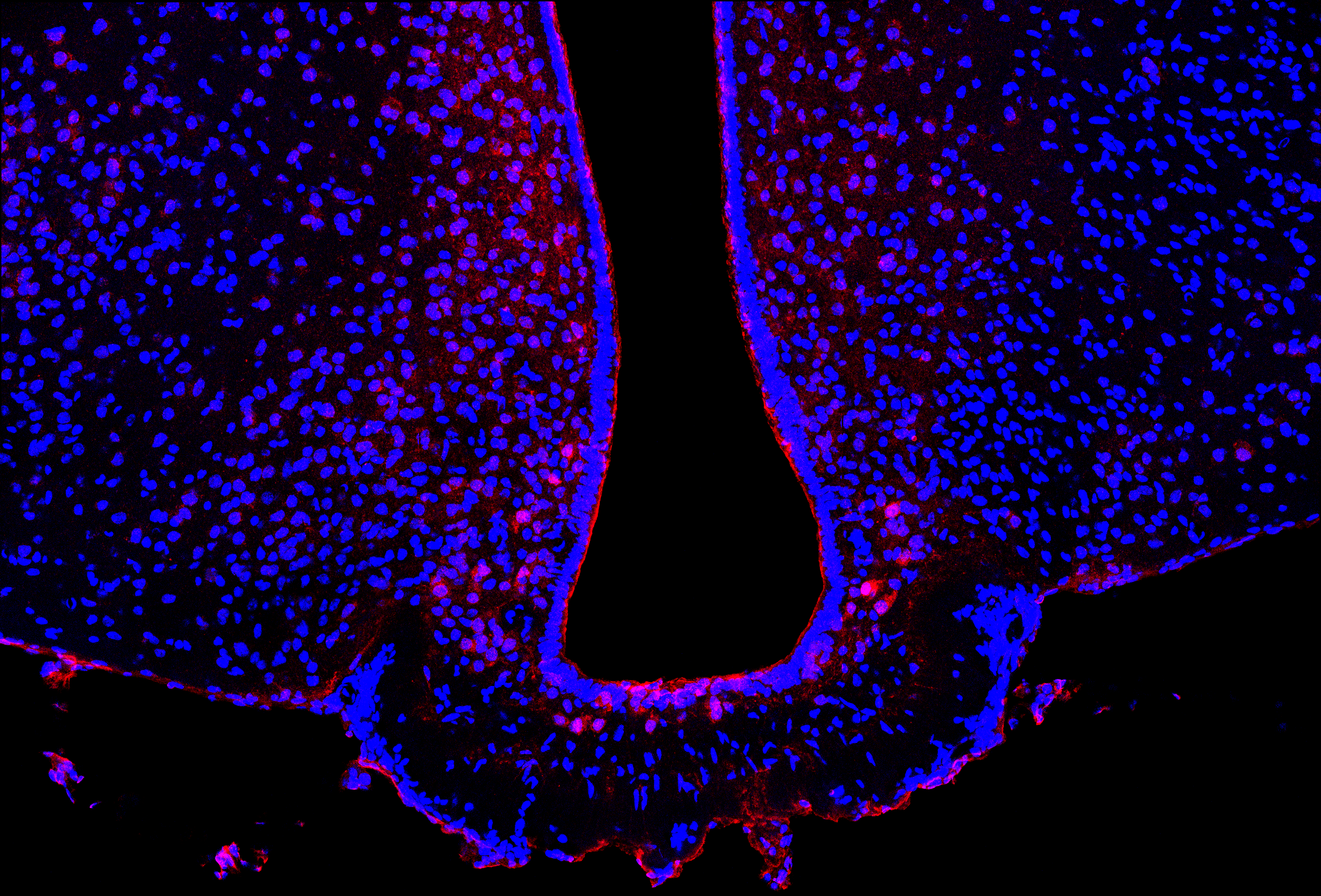

Supplement: Source Data Images Fig. 1 — Source data images. [file 42255_2021_499_MOESM3_ESM.zip › Fig 1h IR-GFP control HFD 5 min.tif]

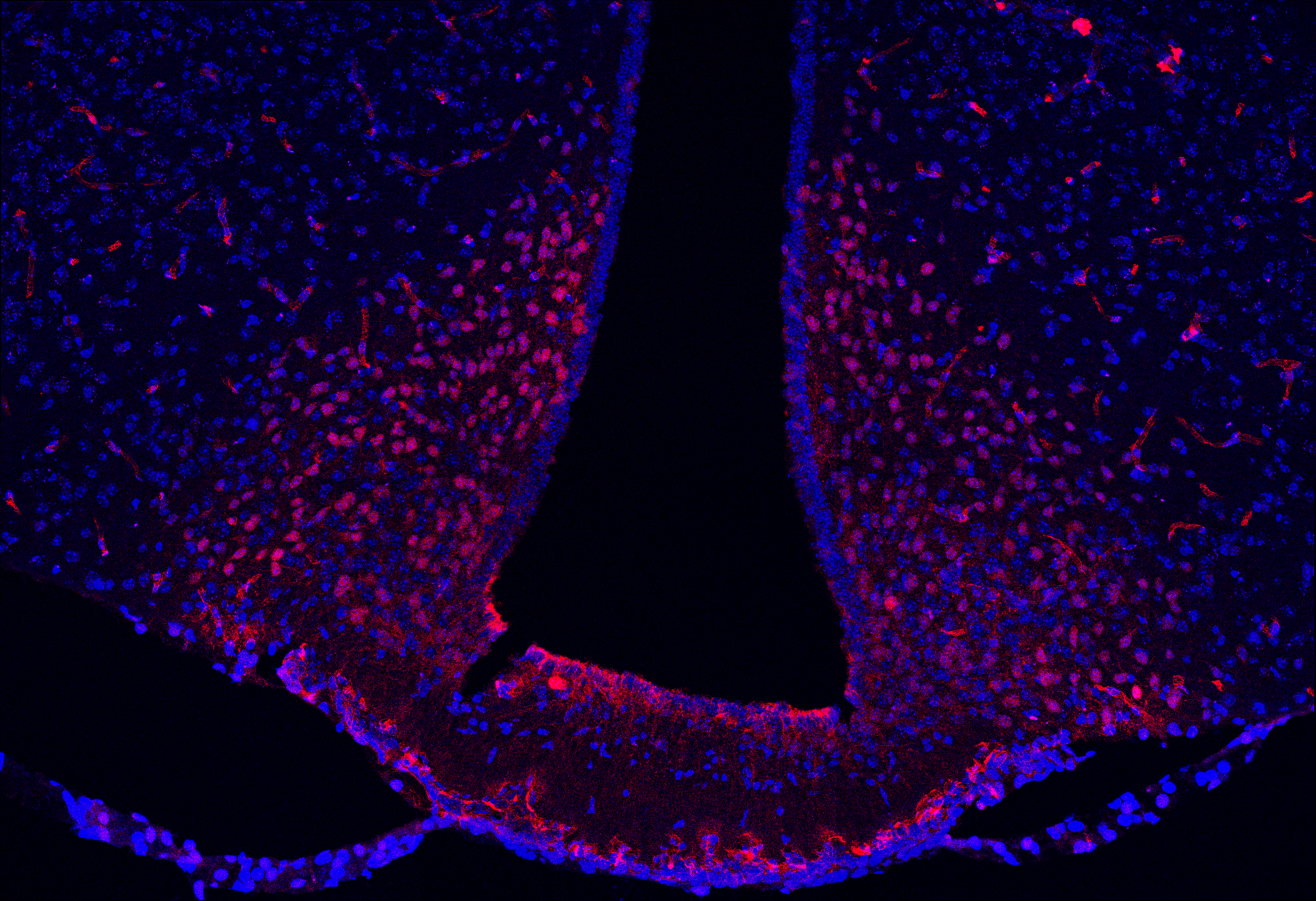

Supplement: Source Data Images Fig. 2 — Source data images. [file 42255_2021_499_MOESM5_ESM.zip › Fig 2 AF488 IR-mKate2 control.tif]

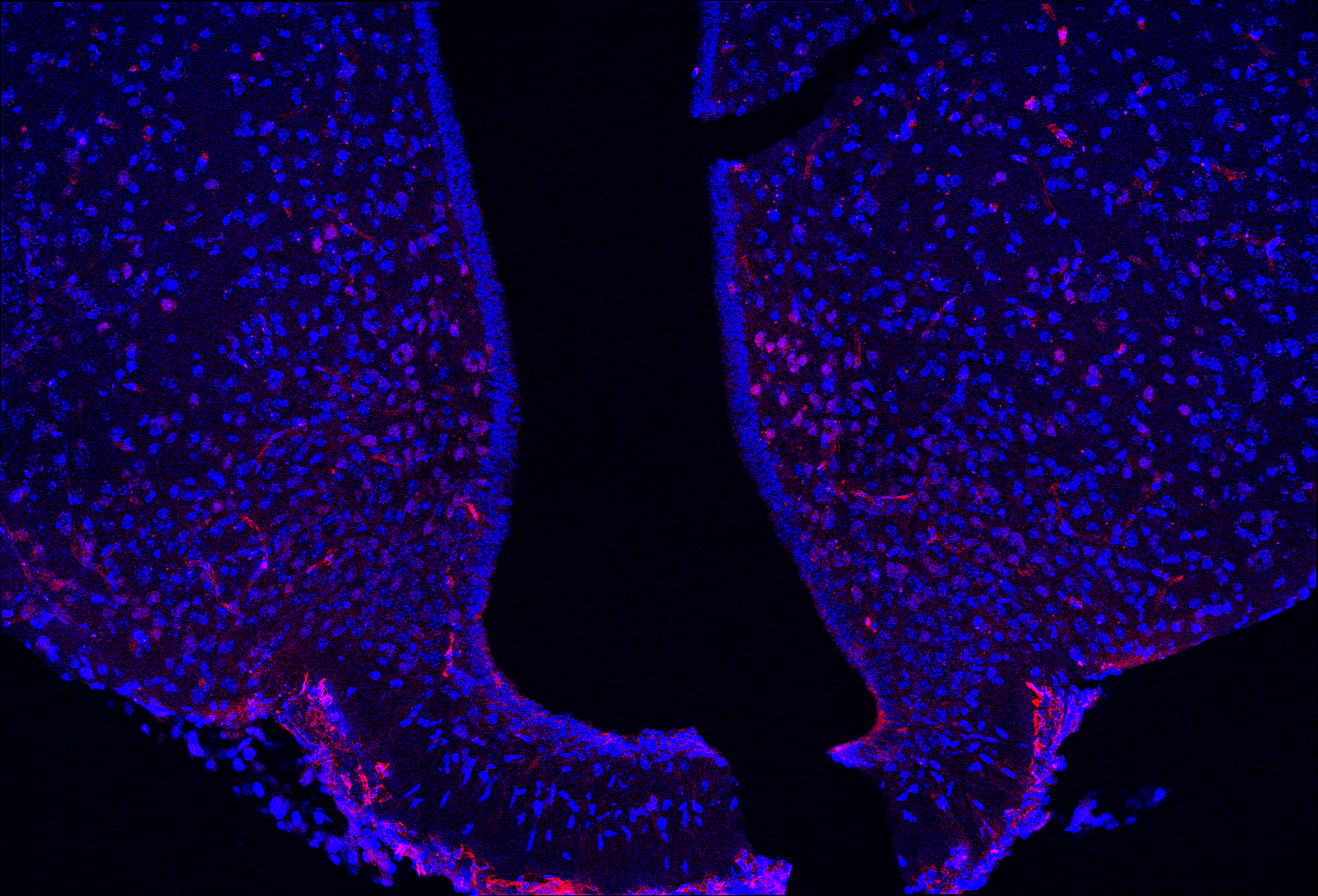

Supplement: Source Data Images Fig. 2 — Source data images. [file 42255_2021_499_MOESM5_ESM.zip › Fig 2 AF488 IR-Tan KO .tif]

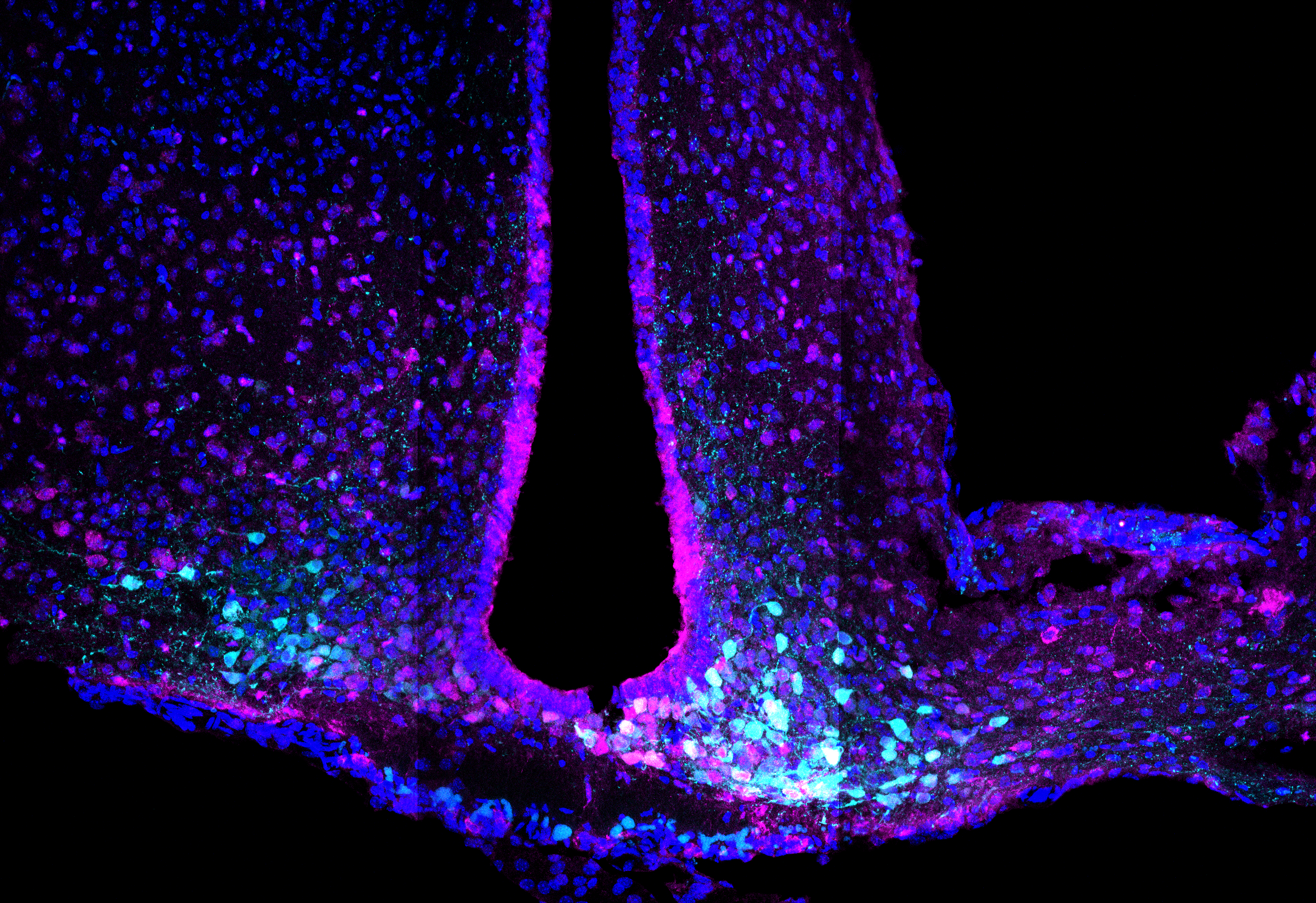

Supplement: Source Data Images Fig. 8 — Source data images. [file 42255_2021_499_MOESM12_ESM.zip › Fig 8c IR-mKate2 Agrp-GCAMP6 control 2.tif]

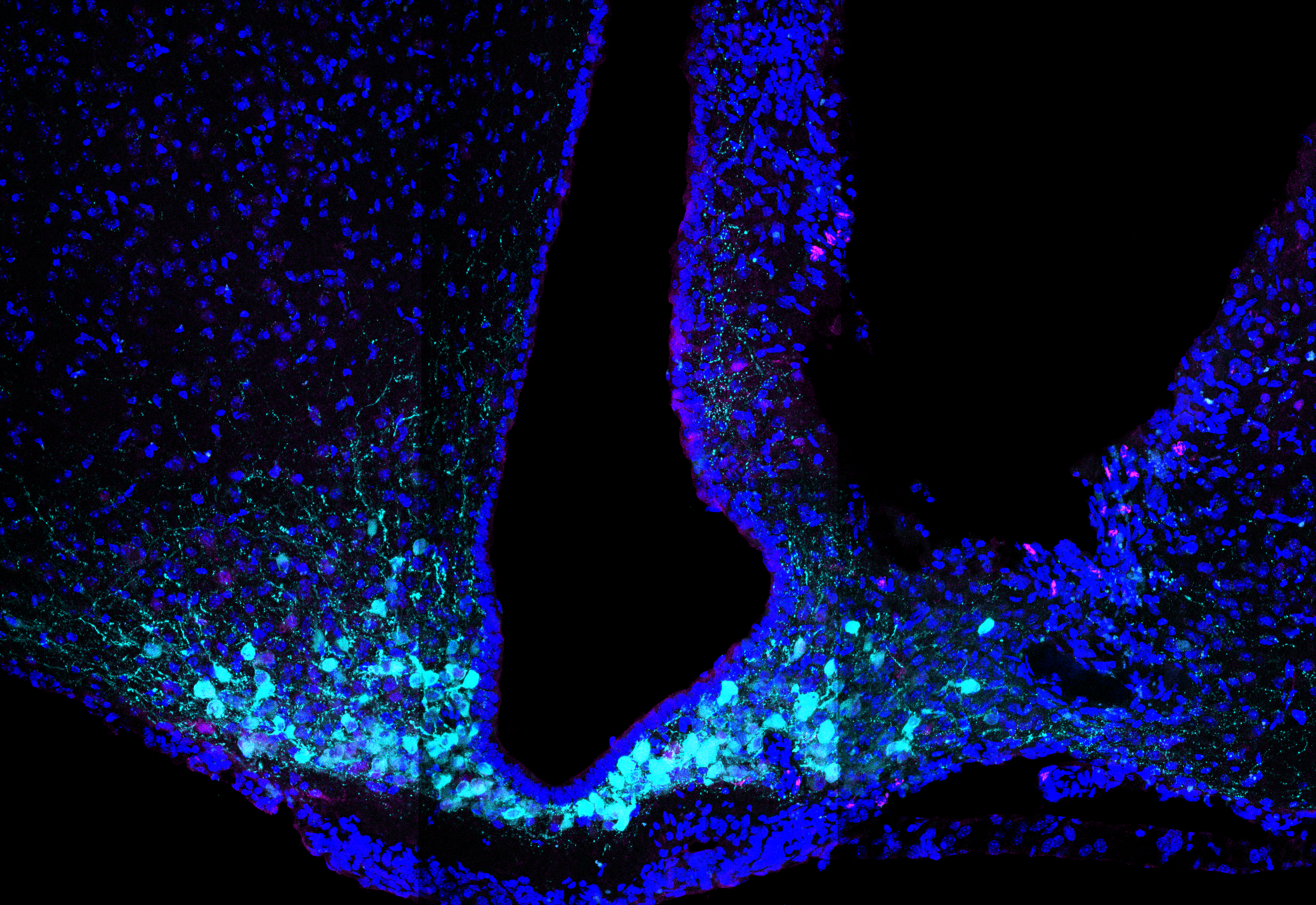

Supplement: Source Data Images Fig. 8 — Source data images. [file 42255_2021_499_MOESM12_ESM.zip › Fig 8c IR-Tan KO Agrp-GCAMP6 2.tif]

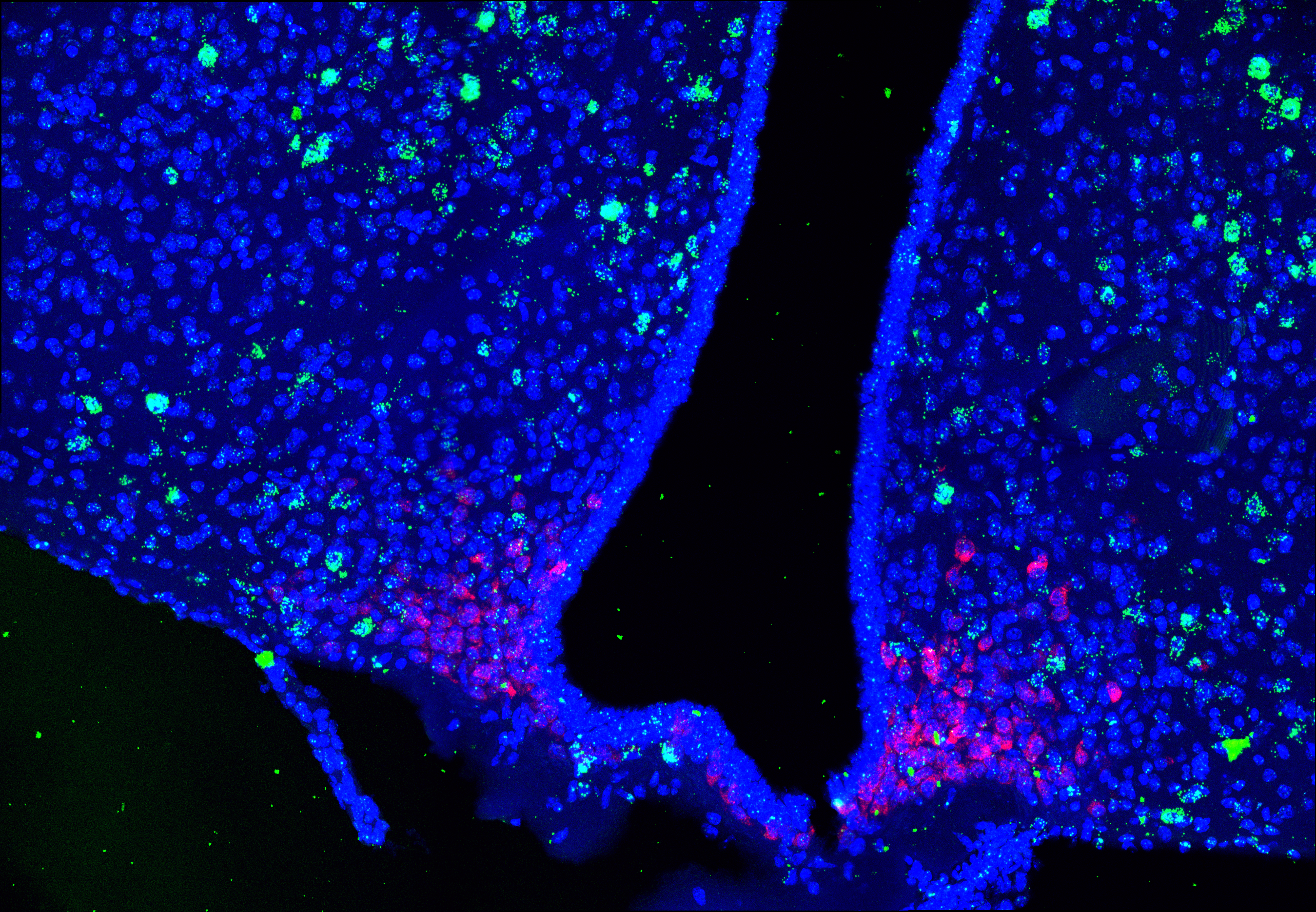

Supplement: Source Data Images Extended Data Fig. 6 — Source data images. [file 42255_2021_499_MOESM18_ESM.zip › ED Fig 6 Refed IR-Tan KO cFOS (green) Agrp (red).tiff]

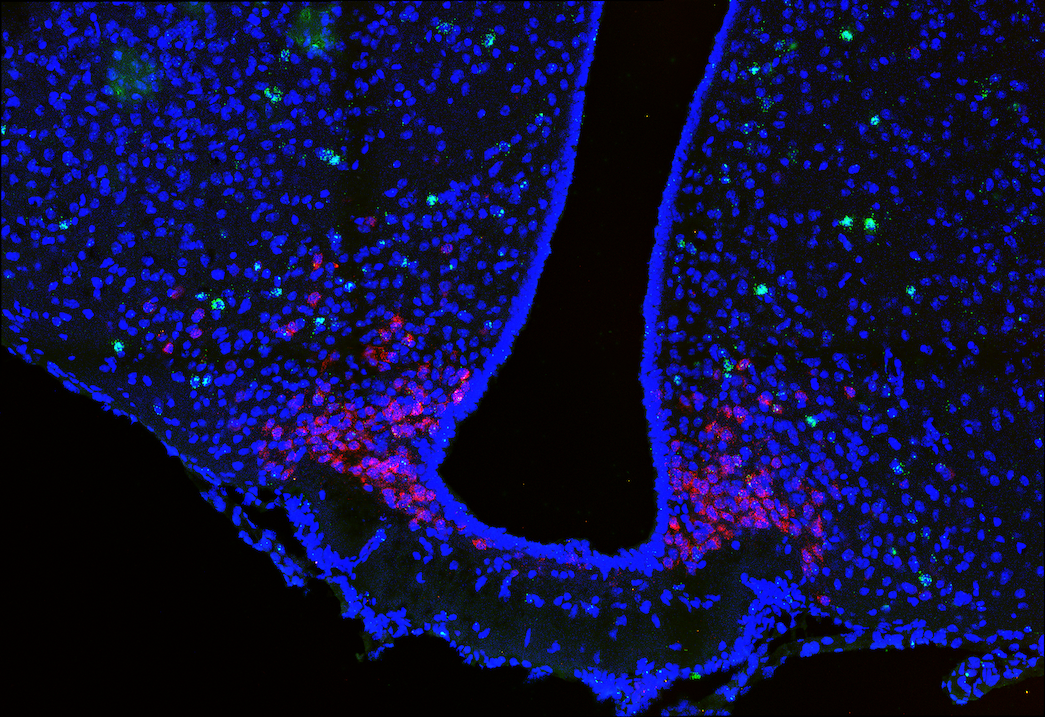

Supplement: Source Data Images Extended Data Fig. 6 — Source data images. [file 42255_2021_499_MOESM18_ESM.zip › ED Fig 6 Fasted IR-GFP control cFOS (green) Agrp (red).tiff]

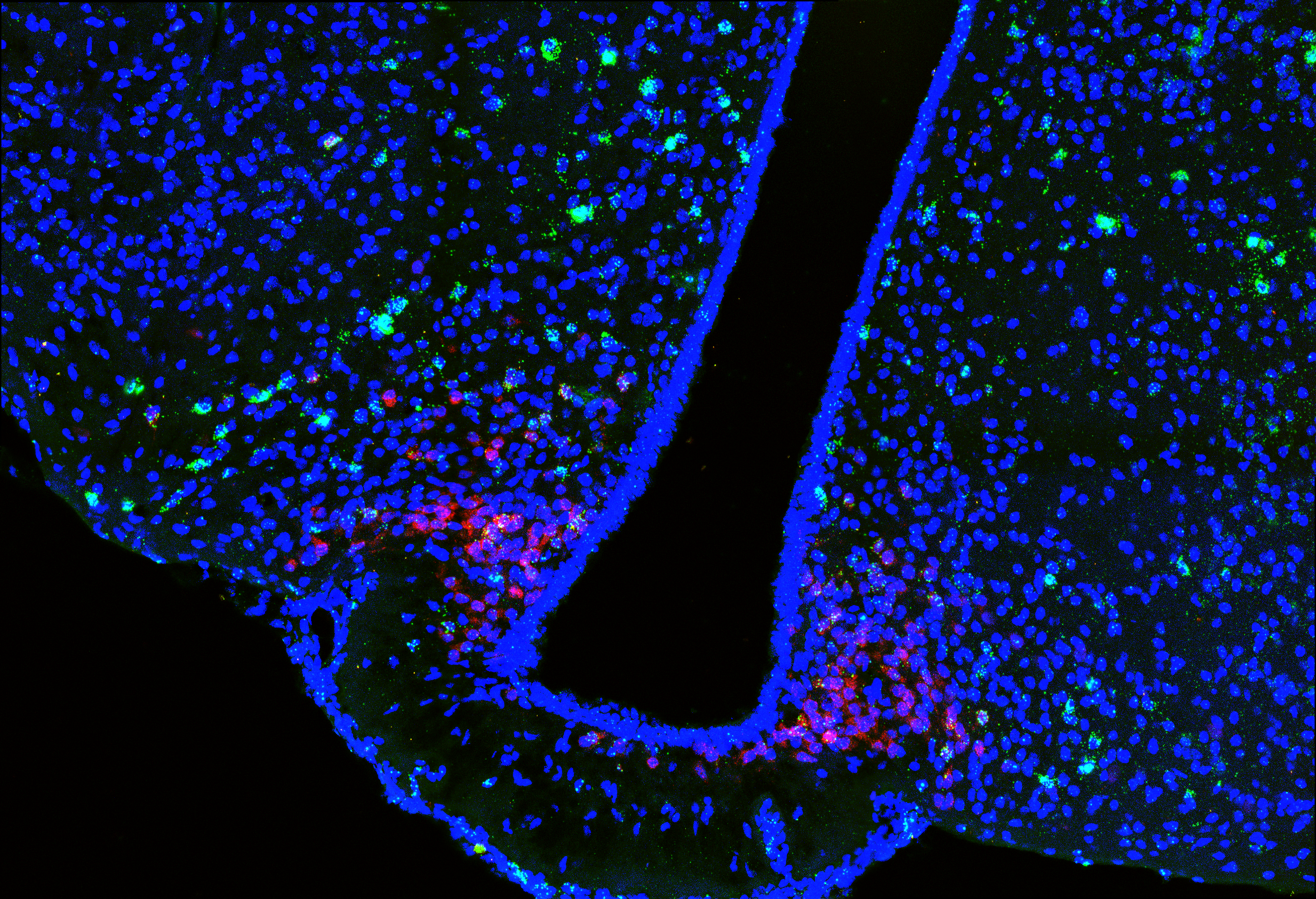

Supplement: Source Data Images Extended Data Fig. 6 — Source data images. [file 42255_2021_499_MOESM18_ESM.zip › ED Fig 6 Fasted IR-Tan KO cFOS (green) Agrp (red).tiff]

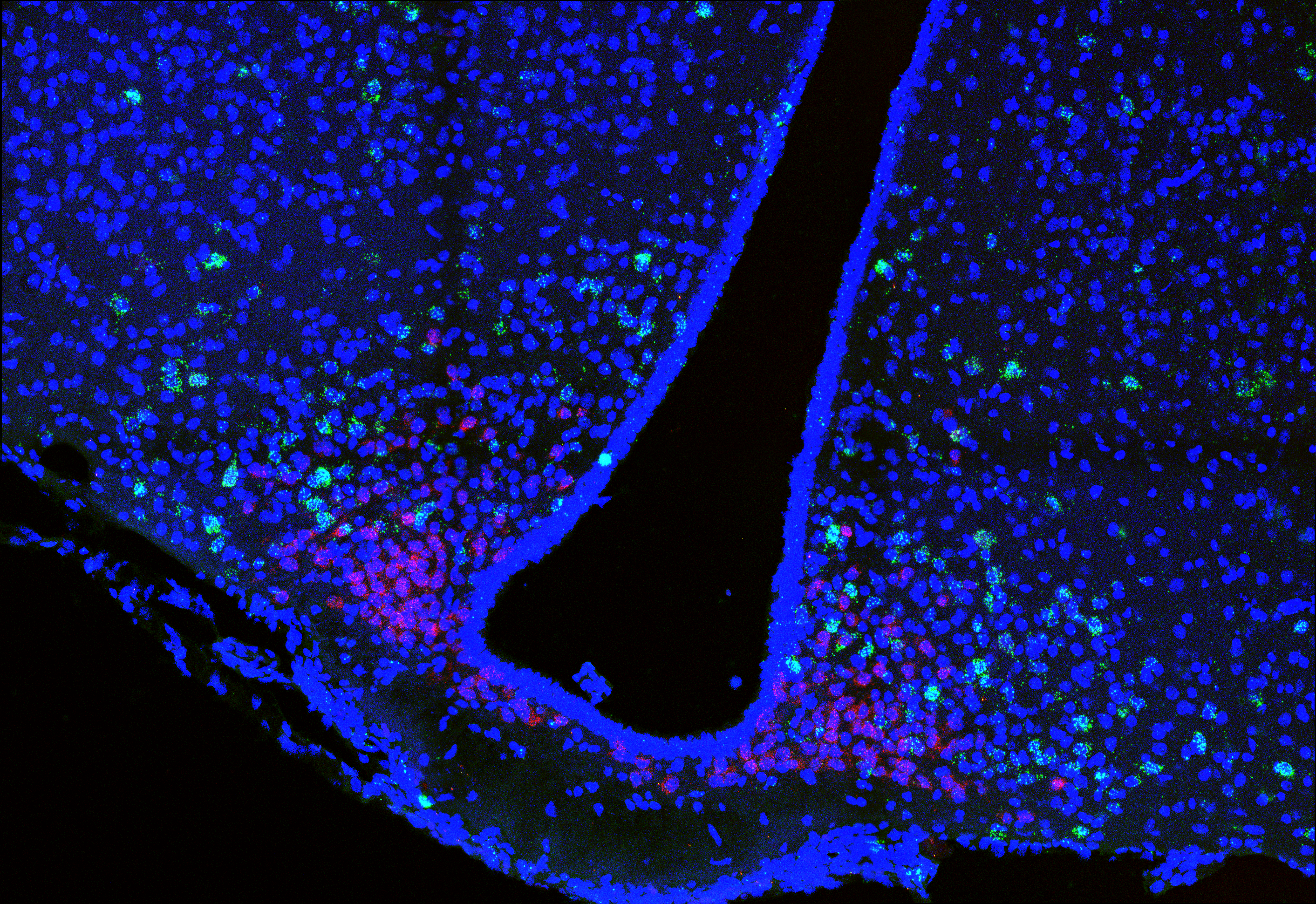

Supplement: Source Data Images Extended Data Fig. 6 — Source data images. [file 42255_2021_499_MOESM18_ESM.zip › ED Fig 6 Refed IR-GFP control cFOS (green) Agrp (red).tiff]

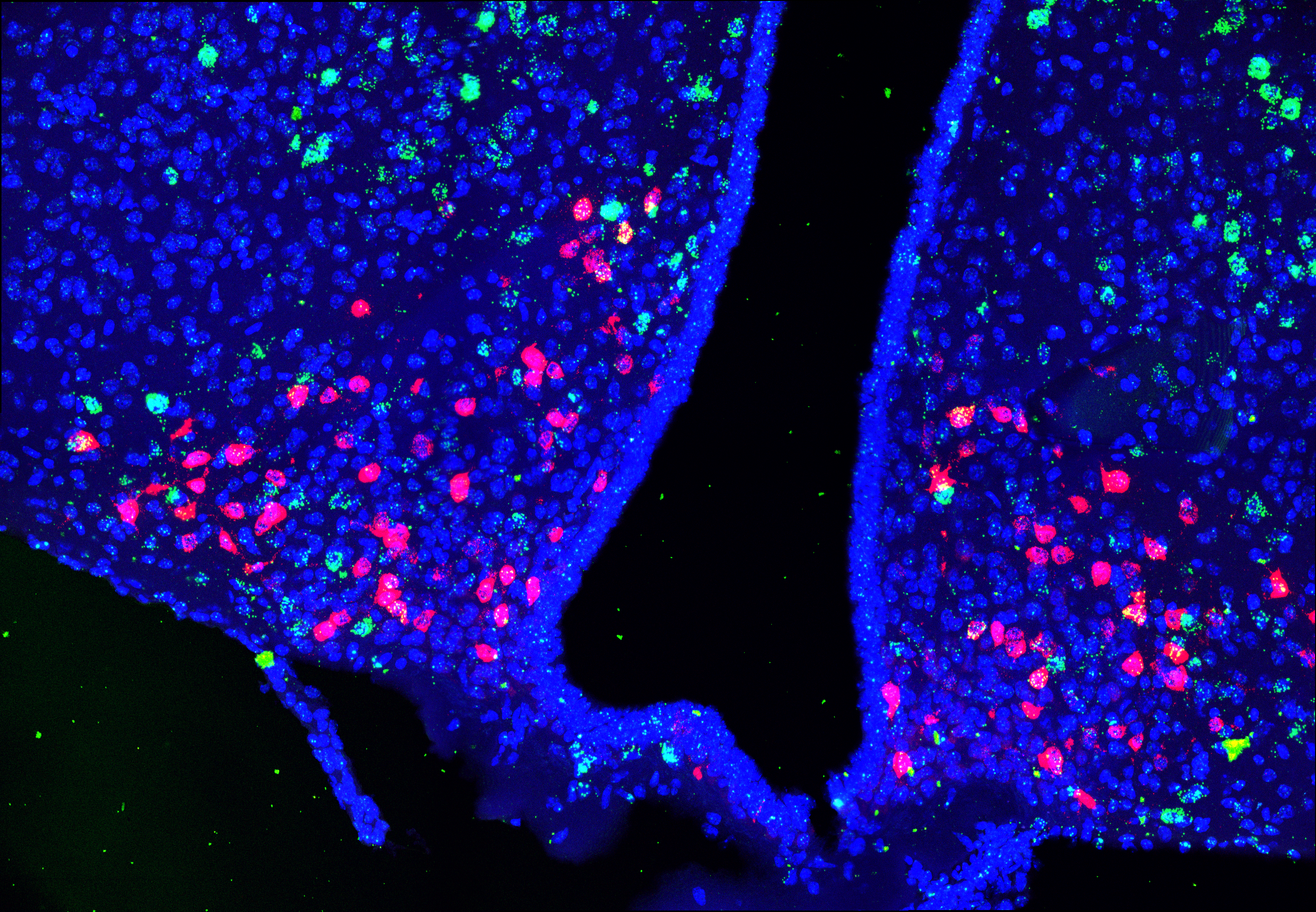

Supplement: Source Data Images Extended Data Fig. 6 — Source data images. [file 42255_2021_499_MOESM18_ESM.zip › ED Fig 6 Refed IR-Tan KO cFOS (green) POMC (red).tiff]

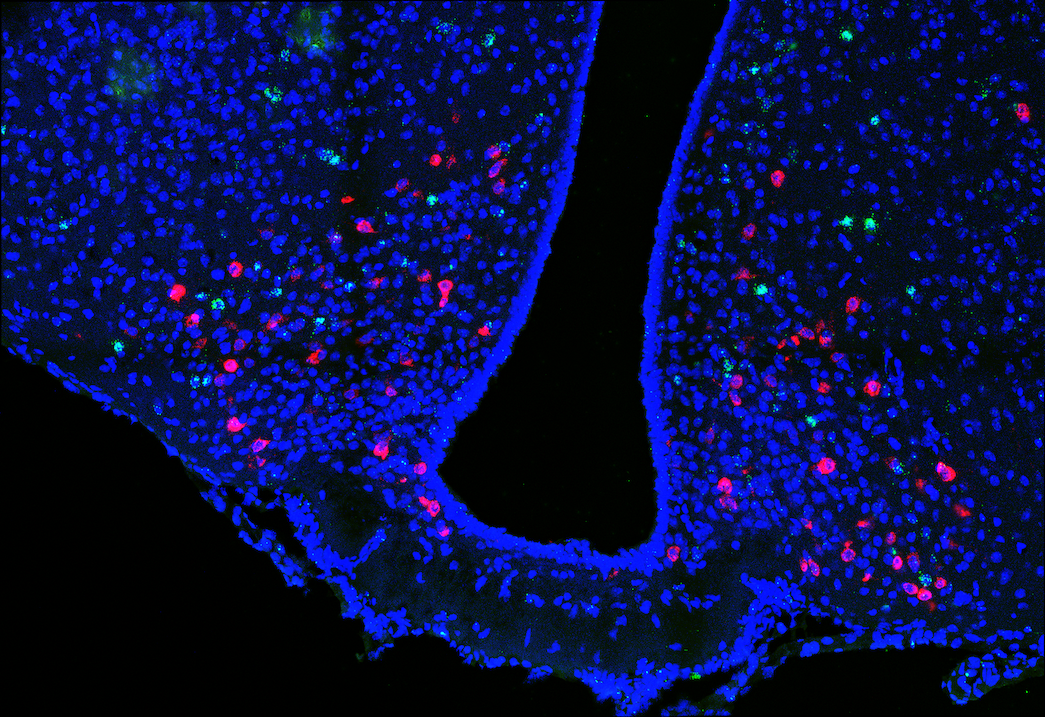

Supplement: Source Data Images Extended Data Fig. 6 — Source data images. [file 42255_2021_499_MOESM18_ESM.zip › ED Fig 6 Fasted IR-GFP control cFOS (green) POMC (red).tiff]

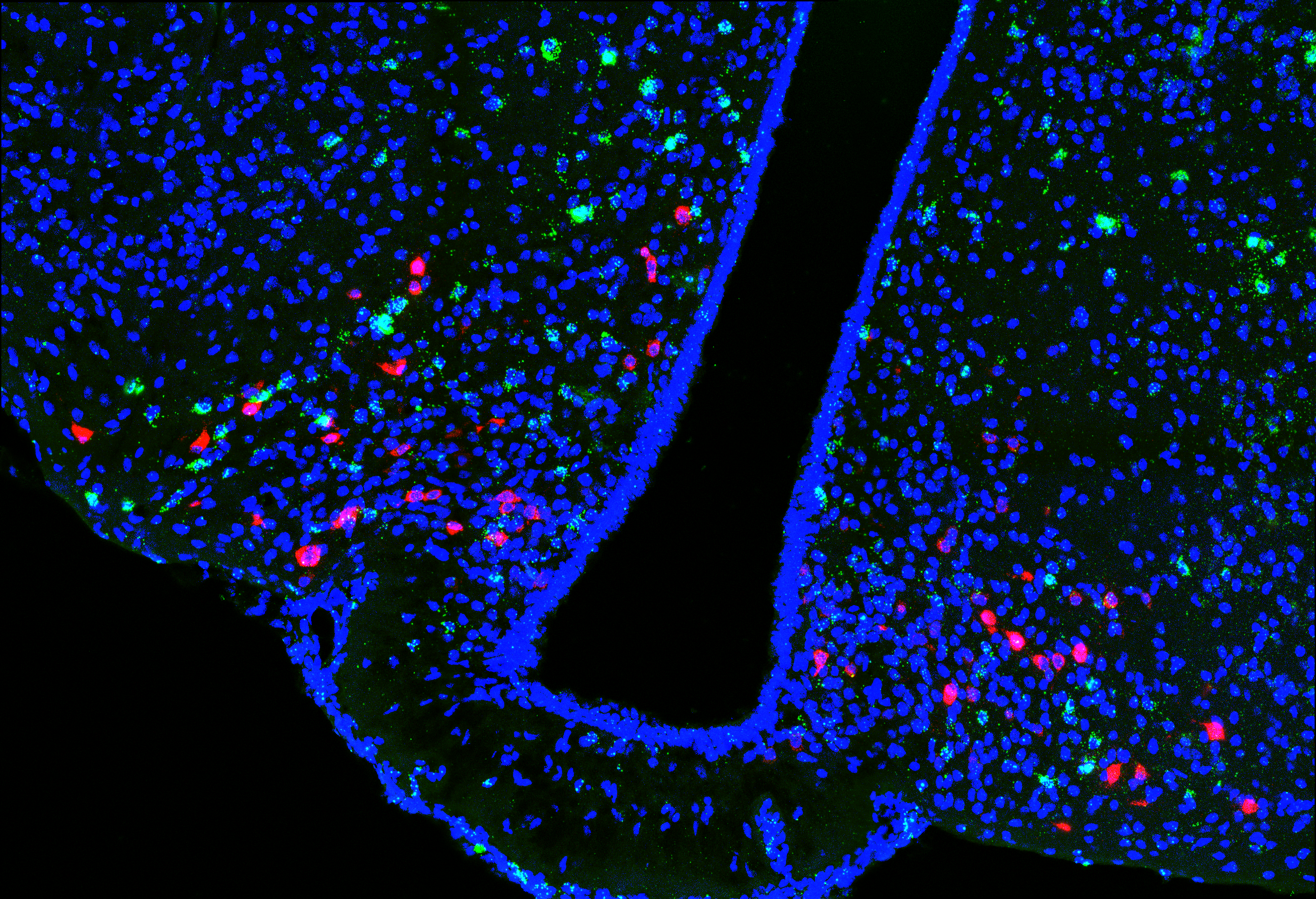

Supplement: Source Data Images Extended Data Fig. 6 — Source data images. [file 42255_2021_499_MOESM18_ESM.zip › ED Fig 6 Fasted IR-Tan KO cFOS (green) POMC (red).tiff]

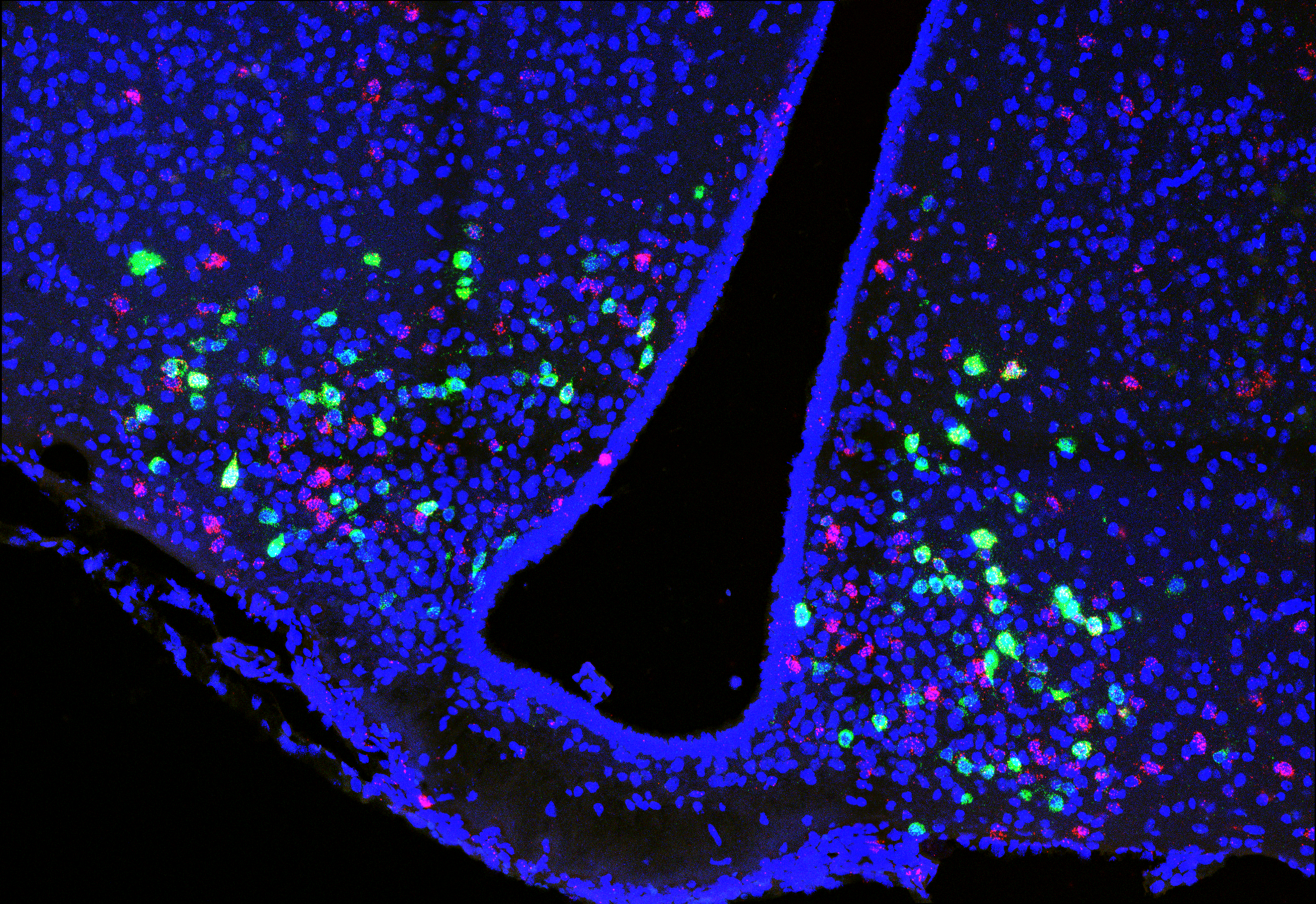

Supplement: Source Data Images Extended Data Fig. 6 — Source data images. [file 42255_2021_499_MOESM18_ESM.zip › ED Fig 6 Refed IR-GFP control cFOS (green) POMC (red).tiff]

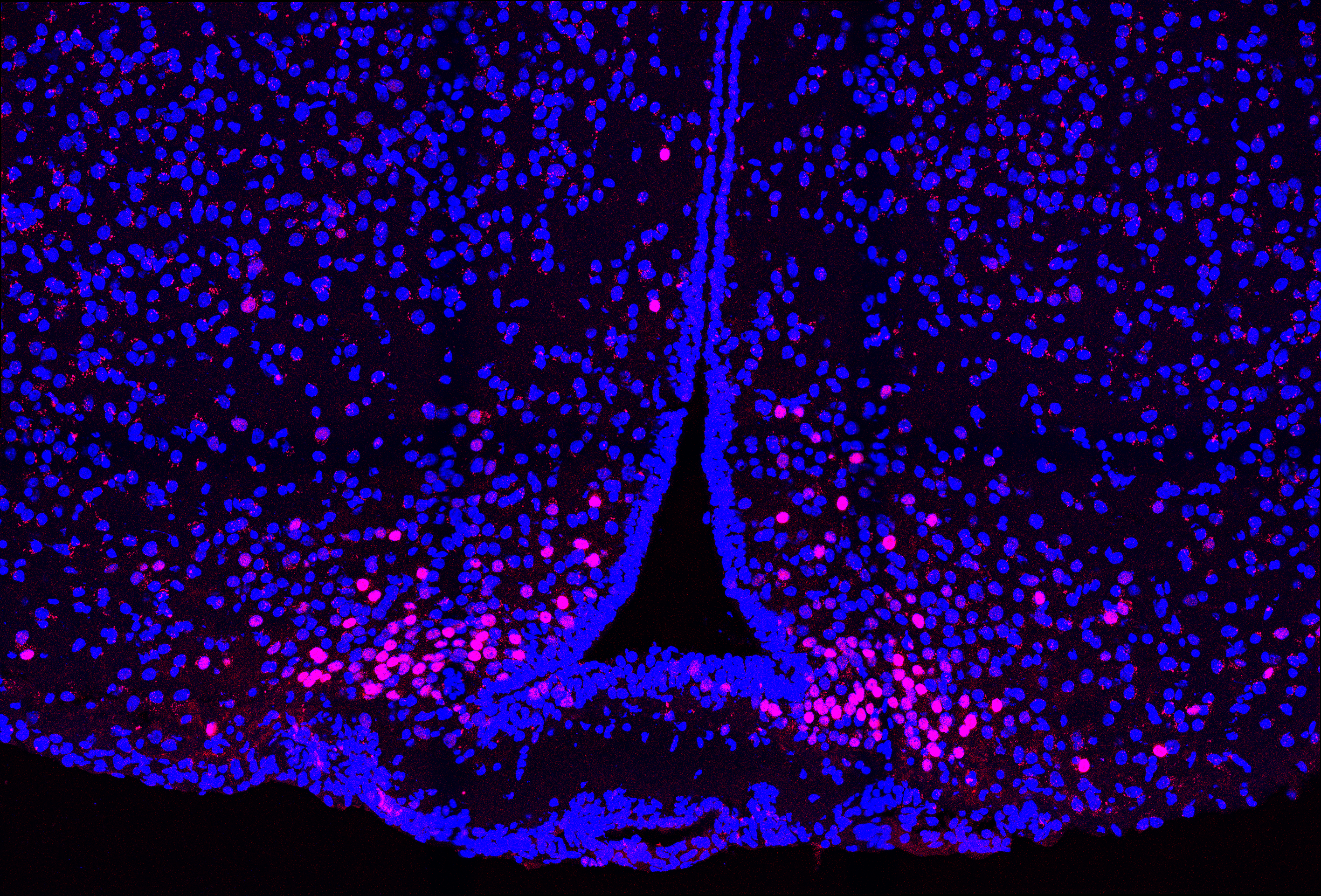

Supplement: Source Data Images Extended Data Fig. 8 — Source data images. [file 42255_2021_499_MOESM21_ESM.zip › ED Fig 8c pSTAT3 IR-Tan KO.tif]

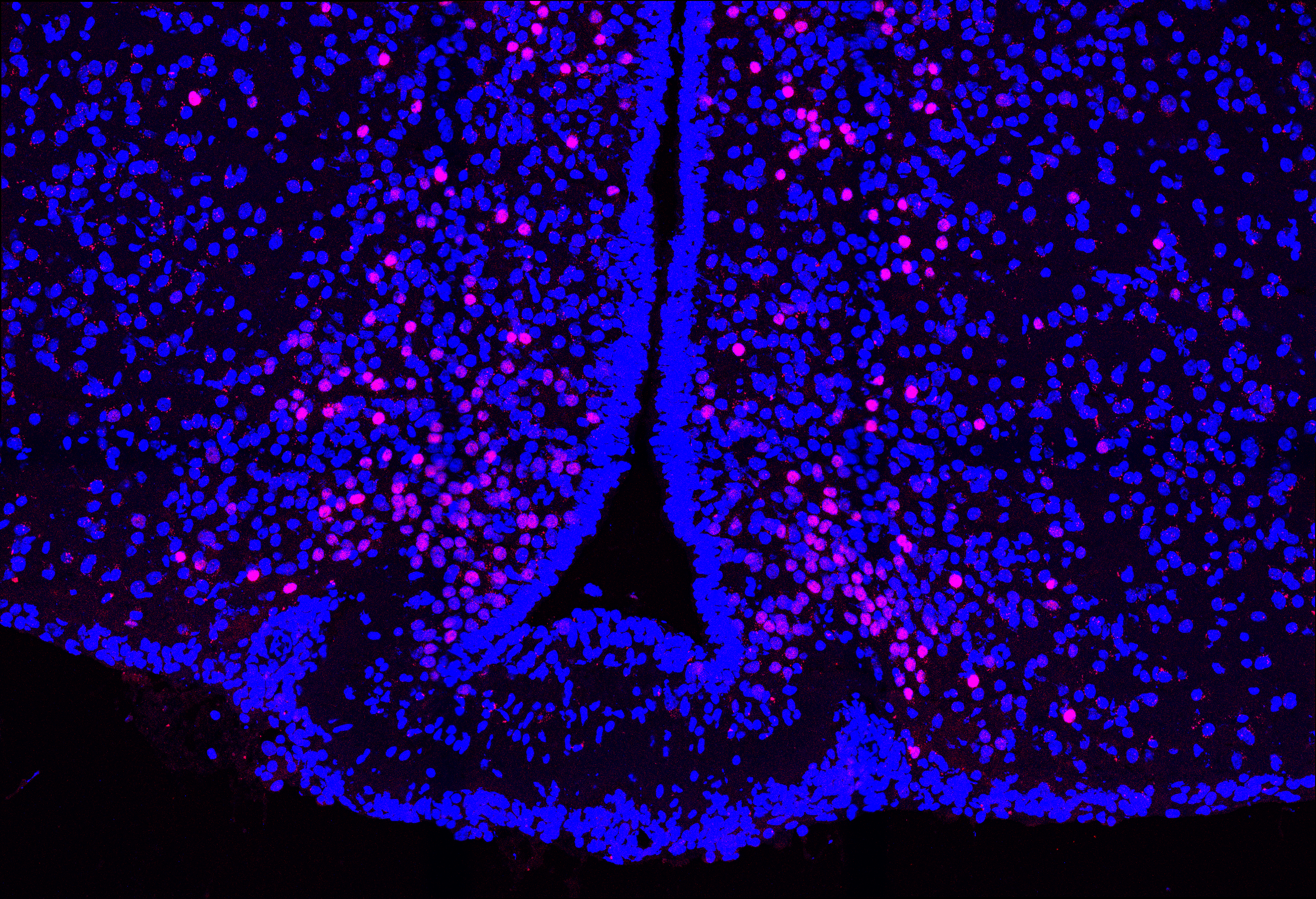

Supplement: Source Data Images Extended Data Fig. 8 — Source data images. [file 42255_2021_499_MOESM21_ESM.zip › ED Fig 8c pSTAT3 IR-GFP control .tif]
