# Supplementary material for: Insulin signalling in tanycytes gates hypothalamic insulin uptake and regulation of AgRP neuron activity
Source: Nat Metab. 2021 Dec 20;3(12):1662–79. doi: 10.1038/s42255-021-00499-0 (PMC8688146; doi:10.1038/s42255-021-00499-0)

Source data

Uncropped images of the gels presented in Suppl. Fig. 3

Hypothalamus

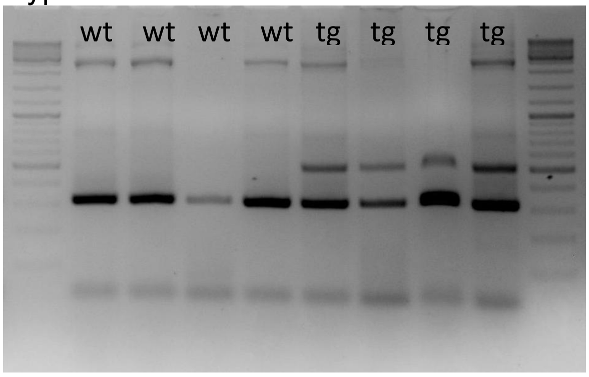

Cortex

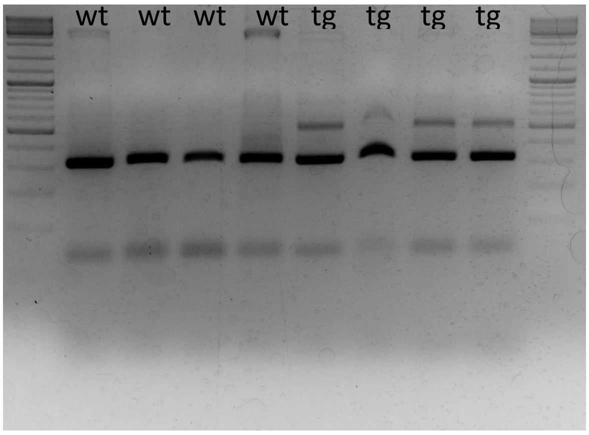

Muscle

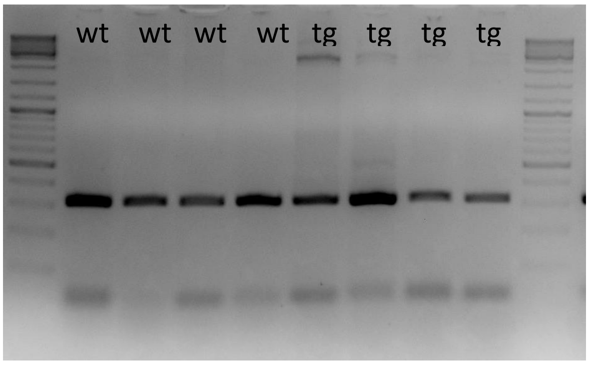

Liver

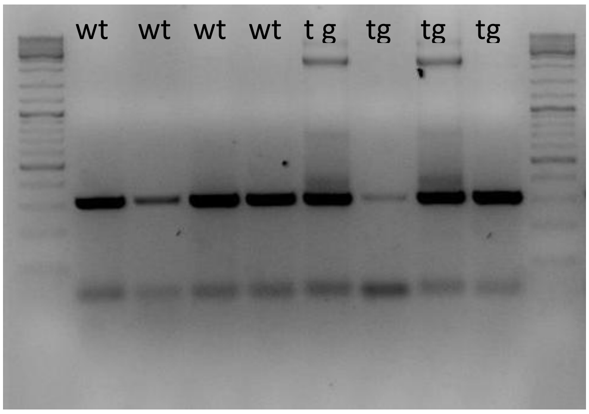

BAT

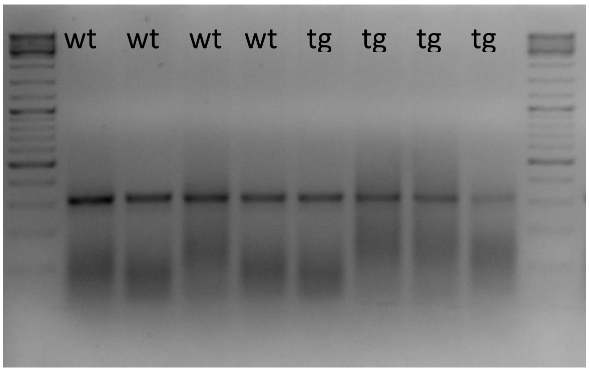

WAT

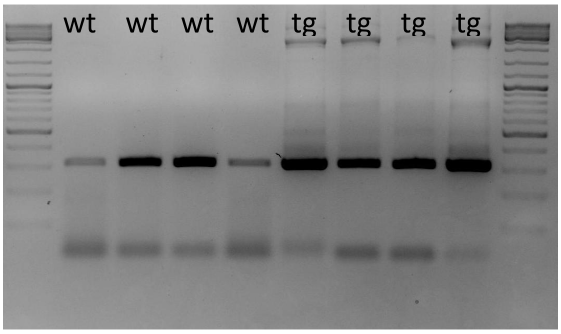

Supplement: Source Data Extended Data Fig. 3 — Unprocessed gels. [file 42255_2021_499_MOESM14_ESM.pdf]
